# Combination of vemurafenib, pleconaril, and AG7404 attenuates enterovirus replication *in vitro* and *in vivo*

**DOI:** 10.1093/narmme/ugaf046

**Published:** 2025-12-23

**Authors:** Erlend Ravlo, Aleksandr Ianevski, Waleria Wolska, Jørn-Ove Schjølberg, María Cámara-Quílez, Ine Emilie Olsen Nordli, Ingrid Bjørnes Sæther, Hilde Lysvand, Valentyn Oksenych, Markus Vähä-Koskela, Sanna Vainionpää, Hanna Seppänen, Teemu Smura, Hanna Vauhkonen, Roni Odai, Adelina Stoyanova, Simeon A Galabov, Angel S Galabov, Pavel Plevka, Magloire Pandoua Nekoua, Didier Hober, Magnar Bjørås, Denis E Kainov

**Affiliations:** Department of Clinical and Molecular Medicine (IKOM), Norwegian University of Science and Technology, 7028 Trondheim, Norway; Department of Clinical and Molecular Medicine (IKOM), Norwegian University of Science and Technology, 7028 Trondheim, Norway; Department of Clinical and Molecular Medicine (IKOM), Norwegian University of Science and Technology, 7028 Trondheim, Norway; Department of Clinical and Molecular Medicine (IKOM), Norwegian University of Science and Technology, 7028 Trondheim, Norway; Department of Microbiology, Oslo University Hospital and University of Oslo, 0372 Oslo, Norway; Department of Clinical and Molecular Medicine (IKOM), Norwegian University of Science and Technology, 7028 Trondheim, Norway; Department of Clinical and Molecular Medicine (IKOM), Norwegian University of Science and Technology, 7028 Trondheim, Norway; Department of Clinical and Molecular Medicine (IKOM), Norwegian University of Science and Technology, 7028 Trondheim, Norway; Department of Clinical and Molecular Medicine (IKOM), Norwegian University of Science and Technology, 7028 Trondheim, Norway; Department of Clinical and Molecular Medicine (IKOM), Norwegian University of Science and Technology, 7028 Trondheim, Norway; Institute for Molecular Medicine FIMM, Helsinki Institute for Life Science, University of Helsinki, 00014 Helsinki, Finland; Translational Cancer Medicine Research Program, Faculty of Medicine, University of Helsinki, 00014 Helsinki, Finland; iCAN Digital Precision Cancer Medicine Flagship, 00014 Helsinki, Finland; Translational Cancer Medicine Research Program, Faculty of Medicine, University of Helsinki, 00014 Helsinki, Finland; iCAN Digital Precision Cancer Medicine Flagship, 00014 Helsinki, Finland; Department of Surgery, Faculty of Medicine, University of Helsinki and Helsinki University Hospital, 00014 Helsinki, Finland; Translational Cancer Medicine Research Program, Faculty of Medicine, University of Helsinki, 00014 Helsinki, Finland; iCAN Digital Precision Cancer Medicine Flagship, 00014 Helsinki, Finland; Department of Surgery, Faculty of Medicine, University of Helsinki and Helsinki University Hospital, 00014 Helsinki, Finland; Department of Virology, University of Helsinki, 00014 Helsinki, Finland; HUS Diagnostic Center, Clinical Microbiology, Helsinki University Hospital, University of Helsinki, 00029 Helsinki, Finland; Department of Virology, University of Helsinki, 00014 Helsinki, Finland; HUS Diagnostic Center, Clinical Microbiology, Helsinki University Hospital, University of Helsinki, 00029 Helsinki, Finland; Department of Experimental Medical Science, Lund University, 22100 Lund, Sweden; The Stephan Angeloff Institute of Microbiology, Bulgarian Academy of Sciences, 1113 Sofia, Bulgaria; The Stephan Angeloff Institute of Microbiology, Bulgarian Academy of Sciences, 1113 Sofia, Bulgaria; The Stephan Angeloff Institute of Microbiology, Bulgarian Academy of Sciences, 1113 Sofia, Bulgaria; Central European Institute of Technology, Masaryk University, 62500 Brno, Czech Republic; Laboratoire de virologie ULR3610, Univ Lille et CHU Lille, 59000 Lille, France; Laboratoire de virologie ULR3610, Univ Lille et CHU Lille, 59000 Lille, France; Department of Clinical and Molecular Medicine (IKOM), Norwegian University of Science and Technology, 7028 Trondheim, Norway; Department of Microbiology, Oslo University Hospital and University of Oslo, 0372 Oslo, Norway; Centre for Embryology and Healthy Development (CRESCO), University of Oslo, Oslo 0373, Norway; Department of Clinical and Molecular Medicine (IKOM), Norwegian University of Science and Technology, 7028 Trondheim, Norway; Translational Cancer Medicine Research Program, Faculty of Medicine, University of Helsinki, 00014 Helsinki, Finland

## Abstract

Enteroviruses infect multiple human tissues and cause diseases including meningitis, the common cold, myocarditis, pancreatitis, hepatitis, poliomyelitis, sepsis, type 1 diabetes, hand, foot, and mouth disease. Despite this burden, no antiviral therapy has been approved to date. Progress has been limited by the structural and topical diversity of enteroviruses because many variants are intrinsically insensitive to candidate agents and sensitive strains develop resistance rapidly. Here, we report that the approved anticancer drug vemurafenib inhibited replication of some tested enteroviruses in cell cultures. Passage of echovirus EV1 and coxsackievirus CVB5 for six cycles in cell culture yielded vemurafenib-resistant virus variants harboring mainly missense mutations in the viral 3A and VP1 proteins, underscoring the need for combination therapy. We therefore evaluated cocktails, combining vemurafenib with the VP1 inhibitor pleconaril and the 3C protease inhibitor AG7404. In cell culture, the cocktails suppressed replication of all seven tested enteroviruses. The combination was also effective in human pancreatic, retinal, and brain organoids. In infected mice, the triple regimen reduced viral titers in the pancreas. These findings support multi-stage targeting of the enterovirus life cycle as a promising path toward broadly active therapeutic cocktails.

## Graphical Abstract

**Graphical Abstract:**
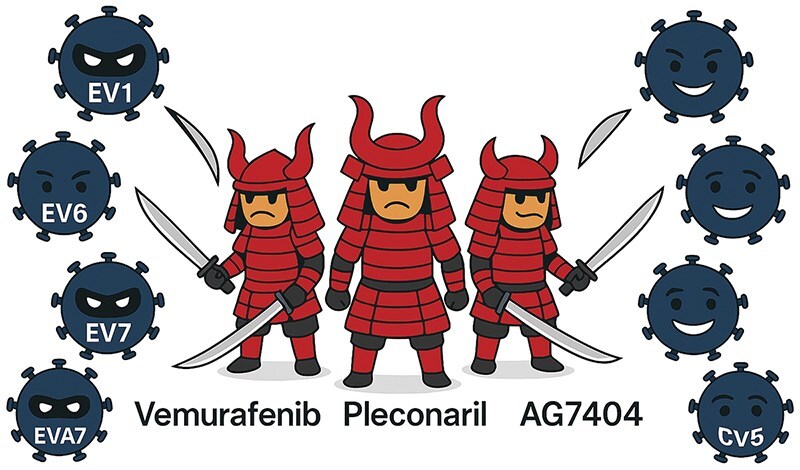

## Introduction

Enteroviruses are globally prevalent pathogens responsible for a wide range of diseases. These diseases can range from the common cold to severe conditions such as meningitis, myocarditis, pancreatitis, sepsis, poliomyelitis, and hand-foot-and-mouth disease (HFMD) [1]. Additionally, the association of picornaviruses with chronic diseases such as asthma, allergies, and Type 1 diabetes (T1D) underscores their significant clinical impact [2–4]. Recent outbreaks of echovirus 11 (EV11) have caused sepsis and death in neonates in Europe [5]. In 2023, Enterovirus A71 (EVA71) caused a severe HFMD outbreak in Asia [6]. Additionally, coxsackievirus CVA13 caused an HFMD outbreak in Africa [7].

Despite the widespread impact of enteroviral infections, there is a lack of approved anti-enteroviral drugs [8–10]. Many antivirals are in development [11, 12]. They could be grouped based on their targets. Virus-directed antivirals target conserved viral structures or enzymes and block key steps of the enterovirus life cycle. Capsid binders stabilize the particle or prevent receptor engagement/uncoating—classically at the VP1 hydrophobic pocket (pleconaril, vapendavir, and pirodavir) or at alternative sites such as the VP1–VP3 interprotomer pocket (benzoic/sulfonamide derivatives) and the 5-fold axis (MADL-385/CB-30, suramin). Viral 3C protease (3Cpro) and 2Apro inhibitors block viral polyprotein processing (rupintrivir, AG7404, NK-1.9k, cyanohydrin, telaprevir, and CW-33). Polymerase (3Dpol) inhibitors include nucleosides/nucleotides that terminate or attenuate error-prone replication (gemcitabine, LY2334737, sofosbuvir, 4′-azidocytidine, favipiravir, NITD008, remdesivir, and mindeudesivir) and non-nucleosides that block elongation or VPg uridylation (GPC-N114, BPR-3P0128, and DTriP-22). Additional viral targets include 2C ATPase (fluoxetine S-enantiomer; dibucaine-derived 6aw/6i; JX040; R523062). Emerging biologics and nucleic-acid modalities complement these: IVIG, neutralizing mAbs, si/shRNAs against conserved 2A/2C/3C/3Dpol/5′UTR, CRISPR–Cas13 antivirals targeting viral genomes, and antiviral peptides.

Host-directed antivirals exploit the virus’s reliance on cellular pathways. Central is the PI4KB–PI4P–OSBP lipid-shuttle axis that builds replication organelles. OSBP inhibitors include OSW-1, itraconazole, posaconazole, TTP-8307/T-00127-HEV2, and PI4KB inhibitors include MDL-860/allosteric C646, PIK93, T-00127-HEV1, “compound 10,” and perhaps vemurafenib. Nucleotide biosynthesis can be throttled with DHODH inhibitors, such as RYL-634 and FA-613. Entry/attachment can be blocked using heparan-sulfate mimetics and lectins (HTA-22, heparin/pentosan, and lactoferrin), whereas uncoating can be inhibited with cyclosporine A, HL051001P2, and CypA-11. Additionally virus-host interaction could be modulated with ACA that target AP2M1, emetine, homoharringtonine, anisomycin, or cycloheximide that target translation machinery, type I IFNs, R837, and GS-9620 that target innate immune responses,and Torin2 and LY-55 that interfere with autophagy/mTOR signaling [13].

The virus-directed inhibitors face low coverage of enteroviruses and the rapid emergence of drug-resistant virus variants. Host-directed antivirals often have a broader spectrum and higher resistance barriers but are associated with higher drug toxicity, particularly in children [14]. To tackle these issues, antiviral drugs are combined into cocktails [8, 15, 16]. Recently, we tested several drug combinations against enterovirus infections *in vitro*. We showed that the combinations of vemurafenib with translation inhibitors (emetine, homoharringtonine, anisomycin, and cycloheximide), VP1 binder (pleconaril and vapendavir), 3Cpro inhibitor (rupintrivir and AG7404), and 3Dpol inhibitors (remdesivir and mindeudesivir) were synergistic in cell culture, suggesting these partners could lower the required dose of vemurafenib [15, 17–19]. In addition, we showed that triple combinations of vemurafenib–pleconaril–rupintrivir, pleconaril–rupintrivir–remdesivir, and pleconaril–AG7404–mindeudesivir further improved synergy in cell cultures [17, 18]. Importantly, the combination delayed the emergence of antiviral drug resistance, distinguishing it from single drugs and two-drug cocktails.

Here, we found that a cocktail consisting of orally available vemurafenib, pleconaril, and AG7404 inhibited the replication of EV1, EV6, EV7, EV11, EVA71, CVB5, and CVB6 in human retinal pigment epithelium (RPE), rhabdomyosarcoma (RD), and pancreatic MiaPaCa-2 (MP2) cells at noncytotoxic concentrations. The combination also protected human pancreatic organoids from virus-mediated cell death, as well as reduced viral replication in retinal and brain organoids and in the pancreas of mice. Thus, we discovered a combination of orally available small molecules suitable for further preclinical trials and clinical trials against a broad range of enterovirus infections.

## Materials and methods

### Small molecules, viruses, and cells

Supplementary Table S1 lists compounds used in the study, their suppliers, and catalog numbers. To obtain 10 mM stock solutions, compounds were dissolved in dimethyl sulfoxide (DMSO; Sigma–Aldrich, Germany) or water. The solutions were stored at −20°C until use.

Supplementary Table S2 lists viruses used in the study, and their sources. EV1, EV6, EV11, and CVB5 were amplified in a monolayer of A549 cells, whereas EVA71, CVB6, and EV7 were amplified in RD cells. The diabetogenic strain CVB4 E2 (provided by Ji-Won Yoon, Julia McFarlane Diabetes Research Center, Calgary, Alberta, Canada) was propagated in HEp-2 cells [20, 21]. The CVB1 Connecticut 5 strain was passaged twice intracerebrally in newborn ICR mice and prepared as a clarified suspension. Infectious titer was 1.2 × 10⁸ TCID₅₀ ml⁻¹; the mouse lethal dose 50 % (MLD₅₀) was determined by the Reed–Muench method. Virus stocks were stored at −80°C.

Supplementary Table S3 lists cells used in the study and their sources. A549, MP2, and RD cells were grown in Dulbecco’s Modified Eagle’s medium (DMEM; Gibco, Paisley, Scotland), and RPE cells were grown in DMEM-F12 supplemented with 100 U/ml penicillin, 100 μg/ml streptomycin (pen/strep; Lonza, Cologne, Germany), and 10% heat-inactivated fetal bovine serum (FBS; Lonza, Cologne, Germany). All cells were cultured at 37ºC with 5% CO_2_, 95% humidity, and passaged using 0.05% (v/v) Trypsin/EDTA (Gibco). Cells were tested mycoplasma-negative throughout the work (MycoAlert Mycoplasma Detection Kit, Lonza).

### Virus infection and median tissue culture infectious dose calculation (TCID50)

The infection of RD, MP2, and RPE cells was done in growth media containing 2% FBS. Viral samples (1 µl) at seven 10-fold dilutions were added to 99 µl of virus growth medium. The mixture was added to 96‐well plates containing approximately 4 × 10^4^ cells/well. At 48 h post-infection, cell viability was measured with Cell Titer Glo (CTG; Promega) assay and the median tissue culture infectious dose 50 (TCID_50_) was calculated as described previously [22].

### Calculation of DSS, EC_50_, and CC_50_ values

Drugs were added to cells in 3-fold serial dilutions. Cells were infected with viruses (100 TCID_50_) or mock. The cell viability was measured using CTG assay after 48 h of infection. A drug sensitivity score (DSS) was calculated as a normalized version of the standard area under dose–response curve (AUC), with the baseline noise subtracted, and the normalized maximal response at the highest concentration (often corresponding to off-target toxicity):


(1) \begin{eqnarray*}
DSS = \frac{{AUC - t\left( {{{x}_{max}} - {{x}_{min}}} \right)}}{{\left( {100 - t} \right)\left( {{{x}_{max}} - {{x}_{min}}} \right){{{\log }}_{10}}\textit{Amin}}},
\end{eqnarray*}

where activity threshold *t* equals 10%, and DSS is in the 0–50 range [18, 19, 23, 24]. The EC_50_ and CC_50_ were also calculated.

### Experiments with human organoid cultures

Pancreatic cancer organoid cultures have been described [25]. Organoids were maintained in matrigel domes and complete feeding medium [26]. Organoids were digested by TrypLE Express and Dispase to single cells and 2000 cells were seeded in 10 μl of 4 mg/ml matrigel per well in 384-well plates (PhenoPlate 384, Revvity #6057302). After 20 min at 37°C, 15 μl feeding medium was added on top of the matrigel and cells were cultured for 3–4 days to obtain pancreatic organoids. Compounds were introduced by acoustic dispenser (LabCyte Echo 525) and 1000 PFU of virus was added in 10 µl of feeding medium per well for a final volume of 35 µl per well. Estimated multiplicity of infection based on organoid doubling rate: 1 to 5. After 72 h, 10 µl of supernatant per well was carefully withdrawn for virus titration. CTG reagent (25 µl) was added per well. After 10 min at RT luminescence was read on the Agilent Cytation 5 plate reader/imager.

The brain organoids were generated from human induced pluripotent stem cells (iPSCs) according to a published protocol with a few modifications [27]. The dense, intact, and single spheroids of ≥250 µm were transferred to an ultra-low attachment 96-well plate (CLS7007) on Day 7. On Day 9 of neural differentiation spheroids were transferred to 24-well plate (Corning, 3472). For the first week of transfer, half of the OLIG3 media was changed every third day. Later, two-thirds of the medium were changed every third day until day 30. By Day 30, the organoids were expressing neural progenitor markers.

Retinal organoids were generated from the iPSCs using modified versions of the published protocol [28, 29]. The modifications include alterations to medium composition, differentiation mediators and timing of their addition, and initial seeding density during aggregation. Additionally, orbital shaking was employed throughout the differentiation and maintenance to increase nutrient uptake. On Day 20, the retinal organoids were collected for the viral infection assay.

One hBO or hRO was placed in each well in an ultralow attachment 96-well plate, with a total of 100 µl of medium per well. Compounds (5 µM vemurafenib, 0.1 µM pleconaril, 1 µM AG7404, and 5 µM mindeudesivir) and their combinations were introduced, and the virus (100 TCID_50_) or mock were added. After 72 h, 30 µl of medium from each well was taken for virus detection by RT-qPCR.

### Drug synergy calculations

To test whether the drug combinations act synergistically, the observed responses were compared with expected combination responses. The expected responses were calculated based on the Bliss reference model using SynergyFinder version 3 to reveal whether changes in drug concentrations are associated with substantial increases in potency and efficacy [30]. Synergy scores were quantified as average excess response due to drug interactions for the most synergistic 4 × 4 × 3 dose-windows in dose–response matrix (i.e. 10% of cell survival beyond the expected additivity between single drugs represents a synergy score of 10).

### RT-qPCRs

Total RNA was isolated using Macherey-Nagel NucleoSpin^®^ RNA II kit (Bioke, Leiden, The Netherlands). cDNA was synthesized using a cDNA synthesis kit (TaKaRa Bio, Inc., Shiga, Japan). Real-time PCR reactions were performed with PowerTrack™ SYBR Green Master Mix for qPCR (Applied Biosystems, Austin, USA) on a QuantStudio™ 3 Real-Time PCR System (Thermo Fisher Scientific LifeSciences). Template control and reverse transcriptase control were included in all RT-qPCR experiments. The primers used are GAPDH_fw: GTCTCCTCTGACTTCAACAGCG, GAPDH_rv: ACCACCCTGTTGCTGTAGCCAA, EV1_fw: TCCTCCGGCCCCGTGA, and EV1_rv: RATTGTCACCATAAGCAGCCA.

### RNA sequencing

The RNA samples were reverse transcribed and amplified using the whole transcriptome amplification WTA2 kit (Sigma–Aldrich, Merck KGaA, Darmstadt, Germany). The sequencing libraries were prepared using Oxford Nanopore Technologies (ONT) SQK-RBK114.24 Rapid Barcoding Kit (ONT, Oxford, UK) and sequenced using an ONT MinION Mk1C sequencer with an R10.4.1 flow cell and MinKNOW software with Fast basecalling.

The virus genomes were assembled using the HaVoC-pipeline [31]. The sequence reads were trimmed using fastp [32], assembled to the reference genome using the BWA–MEM algorithm (https://doi.org/10.48550/arXiv.1303.3997) followed by single nucleotide variant calling using LoFreq version 2 [33] and consensus sequence calling with BCFTools [8]. The sequences are shown in Supplementary Table S4. The NGS data were deposited in the European Nucleotide Archive (ENA) at EMBL–EBI under accession number PRJEB102495 (https://www.ebi.ac.uk/ena/browser/view/ERP183887).

### Mice experiments

Three-week-old Swiss albino mice were inoculated intraperitoneally once a day (starting on day 1) for 5 days with vemurafenib (10 mg/kg), AG7404 (5 mg/kg), and pleconaril (20 mg/kg) or combinations of these molecules dissolved in 10% DMSO, 40% PEG-300, 5% Tween-80, and 45% PBS. The mice were inoculated intraperitoneally with CVB4 E2 (6 × 10^6^ TCID_50_ in 100 µl of PBS) on Day 2. The animals were sacrificed on Day 6, the pancreas was collected and frozen for determination of viral titer. Briefly, frozen pancreas was weighed, crushed using a TissueRuptor, homogenized in 0.5 ml of PBS, and then centrifuged at 2000 × *g* for 10 min at 4°C. The supernatants were harvested and the titer of infectious particles in supernatants was determined on HEp-2 cells using the endpoint dilution assay. The Spearman–Karber method was used to determine the 50% tissue culture infectious dose (TCID50) and virus titers were normalized to tissue weight. The results were expressed as log TCID50 per gram. The limit of detection of the test was 1.5 log TCID50/g.

In another experiment, pleconaril, AG7404 and mindeudesivir were dissolved in polyethylene glycol 400 (PEG 400). Final gavage volume was 10 ml kg⁻¹. Random-bred ICR neonatal mice (Charles River; <24-h-old; mixed sex) were housed with dams under SPF conditions (22°C, 12 h light/dark). Each pup received 0.02 ml of the virus suspension (20 MLD₅₀) subcutaneously. Treatments began 1 h post-infection (Day 0). CAA schedules consisted of a repeating 3-day cycle: Day 0 P → Day 1 A → Day 2 M, repeated until day 11 (total 12 days). Alternate cycles (P→M→A; M→A→P; A→P→M) and simultaneous triple dosing were tested. Double combinations and monotherapies were given daily. Doses were P 20 mg kg⁻¹; A 1 mg kg⁻¹; M 5 mg kg⁻¹ unless stated otherwise. The endpoint was survival up to 12 days post infection (dpi). Animals meeting humane endpoints (≥25% weight loss, severe paralysis) were euthanized and recorded as deaths. Group sizes varied due to funding availability and 3R ethical considerations to minimize animal use.

### Ethics approval

The study involved commercially available human cell lines, WT virus strains, which were isolated from patient samples or obtained from ATCC. All experiments with viruses were performed in BSL2 laboratories in compliance with the guidelines of the national authorities, and under appropriate ethical and safety approvals. All experiments conducted with pancreatic cancer organoids have been approved by the Helsinki University Hospital ethical board, HUS/74/2023, HUS/23/2024. Animal experiments were approved by the Bulgarian Food Safety Agency (permit No. 155/24.05.2025) and by the local Ethical Committee for Animal Experimentation (C2EA-75, Nord–Pas-de-Calais, France), as applicable to the respective studies, and were conducted in accordance with Directive 2010/63/EU.

## Results

Vemurafenib is an approved, orally available anticancer drug primarily used for treating B-type rapidly accelerated fibrosarcoma (BRAF)-mutated melanoma [34]. It was identified in our lab as a potential anti-enterovirus inhibitor through a drug repurposing effort aimed at discovering novel inhibitors against echovirus 1 (EV1) [15]. The vemurafenib’s antiviral activity, could be mediated through host-directed mechanisms such as inhibition of PI4KB, a key cellular kinase involved in enterovirus replication [35, 36].

**Figure 1.**
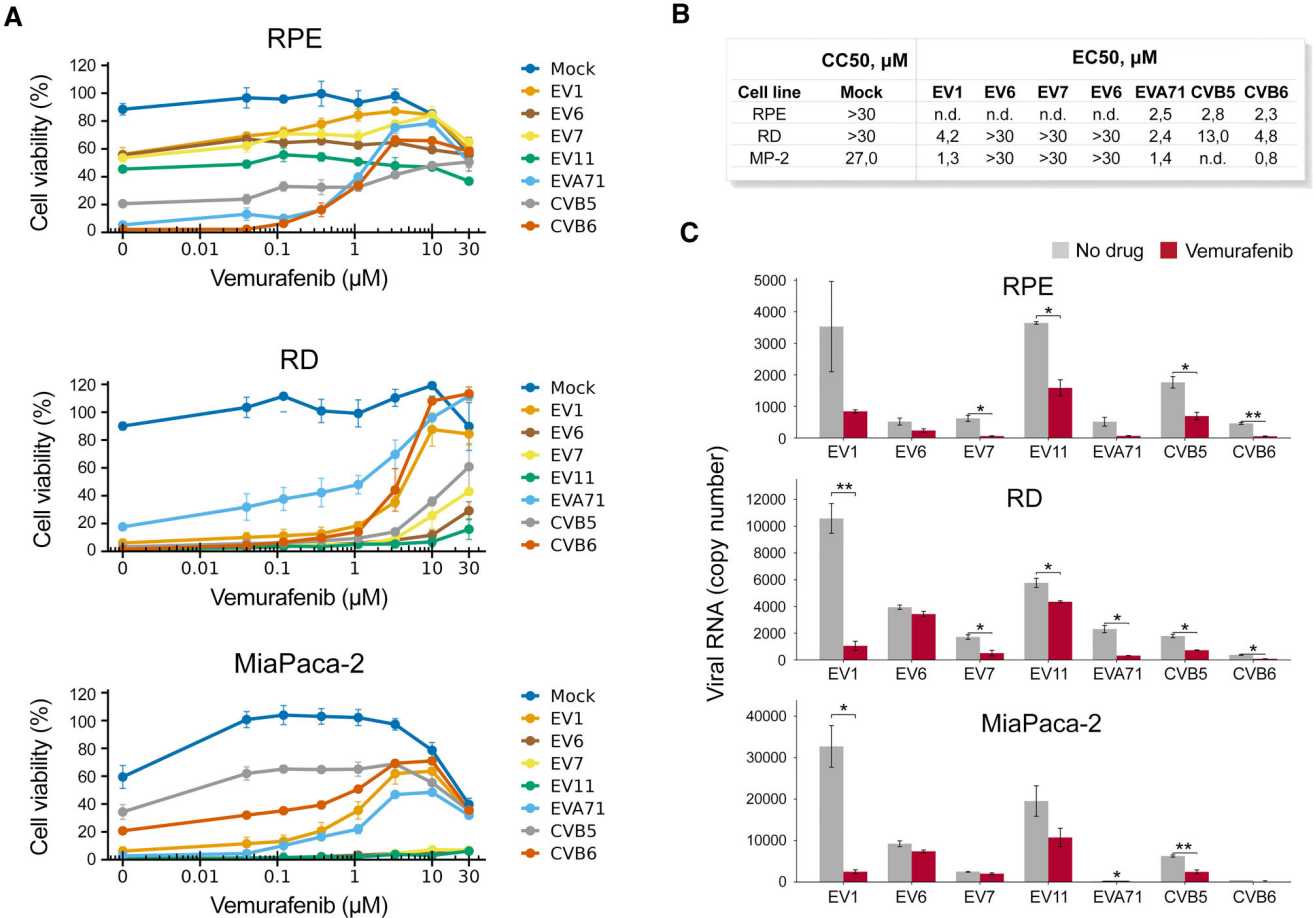
Vemurafenib inhibits replication of some enteroviruses in cell cultures. (**A**) RD, RPE, or MP2 cells were treated with increasing concentrations of vemurafenib or DMSO and infected with enteroviruses (100 TCID_50_) or mock. The viability of the cells was determined at 48 hpi with the Cell Titer Glo assay (mean ± SD, *n* = 3). (**B**) Data presented in (A) was used to calculate EC_50_ values for vemurafenib. (C) Cells were treated with 3 μM vemurafenib or DMSO, infected with viruses (moi 0.1) or mock. After 48 h, cell culture supernatants were collected, and RNA was extracted. Viral RNA was analyzed by RT-qPCR (mean ± SD, *n* = 3). Welch’s *t*-test statistics (*P < 0.05, **P < 0.01).

We tested vemurafenib against EV1, EV6, EV7, EV11, EVA71, CVB5, and CVB6 viruses in human RPE, RD, and MiaPaCa-2 (MP2) cell lines. Seven different concentrations of the compound were added to virus- or mock-infected cells. After 48 h cell viability was measured using CTG assay (Fig. 1A). Because baseline virus-induced cytopathic effect differed across virus–cell combinations (reflecting differences in entry receptors/host-factor usage and replication kinetics), the dynamic range of the viability readout and the quality of dose–response curve fitting varied, and EC50 values could therefore be determined only for virus–cell pairs with reliable fits. At noncytotoxic concentrations vemurafenib rescued RPE cells from EVA71- and CVB6-, and RD and MP2 cells from EV1-, EVA71-, and CVB6-mediated death. EC_50_ values were 1–13 μM, CC_50_ values were >27 μM (Fig. 1B). RT-qPCR revealed that 3 μM vemurafenib significantly (*P *< 0.05) reduced EV7, EVA71, and CVB6 RNA levels in the RPE cell culture medium, EVA71, CVB5, and CVB6 RNA levels in RD cell culture medium, and EVA71 and CVB6 in MP2 cell culture medium (Fig. 1C). Thus, vemurafenib inhibited replication of some but not all tested viruses *in vitro*.

**Figure 2.**
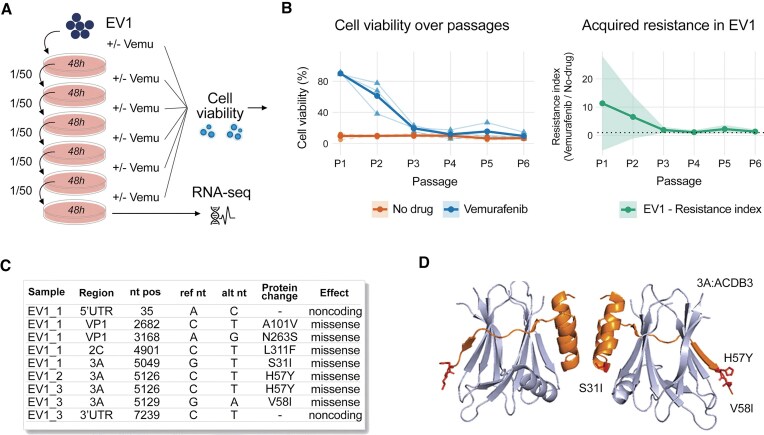
Drug-resistance test. (**A**) Schematic representation of the experiment. (**B**) EV1 (starting 100 TCID_50_) was passaged six times in RD cells in the presence of 5 μM vemurafenib or DMSO (*n* = 3) Viability of cells was determined using a CTG assay. (**C**) Cell culture supernatants were collected, and RNA was extracted and sequenced. Mutations found in EV1 genomes after 6 passages of the virus in RD cells in the presence or absence of vemurafenib were determined. (**D**) Some mutations were mapped onto structure of 3A protein (orange) in complex with host ACDB3 domain (blue) of Golgi resident protein GCP60 (PDB ID: 5LZ6).

Enteroviruses may develop resistance to drugs due to the error prone nature of 3Dpol. Therefore, we probed the development of resistance of sensitive enteroviruses to vemurafenib *in vitro*. Specifically, we passaged EV1 on RD cells in the presence or absence of 5 μM vemurafenib in triplicate, as illustrated in Fig. 2A. Vemurafenib demonstrated protection of RD cells from EV1-mediated death only for two passages (Fig. 2B). After six passages, we sequenced the viral RNA and identified several mutations potentially linked to drug resistance. In one biological replicate, missense mutations emerged in VP1 (A101V, N263S), 2C (L311F), and 3A (S31I), in othe rtwo replicates, a missense mutation emerged in 3A (H57Y), and in the third replicate additional mutation was detected in 3A (V58I) (Fig. 2C). Notably, no missense mutations emerged in the absence of vemurafenib. Similarly, we were able to obtain vemurafenib-resistant CVB5 strain carrying K132Q in VP1 and V58I in 3A (Supplementary Table S5). Although multiple mutations emerged during passaging, the recurrence of 3A and VP1 across two enteroviruses under vemurafenib selection supports its potential role in resistance.

**Figure 3.**
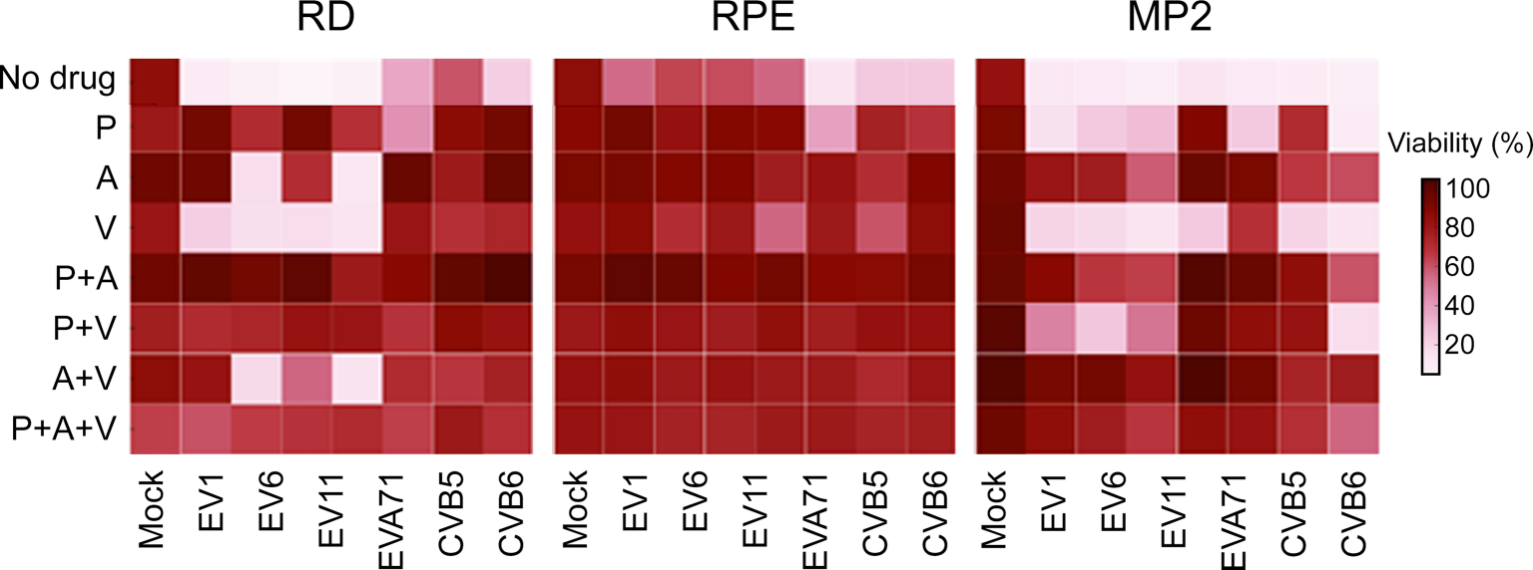
Anti-enteroviral effect of pleconaril (P), AG7404 (A), vemurafenib **(V)** and their combinations in RD, RPE, and MP2 cell cultures. Cells were treated with 0.2 μM P, 1 μM A, 5 μM V or their combinations, and infected with enteroviruses (100 TCID_50_). After 72 h, cell viability was determined using a CTG assay (mean, *n* = 3).

Naturally occurring vemurafenib-resistant viruses and the emergence of vemurafenib resistance in sensitive strains underscored the need for combination therapy. We therefore evaluated combinations, pairing 5 µM vemurafenib with 0.2 µM pleconaril (the VP1 inhibitor) and 1 µM AG7404 (3C protease inhibitor). After 72 h, viability of virus- and mock-infected RD, RPE, and MP2 cells was measured using CTG assay. The P + A + V combination suppressed replication of all seven tested enteroviruses (Fig. 3). By contrast, treatment with single agents was not efficient against all tested strains. The viral coverage was similar to combinations of pleconaril, AG7404, and mindeudesivir (M), an inhibitor of viral RNA synthesis, which we developed previously [17], as well as other triple combinations containing P, A, V, and M (Supplementary Fig. S1). These results support a combination approach which targets different stages of virus replication including virus entry, viral protein processing, and viral RNA replication.

**Figure 4.**
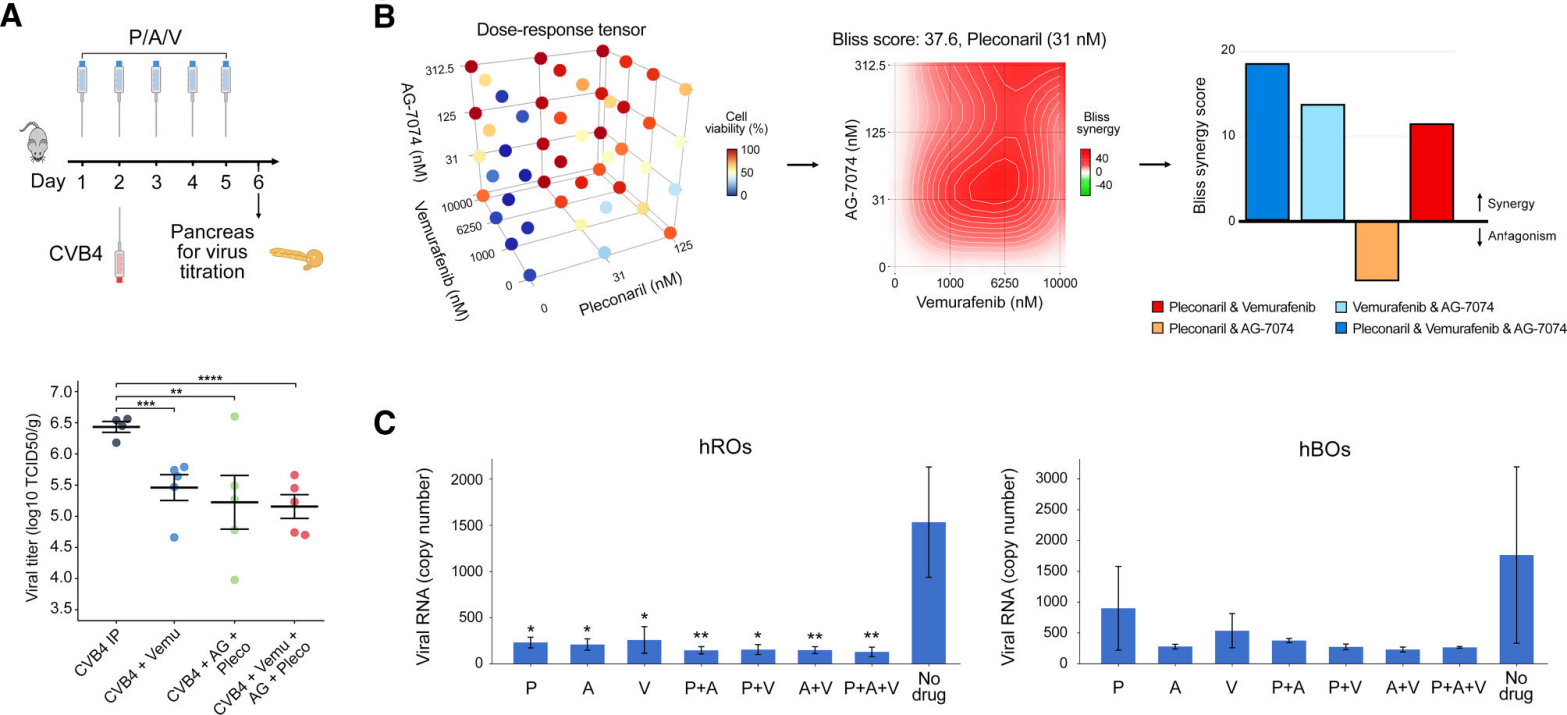
Antiviral efficacy of combination of pleconaril (P), AG7404 (A), and vemurafenib (V) in mice and human organoids. (**A**) 10 mg/kg V, 5 mg/kg A, and 20 mg/kg P were administered IP 5 consecutive days to 3-week-old Swiss albino mice. CVB4 (6 × 10 ^6^ TCID50 in 100 µl) was inoculated on Day 2 by IP route. Pancreas was recovered on Day 6. The organs were weighed and ground in 500 µl of PBS. After centrifugation, the supernatant of the crushed organs was collected for viral titration. Titers were normalized to tissue weight. Results are expressed as log_10_ TCID_50_ per gram. (**B**) Pancreatic organoids (hPOs) were treated with 0.1 µM P, 1 µM A, 5 µM V or their combinations, and infected with the EV1 (100 TCID_50_). After 72 h, CTG reagent was added, luminescence was measured. The 4 × 4 × 3 interaction landscapes were obtained for the combination, and Bliss synergy scores were calculated. (**C**) Retinal (hROs) and brain (hBOs) organoids were treated with 0.2 μM P, 1 μM A, 5 μM V or their combinations, and infected with EV11 (100 TCID50). After 72 h, culture supernatants were collected, and RNA was extracted. Viral RNA was analyzed by RT-qPCR (mean ± SD, *n* = 3); *P < 0.1, **P < 0.05, ***P < 0.01, ****P < 0.001.

Efficacy of the P + A + V combination was further validated in a mouse model. 20 mg/kg pleconaril, 5 mg/kg AG7404, and 10 mg/kg vemurafenib were administered intraperitoneally for five consecutive days (D1–D5) to 3-week-old Swiss albino mice. CVB4 (6 × 10^6^ TCID50 in 100 µl) was inoculated on D2 by the IP route. The pancreas was recovered on Day 6, viruses were extracted and titrated. Titers were normalized to tissue weight. The P + A + V combination significantly reduced virus titers in the pancreas of infected animals (Fig. 4A). By contrast, the P + A + M combination was not effective in mice when given simultaneously, perhaps due to drug toxicity or drug–drug interaction (Supplementary Table S6) [39–41].

Efficacy of the P + A + V combination was further validated in human pancreatic (hPOs), retinal (hROs), and brain (hBOs) organoid systems, which are a necessary step before clinical trials. First, we generated hPOs from pancreatic cancer cells [25]. hPOs were treated with 0.1 µM P, 1 µM A, 5 µM V, or their combinations, and infected with EV1 (100 TCID50). After 72 h, CTG reagent was added, and luminescence was measured. The 4 × 4 × 3 interaction landscape was obtained, and Bliss synergy scores were calculated (Fig. 4B). The Bliss scores indicated that the triple combination was synergistic, i.e. reduced concentrations of the small molecules were needed to achieve antiviral effect compared to double-drug combinations. Second, we generated eye and brain organoids from iPSCs derived from healthy donors. hROs and hBOs were treated with 0.2 µM P, 1 µM A, 5 µM V, or their combinations, and infected with EV11 (100 TCID_50_). After 72 h, culture supernatants were collected, and RNA was extracted. Viral and host RNA was analyzed by RT-qPCR. RT-qPCR showed that the combination attenuated viral replication while preserving host RNA levels (Fig. 4C and Supplementary Fig. S2). Thus, we identified a combination of orally available, safe-in-human agents that inhibits a broad range of enterovirus infections in different model systems.

## Discussion

The Enterovirus genus within the Picornaviridae family includes 14 species. All enteroviruses share a similar organization but differ in their genetic determinants and in their dependence on specific host factors. These differences likely translate into variable sensitivity across virus–cell combinations to different antivirals such as AG7404 and pleconaril. Moreover, the emergence of drug resistance during replication in initially sensitive strains underscores that virus-specific adaptation to the relevant host pathway(s) can shape the antiviral response.

Here, we observed both (i) naturally occurring vemurafenib-resistant viruses and (ii) the emergence of resistance during passaging in initially sensitive strains, which together underscore that virus-specific adaptation to the relevant virus-host interactions can shape the antiviral response. In particular, mutations in 3A suggests that they could destroy its interaction with the host ACBD3 domain of the Golgi-resident protein GCP60 and associated proteins, such as phosphatidylinositol 4-kinase IIIβ (PI4KB) involved in the assembly of replication organelles [35, 37, 38].

The rapid development of resistance to monotherapies highlights the need for a combination approach. Indeed, a drug cocktail containing safe-in-human, orally available vemurafenib, AG7404 and pleconaril inhibited all seven tested enteroviruses at nontoxic concentrations in human retinal RPE, rhabdomyosarcoma RD, and pancreatic MiaPaca-2 cells. In infected mice, the triple regimen reduced viral titers in the pancreas. The combination was also effective in human pancreatic, retinal, and brain organoids. Thus, this combination consistently exhibits antiviral effects across a range of enteroviruses, emphasizing its potential as a broad-spectrum antiviral cocktail.

**Figure 5.**
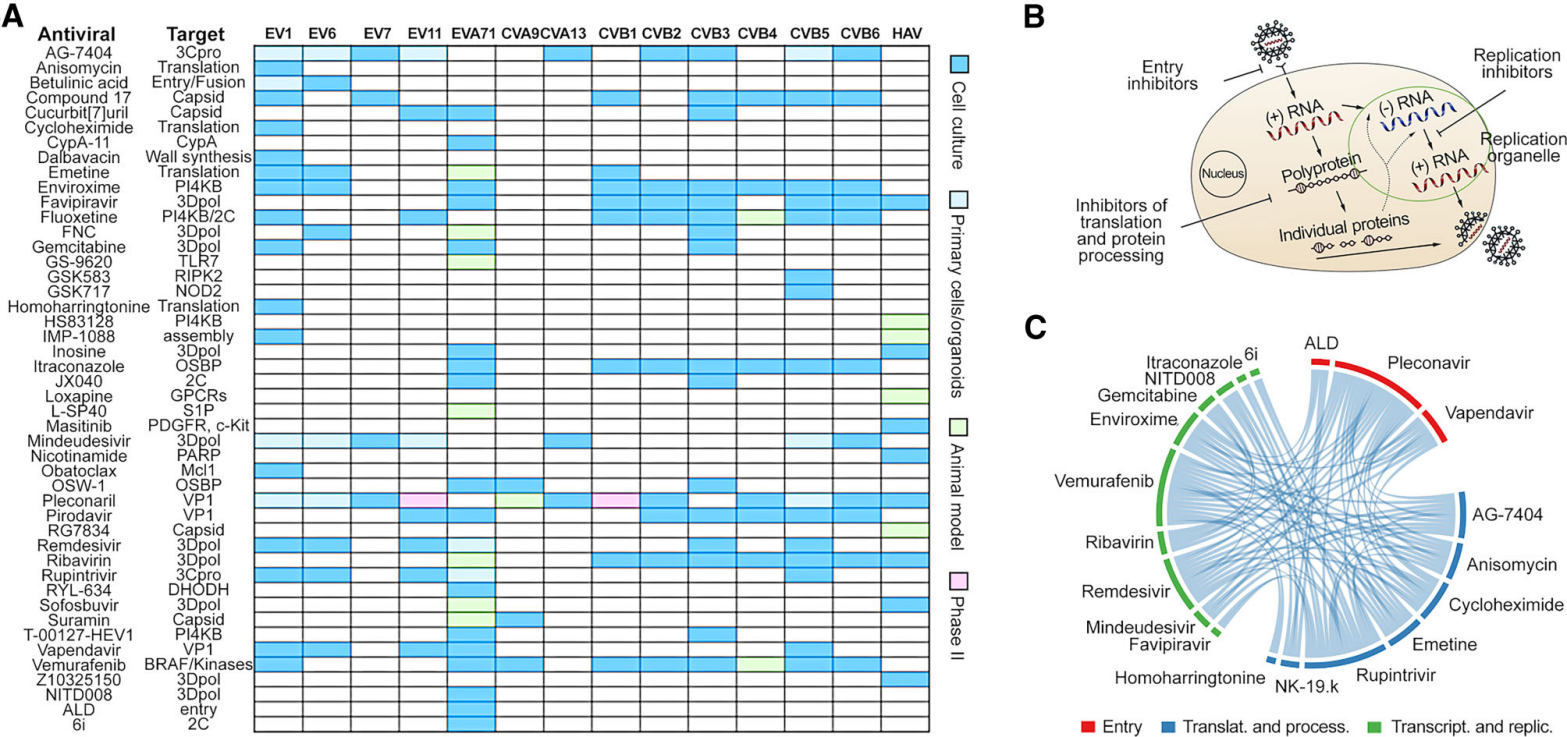
Targeting multiple stages of the enterovirus life cycle is a promising strategy for broadly active therapeutic combinations. (**A**) Representative anti-enteroviral inhibitors. (**B**) These agents primarily block viral entry, translation and polyprotein processing, or RNA replication within replication organelles. (**C**) Several combinations show synergistic or additive effects across model systems.

Our findings also support multistage targeting of the enterovirus life cycle as a path toward broadly active therapeutics. Supplementary Table S7 and Fig. 5A summarizes known anti-enteroviral inhibitors [15, 36–38, 42–59]. Interestingly, these agents mainly target virus entry, viral RNA translation and polyprotein processing as well as viral RNA replication in replication organelles (Supplementary Table S8 and Fig. 5B). Figure 5C shows that some of these agents were used in combinations and were shown to be synergistic or additive *in vitro* or *in vivo* [19, 60–63]. Further development of such combinations with high resistance barriers, both *in vitro* and *in vivo*, as well as in clinical trials, could provide a potential treatment solution for diseases associated with enteroviruses [64].

## Supplementary Material

ugaf046_Supplemental_File

## Data Availability

All data generated or analyzed during this study are included in this published article and its supplementary information files.
