# Supplementary material for: Combination of vemurafenib, pleconaril, and AG7404 attenuates enterovirus replication in vitro and in vivo
Source: NAR Mol Med. 2025 Dec 23;3(1):ugaf046. doi: 10.1093/narmme/ugaf046 (PMC12783043; doi:10.1093/narmme/ugaf046)
Supplement: ugaf046_Supplemental_File [file ugaf046_supplemental_file.docx]

Supplementary data

Table S1. Chemical compounds used as antivirals in this study.

| **Compound** | **Oral** | **Supplier** | **Catalog N.** | **Cas N.** |
| --- | --- | --- | --- | --- |
| Pleconaril | Yes | Cayman Chemical | CAYM28461 | 153168-05-9 |
| AG7404 | Yes | MEDKOO | 530656 | 343565-99-1 |
| Mindeudesivir | Yes | MedChemExpress | HY-145119AS | 2779498-79-0 |
| Vemurafenib | Yes | MedChemExpress | HY-12057 | [**918504-65-1**](https://www.medchemexpress.com/cas/918504-65-1.html) |

Table S2. Virus strains used in this study.

| **Virus name*** | **ICTV name**** | **ICTV species *** | **Reference** |
| --- | --- | --- | --- |
| EVA71 | EV-A71 | *Enterovirus alphacoxsackie* | ATCC VR-1775 |
| EV1 | E1 | *Enterovirus betacoxsackie* | ATCC VR-1808 |
| EV6 | E6 | *Enterovirus betacoxsackie* | [64] |
| EV7 | E7 | *Enterovirus betacoxsackie* | [65] |
| EV11 | E11 | *Enterovirus betacoxsackie* | [18] |
| CVB1 | CVB1 | *Enterovirus betacoxsackie* | ATCC VR-28 |
| CVB5 | CVB5 | *Enterovirus betacoxsackie* | [18] |
| CVB6 | CVB6 | *Enterovirus betacoxsackie* | ATCC VR-155 |

* - Virus names are not official ICTV designations.

** - ICTV names are from <https://ictv.global/report/chapter/picornaviridae/picornaviridae/enterovirus>

Table S3. Cells used in this study.

| **Cells** | **Reference** |
| --- | --- |
| Human adenocarcinoma alveolar basal epithelial A549 cells | ATCC CCL-185 |
| Human pancreatic cancer MIA PaCa-2 cells | ATCC CRM-CRL-1420 |
| Human rhabdomyosarcoma RD cells | ATCC CCL-136 |
| Human immortalized retinal pigment epithelium RPE cells | CRL-4000 |
| Human iPS generated from ATCC dermal fibroblast | ATCC ACS-1011 |

Table S4. EV1 sequencing results.

| >1E1_Barcode01_EV1_consensus | TTTGTGCGCCTGTTTTATACTCCCCCCCCTAAGGAAACTTTAGAAGCAAAGCAATTGTGATCAATAGTGGGTATGGCACACCAGTCATATCTTGATCAAGCACTTCTGTTCCCCCGGACTTAGTACCAATAGACTGCTCAAGCGGTTGAAGGGGAAAACGTTCGTTATCCGGCCAACTACTTCGAGAAACCTAGTAGCACCATGAAAGTTGCGGAGTGTTTCGCTCAGCACTTCCCCCGTGTAGATCAGGCTGATGAGTCACCGTATTCCCCACGGGCGACCGTGACGGTGGCTGCGTTGGCGGCCTGCCCATGGGGTAACCCATGGGACGCTCTAAAACAGACACGGTGCGAAGAGTCTATTGAGCTAGTTGGTAGTCCTCCGGCCCCTGAATGCGGCCAATCCTAACTGCGGAGCACATACTCCCAATCCAGGGAGCAGTGTGTCGTAATGGGTAACTCTGCAGCGGAACCGACTACTTTGGGTGTCCGTGTTTCCTTTTATTCTCACATTGACTGCTTATGGTGACAATTGAAAGATTGTTACCATATAGCTATTGGATTGGTCATCCGGTGAGCAATAGAGCTATTGTTTATCAATTTGTTGGATTTGTACCACTCAACTTTTCTGTTTTGAGAACACTCAACTACATCTTACTGCTAAACACATCAAAATGGGAGCACAGGTATCAACACAGAAGACCGGGGCGCACGAGACTAGCTTGAGCGCTACTGGTAACTCCATAATACACTACACGAATATTAATTATTACAAAGATGCAGCCTCTAACTCTGCCAATAGACAAGATTTCACCCAAGACCCTGGTAAGTTTACTGAACCAGTGAAAGATGTCATGATAAAAACCCTGCCAGCGCTGAATTCTCCAACGGTTGAAGAGTGCGGGTACAGTGACAGGGTCAGGTCAATCACACTTGGGAACTCCACTATTACAACTCAAGAGTGTGCCAATGTGGTGGTGGGGTACGGTGAATGGCCTGAGTATCTGAGTGATAACGAGGCAACTGCTGAGGACCAACCAACGCAGCCGGACGTGGCCACTTGCCGTTTTTACACCCTAGACTCAGTCCAATGGGAGAATGGGTCACCAGGTTGGTGGTGGAAGTTTCCCGACGCTCTAAGGGATATGGGATTATTTGGCCAAAATATGTACTACCATTACTTAGGCAGAGCCGGGTATACCATCCACGTACAATGCAATGCTTCCAAGTTTCATCAAGGCTGTATCCTGGTAGTGTGTGTCCCTGAGGCGGAGATGGGAAGTGCCCAAACCTCAGGGGTGGTCAACTACGAACACATTAGTAAGGGTGAGATCGCATCAAGGTTCACTACCACGACAACAGCAGAAGACCATGGCGTGCAGGCCGCGGTATGGAATGCTGGTATGGGCGTTGGAGTTGGGAACTTGACGATCTTCCCGCACCAATGGATCAACCTTCGCACCAACAACAGCGCCACAATTGTTATGCCATACGTAAATAGTGTACCAATGGACAATATGTATAGACATCACAACTTTACACTAATGATAATACCCTTTGTGCCTCTGGATTTCAGCGCGGGTGCATCCACATACGTGCCCATAACGGTGACAGTGGCCCCCATGTGTGCCGAGTACAATGGACTACGACTAGCTGGACACCAAGGACTACCGACCATGAACACCCCTGGCAGCAACCAATTTCTTACATCGGACGATTTCCAATCCCCGTCAGCGATGCCTCAATTTGATGTAACTCCAGAAATGCACATCCCTGGTGAGGTGCGCAACCTCATGGAAATTGCCGAAGTTGATTCTGTAATGCCAATTAACAATGATAGCGCCGCAAAAGTTTCATCCATGGAGGCTTATAGAGTCGAATTGAGCACCAACACTAATGCCGGGACTCAAGTGTTTGGCTTTCAACTGAACCCCGGAGCGGAATCAGTAATGAACCGCACATTAATGGGTGAAATCCTAAATTACTACGCACACTGGTCAGGAAGCATAAAGATAACATTCGTGTTCTGTGGTTCTGCCATGACCACTGGCAAGTTTCTGCTGTCTTACGCCCCACCAGGTGCAGGTGCGCCAAAAACTCGCAAGGATGCCATGTTAGGCACTCATGTGGTGTGGGATGTTGGGCTCCAATCCAGCTGCGTGTTATGCATCCCCTGGATTAGTCAAACCCATTACAGATTTGTGGAAAAGGATCCATACACCAATGCCGGGTTTGTGACATGTTGGTATCAGACCAGTGTAGTGTCCCCAGCGAGCAACCAGCCAAAGTGTTATATGATGTGCATGGTTTCTGCGTGTAATGACTTCTCAGTTCGCATGTTGAGAGATACCAAGTTCATTGAGCAAACATCTTTTTACCAAGGTGATGTGCAGAATGCTGTCGAAGGGGCTATGGTCAGGGTGGCAGATACAGTGCAAACTTCAGCCACAAACTCAGAGAGGGTGCCTAACTTGACAGCAGTAGAAACTGGTCACACTTCGCAGGTAGTACCTGGTGATACCATGCAGACTAGACATGTGATCAACAATCACGTGAGGTCAGAATCTACAATTGAGAACTTCCTTGCCAGATCAGCGTGTGTTTTCTTCCTAGAGTACAAGACAGGGACCAAAGAGGATTCCAATAGCTTCAACAATTGGGTGATTACAACCAGGCGAGTGGCTCAACTACGTAGAAAACTGGAAATGTTTACTTACCTACGGTTTGACATGGAAATCACCGTGGTCATTACAAGCTCGCAAGATCAGTCTACATCACAAAACCAGAATGCACCAGTGCTAACACACCAGATAATGTATGTACCACCAGGGGGACCCATACCCATAAGCGTGGATGATTACAGCTGGCAAACATCCACCAACCCCAGTATCTTTTGGACCGAAGGGAACGCTCCGGCACGCATGTCAATTCCATTTATTAGCATAGGCAATGCGTATAGTAATTTCTACGATGGGTGGTCTCACTTCTCCCAGAATGGCGTGTATGGCTTCACTACTCTGAACAACATGGGTCAATTGTTCTTCCGGCACGTAAACAAGCCCAACCCAGCCGCTATTACAAGTGTGGCGCGCATTTACTTCAAACCGAAACATGTACGCGCTTGGGTGCCTAGACCACCGCGCTTGTGTCCATACATCAATAGCACGAATGTCAACTTTGAACCCAAGCCAGTGACTGAAGTACGTACCAACATAATAACAACGGGTGCCTTTGGGCAGCAATCTGGCGCAGTGTACGTGGGCAACTACAGAGTGGTCAATAGGCACTTGGCGACTCACATTGATTGGCAAAACTGTGTGTGGGAGGACTATAACAGGGATCTACTGGTCAGCACAACTACAGCTCATGGGTGCGACACCATAGCTAGGTGCCAGTGCACGACAGGGGTGTACTTCTGCCTGAGCAGAAACAAACACTACCCAGTGTCATTTGAAGGTCCAGGATTGGTTGAGGTTCAAGAGAGTGAGTATTACCCAAGAAGGTACCAATCCCACGTGCTTCTTGCAGCCGGATTTTCTGAACCTGGAGATTGTGGTGGTATCTTGAGGTGTGAGCATGGTGTTATTGGTATAGTGACCATGGGAGGTGAAGGTGTCGTTGGTTTCGCCGATGTGCGAGACCTTCTATGGCTAGAGGATGACGCCATGGAGCAGGGAGTCAAGGACTACGTGGAACAGCTCGGCAACGCCTTTGGTTCAGGTTTCACCAATCAGATTTGTGAGCAGGTCAATCTCCTGAAAGAGTCCTTGGTAGGTCAAGACTCCATCTTGGAAAAGTCTTTAAAAGCACTAGTAAAAATCATATCAGCATTAGTGATCGTGGTAAGGAACCACGACGACTTGATCACAGTGACTGCTACACTAGCCCTCATTGGCTGCACCTCTTCACCATGGCGATGGCTCAAGCAGAAGGTATCACAATATTATGGAATACCCATGGCCGAGCGTCAGAACAATGGATGGCTCAAGAAATTCACTGAGATGACTAACGCCTGCAAAGGCATGGAGTGGATTGCCATTAAAATTCAGAAATTTATTGAATGGCTGAAAGTTAAGATTCTACCTGAAGTAAAAGAAAAACATGAATTTCTCAATAGATTAAAACAGCTGCCACTTCTTGAAAGTCAGATTGCTACCATAGAACAGAGCGCACCATCACAAGGTGACCAAGAACAGCTCTTCTCCAATGTGCAGTATTTTGCCCACTATTGCAGAAAGTACGCACCTCTGTATGCCGCCGAAGCAAAAAGAGTGTTCTCGTTGGAGAAAAAGATGAGCAACTACATACAGTTCAAGTCCAAATGCCGTATTGAGCCTGTATGTTTACTTCTCCATGGCAGCCCAGGAGCGGGGAAATCCGTGGCTACAAACCTAATTGGTAGATCCCTCGCGGAGAAACTTAACAGCTCTGTGTACTCGTTACCACCAGACCCAGATCATTTTGATGGATACAAACAACAAGCCGTAGTGATCATGGATGACCTGTGCCAGAATCCAGATGGGAAGGATGTGTCACTATTCTGTCAAATGGTATCCAGCGTGGACTTCGTACCACCCATGGCAGCTCTGGAGGAGAAAGGGATTCTTTTCACGTCCCCGTTTGTGCTAGCATCAACCAATGCGGGGTCTATCAATGCACCCACTGTGTCTGACAGCAGGGCACTTGCCAGAAGGTTCCACTTTGATATGAACATCGAGGTGATCTCCATGTATAGCCAGAATGGGAAGATTAACATGCCCATGTCTGTCAAAACATGTGATGAGGATTGCTGCCCGGTCAATTTCAAGAAATGCTGCCCGCTGGTGTGTGGTAAGGCCATTCAATTTATTGACAGAAAGACCCAAGTTAGGTATTCACTGGACATGTTGGTCACCGAGATGCTCAGGGAGTACAACCACAGACACAGCGTGGGTGCCACCCTCGAGGCTTTGTTCCAAGGGCCACCGGTCTACAGGGAGATTAAGATCAGTGTCGCTCCAGAAACACCCCCTCCACCAGCAATCGCTGACCTGCTAAAATCAGTAGACAGTGAGGCAGTAAGGGAGTACTGCAAGGAAAAAGGCTGGCTTGTGCCGGAAATTAGCTCCACCCTACAGATTGAGAAGCACGTCAGTAGAGCATTTATCTGCCTACAGGCTCTGACTACATTTGTCTCAGTAGCTGGCATAATCTACATTATCTACAAATTGTTTGCCGGTTTTCAGGGCGCGTATACGGGGATGCCAAATCAGAAACCCAAGGTGCCCACTCTGAGACAGGCTAAGGTGCAGGGCCCGGCATTCGAGTTCGCCGTGGCGATGATGAAGAGAAACGCCAGCACAGTGAAAACAGAATATGGTGAGTTCACCATGCTCGGCATCTATGACAGATGGGCAGTGTTACCACGCCACGCCAAGCCCGGACCGACCATCTTAATGAATGATCAGGAGGTCGGTGTGCTAGATGCCAAAGAATTGGTTGACAAAGATGGGACAAATCTGGAGTTGACTCTCCTAAAGCTCAATCGCAATGAGAAGTTTAGGGATATCAGAGGGTTTCTGGCAAGAGAAGAAGCTGAGGTGAATGAGGCTGTTTTGGCAATAAACACAAGCAAGTTCCCCAACATGTACATACCCGTAGGTCAAGTCACCGACTACGGTTTTCTGAACTTGGGAGGAACGCCCACAAAGAGGATGCTCATGTACAATTTCCCAACTAGAGCAGGCCAATGTGGCGGTGTCCTCATGTCAACAGGGAAGGTTCTAGGAATACATGTAGGCGGAAATGGACACCAAGGATTCTCTGCTGCCCTCCTTAGACATTACTTCAATGAGGAACAAGGTGAGATAGAATTCATTGAGAGCTCAAAGGACGCAGGCTTCCCTGTGATCAACACCCCCAGCAAAACCAAGCTGGAACCAAGCGTGTTTCACCAGGTGTTTGAGGGCAACAAAGAGCCGGCAGTGCTTAGAAATGGGGATCCACGACTCAAGGTCAACTTTGAGGAGGCAATCTTCTCCAAGTACATTGGCAATGTTAACACCCACGTGGACGAATACATGCAAGAGGCCGTGGACCATTATGCAGGGCAGCTAGCTACACTGGACATCAGCACAGAGCCCATGAAACTGGAGGATGCCGTGTATGGTACAGAGGGGCTGGAAGCACTAGACCTAACCACCAGTGCAGGCTATCCGTACGTGGCCCTAGGTATCAAGAAAAGAGACATTCTCTCTAAGAAGACCAAAGACCTTACCAAGTTGAAGGAATGCATGGACAAGTATGGCCTAAACTTACCAATGGTAACTTACGTCAAAGATGAATTAAGATCTGCCGAGAAGGTAGCCAAGGGAAAGTCCAGACTTATTGAGGCCTCCAGTCTCAATGACTCAGTAGCAATGAGGCAAACATTTGGAAACCTGTACAAAACCTTTCATCTCAATCCGGGCATTGTCACGGGCAGTGCTGTTGGGTGTGACCCAGATGTATTTTGGAGTAAGATCCCTGTCATGCTTGATGGACATCTCATAGCTTTTGACTATTCAGGTTATGACGCCAGTCTCAGCCCGGTGTGGTTTGCATGTCTGAAACTCCTCCTAGAGAAACTAGGGTATACGAATAAGGAAACAAACTACATAGATTACCTCTGCAACTCTCACCACTTATATAGGGACAAGCACTACTTTGTGAGAGGCGGTATGCCATCAGGATGTTCGGGCACTAGCATATTTAATTCCATGATTAACAACATTATAATCAGGACTCTCATGCTGAAAGTTTATAAAGGCATTGATTTGGACCAATTCAGAATGATCGCTTATGGGGATGATGTGATTGCCTCCTACCCGTGGCCCATCGATGCGTCACTGTTAGCTGAAGCAGGAAAAGATTATGGATTGATCATGACCCCAGCAGACAAAGGTGAGTGCTTTAATGAGGTAACCTGGACAAATGTGACCTTTTTGAAAAGGTACTTCAGAGCAGATGAACAGTACCCATTCCTGGTCCATCCTGTTATGCCAATGAAGGACATACATGAGTCCATTAGATGGACTAAAGACCCCAAAAACACACAGGATCACGTGCGCTCGCTGTGCCTATTGGCTTGGCACAACGGGGAGCACGAATATGAGGAGTTTATTCGCAAGATCAGAAGCGTGCCCGTCGGGCGCTGCTTGACCCTTCCTGCATTTTCGACACTGCGTAGGAAGTGGTTGGACTCCTTCTAAAATTAGAGCACAATTAGTAGATTACAATTGGCTTAACCCTACCGCATGAACCGAACTTGATAAAAGTGCGGTAGGGGTAAATTCTCCGCATTCGGTGCGG |
| --- | --- |
| >1E1V_Barcode02_EV1_consensus | TTTGTGCGCCTGTTTTATACTCCCCCCCCTAAGGAAACTTTAGAAGCAAAGCAATTGTGATCAATAGTGGGTATGGCACACCAGTCATATCTTGATCAAGCACTTCTGTTCCCCCGGACTTAGTACCAATAGACTGCTCAAGCGGTTGAAGGGGAAAACGTTCGTTATCCGGCCAACTACTTCGAGAAACCTAGTAGCACCATGAAAGTTGCGGAGTGTTTCGCTCAGCACTTCCCCCGTGTAGATCAGGCTGATGAGTCACCGTATTCCCCACGGGCGACCGTGACGGTGGCTGCGTTGGCGGCCTGCCCATGGGGTAACCCATGGGACGCTCTAAAACAGACACGGTGCGAAGAGTCTATTGAGCTAGTTGGTAGTCCTCCGGCCCCTGAATGCGGCCAATCCTAACTGCGGAGCACATACTCCCAATCCAGGGAGCAGTGTGTCGTAATGGGTAACTCTGCAGCGGAACCGACTACTTTGGGTGTCCGTGTTTCCTTTTATTCTCACATTGACTGCTTATGGTGACAATTGAAAGATTGTTACCATATAGCTATTGGATTGGTCATCCGGTGAGCAATAGAGCTATTGTTTATCAATTTGTTGGATTTGTACCACTCAACTTTTCTGTTTTGAGAACACTCAACTACATCTTACTGCTAAACACATCAAAATGGGAGCACAGGTATCAACACAGAAGACCGGGGCGCACGAGACTAGCTTGAGCGCTACTGGTAACTCCATAATACACTACACGAATATTAATTATTACAAAGATGCAGCCTCTAACTCTGCCAATAGACAAGATTTCACCCAAGACCCTGGTAAGTTTACTGAACCAGTGAAAGATGTCATGATAAAAACCCTGCCAGCGCTGAATTCTCCAACGGTTGAAGAGTGCGGGTACAGTGACAGGGTCAGGTCAATCACACTTGGGAACTCCACTATTACAACTCAAGAGTGTGCCAATGTGGTGGTGGGGTACGGTGAATGGCCTGAGTATCTGAGTGATAACGAGGCAACTGCTGAGGACCAACCAACGCAGCCGGATGTGGCCACTTGCCGTTTTTACACCCTAGACTCAGTCCAATGGGAGAATGGGTCACCAGGTTGGTGGTGGAAGTTTCCCGACGCTCTAAGGGATATGGGATTATTTGGCCAAAATATGTACTACCATTACTTAGGCAGAGCCGGGTATACCATCCACGTACAATGCAATGCTTCCAAGTTTCATCAAGGCTGTATCCTGGTAGTGTGTGTCCCTGAGGCGGAGATGGGAAGTGCCCAAACCTCAGGGGTGGTCAACTACGAACACATTAGTAAGGGTGAGATCGCATCAAGGTTCACTACCACGACAACAGCAGAAGACCATGGCGTGCAGGCCGCGGTATGGAATGCTGGTATGGGCGTTGGAGTTGGGAACTTGACGATCTTCCCGCACCAATGGATCAACCTTCGCACCAACAACAGCGCCACAATTGTTATGCCATACGTAAATAGTGTACCAATGGACAATATGTATAGACATCACAACTTTACACTAATGATAATACCCTTTGTGCCTCTGGATTTCAGCGCGGGTGCATCCACATACGTGCCCATAACGGTGACAGTGGCCCCCATGTGTGCCGAGTACAATGGACTACGACTAGCTGGACACCAAGGACTACCGACCATGAACACCCCTGGCAGCAACCAATTTCTTACATCGGACGATTTCCAATCCCCGTCAGCGATGCCTCAATTTGATGTAACTCCAGAAATGCACATCCCTGGTGAGGTGCGCAACCTCATGGAAATTGCCGAAGTTGATTCTGTAATGCCAATTAACAATGATAGCGCCGCAAAAGTTTCATCCATGGAGGCTTATAGAGTCGAATTGAGCACCAACACTAATGCCGGGACTCAAGTGTTTGGCTTTCAACTGAACCCCGGAGCGGAATCAGTAATGAACCGCACATTAATGGGCGAAATCCTAAATTACTACGCACACTGGTCAGGAAGCATAAAGATAACATTCGTGTTCTGTGGTTCTGCCATGACCACTGGCAAGTTTCTGCTGTCTTACGCCCCACCAGGTGCAGGTGCGCCAAAAACTCGCAAGGATGCCATGTTAGGCACTCATGTGGTGTGGGATGTTGGGCTCCAATCCAGCTGCGTGTTATGCATCCCCTGGATTAGTCAAACCCATTACAGATTTGTGGAAAAGGATCCATACACCAATGCCGGGTTTGTGACATGTTGGTATCAGACCAGTGTAGTGTCCCCAGCGAGCAACCAGCCAAAGTGTTATATGATGTGCATGGTTTCTGCGTGTAATGACTTCTCAGTTCGCATGTTGAGAGATACCAAGTTCATTGAGCAAACATCTTTTTACCAAGGTGATGTGCAGAATGCTGTCGAAGGGGCTATGGTCAGGGTGGCAGATACAGTGCAAACTTCAGCCACAAACTCAGAGAGGGTGCCTAACTTGACAGCAGTAGAAACTGGTCACACTTCGCAGGTAGTACCTGGTGATACCATGCAGACTAGACATGTGATCAACAATCACGTGAGGTCAGAATCTACAATTGAGAACTTCCTTGCCAGATCAGCGTGTGTTTTCTTCCTAGAGTACAAGACAGGGACCAAAGAGGATTCCAATAGCTTCAACAATTGGGTGATTACAACCAGGCGAGTGGTTCAACTACGTAGAAAACTGGAAATGTTTACTTACCTACGGTTTGACATGGAAATCACCGTGGTCATTACAAGCTCGCAAGATCAGTCTACATCACAAAACCAGAATGCACCAGTGCTAACACACCAGATAATGTATGTACCACCAGGGGGACCCATACCCATAAGCGTGGATGATTACAGCTGGCAAACATCCACCAACCCCAGTATCTTTTGGACCGAAGGGAACGCTCCGGCACGCATGTCAATTCCATTTATTAGCATAGGCAATGCGTATAGTAATTTCTACGATGGGTGGTCTCACTTCTCCCAGAATGGCGTGTATGGCTTCACTACTCTGAACAACATGGGTCAATTGTTCTTCCGGCACGTAAACAAGCCCAACCCAGCCGCTATTACAAGTGTGGCGCGCATTTACTTCAAACCGAAACATGTACGCGCTTGGGTGCCTAGACCACCGCGCTTGTGTCCATACATCAATAGCACGAGTGTCAACTTTGAACCCAAGCCAGTGACTGAAGTACGTACCAACATAATAACAACGGGTGCCTTTGGGCAGCAATCTGGCGCAGTGTACGTGGGCAACTACAGAGTGGTCAATAGGCACTTGGCGACTCACATTGATTGGCAAAACTGTGTGTGGGAGGACTATAACAGGGATCTACTGGTCAGCACAACTACAGCTCATGGGTGCGACACCATAGCTAGGTGCCAGTGCACGACAGGGGTGTACTTCTGCCTGAGCAGAAACAAACACTACCCAGTGTCATTTGAAGGTCCAGGATTGGTTGAGGTTCAAGAGAGTGAGTATTACCCAAGAAGGTACCAATCCCACGTGCTTCTTGCAGCCGGATTTTCTGAACCTGGAGATTGTGGTGGTATCTTGAGGTGTGAGCATGGTGTTATTGGTATAGTGACCATGGGAGGTGAAGGTGTCGTTGGTTTCGCCGATGTGCGAGACCTTCTATGGCTAGAGGATGACGCCATGGAGCAGGGAGTCAAGGACTACGTGGAACAGCTCGGCAACGCCTTTGGTTCAGGTTTCACCAATCAGATTTGTGAGCAGGTCAATCTCCTGAAAGAGTCCTTGGTAGGTCAAGACTCCATCTTGGAAAAGTCTTTAAAAGCACTAGTAAAAATCATATCAGCATTAGTGATCGTGGTAAGGAACCACGACGACTTGATCACAGTGACTGCTACACTAGCCCTCATTGGCTGCACCTCTTCACCATGGCGATGGCTCAAGCAGAAGGTATCACAATATTATGGAATACCCATGGCCGAGCGTCAGAACAATGGATGGCTCAAGAAATTCACTGAGATGACTAACGCCTGCAAAGGCATGGAGTGGATTGCCATTAAAATTCAGAAATTTATTGAATGGCTGAAAGTTAAGATTCTACCTGAAGTAAAAGAAAAACATGAATTTCTCAATAGATTAAAACAGCTGCCACTTCTTGAAAGTCAGATTGCTACCATAGAACAGAGCGCACCATCACAAGGTGACCAAGAACAGCTCTTCTCCAATGTGCAGTATTTTGCCCACTATTGCAGAAAGTACGCACCTCTGTATGCCGCTGAAGCAAAAAGAGTGTTCTCGTTGGAGAAAAAGATGAGCAACTACATACAGTTCAAGTCCAAATGCCGTATTGAGCCTGTATGTTTACTTCTCCATGGCAGCCCAGGAGCGGGGAAATCCGTGGCTACAAACCTAATTGGTAGATCCCTCGCGGAGAAACTTAACAGCTCTGTGTACTCGTTACCACCAGACCCAGATCATTTTGATGGATACAAACAACAAGCCGTAGTGATCATGGATGACCTGTGCCAGAATCCAGATGGGAAGGATGTGTCACTATTCTGTCAAATGGTATCCAGCGTGGACTTCGTACCACCCATGGCAGCTCTGGAGGAGAAAGGGATTCTTTTCACGTCCCCGTTTGTGCTAGCATCAACCAATGCGGGGTCTATCAATGCACCCACTGTGTCTGACAGCAGGGCACTTGCCAGAAGGTTCCACTTTGATATGAACATCGAGGTGATCTCCATGTATAGCCAGAATGGGAAGATTAACATGCCCATGTCTGTCAAAACATGTGATGAGGATTGCTGCCCGGTCAATTTCAAGAAATGCTGCCCGCTGGTGTGTGGTAAGGCCATTCAATTTATTGACAGAAAGACCCAAGTTAGGTATTCACTGGACATGTTGGTCACCGAGATGTTCAGGGAGTACAACCACAGACACAGCGTGGGTGCCACCCTCGAGGCTTTGTTCCAAGGGCCACCGGTCTACAGGGAGATTAAGATCAGTGTCGCTCCAGAAACACCCCCTCCACCAGCAATCGCTGACCTGCTAAAATCAGTAGACATTGAGGCAGTAAGGGAGTACTGCAAGGAAAAAGGCTGGCTTGTGCCGGAAATTAGCTCCACCCTACAGATTGAGAAGCACGTCAGTAGAGCATTTATCTGCCTACAGGCTCTGACTACATTTGTCTCAGTAGCTGGCATAATCTACATTATCTACAAATTGTTTGCCGGTTTTCAGGGCGCGTATACGGGGATGCCAAATCAGAAACCCAAGGTGCCCACTCTGAGACAGGCTAAGGTGCAGGGCCCGGCATTCGAGTTCGCCGTGGCGATGATGAAGAGAAACGCCAGCACAGTGAAAACAGAATATGGTGAGTTCACCATGCTCGGCATCTATGACAGATGGGCAGTGTTACCACGCCACGCCAAGCCCGGACCGACCATCTTAATGAATGATCAGGAGGTCGGTGTGCTAGATGCCAAAGAATTGGTTGACAAAGATGGGACAAATCTGGAGTTGACTCTCCTAAAGCTCAATCGCAATGAGAAGTTTAGGGATATCAGAGGGTTTCTGGCAAGAGAAGAAGCTGAGGTGAATGAGGCTGTTTTGGCAATAAACACAAGCAAGTTCCCCAACATGTACATACCCGTAGGTCAAGTCACCGACTACGGTTTTCTGAACTTGGGAGGAACGCCCACAAAGAGGATGCTCATGTACAATTTCCCAACTAGAGCAGGCCAATGTGGCGGTGTCCTCATGTCAACAGGGAAGGTTCTAGGAATACATGTAGGCGGAAATGGACACCAAGGATTCTCTGCTGCCCTCCTTAGACATTACTTCAATGAGGAACAAGGTGAGATAGAATTCATTGAGAGCTCAAAGGACGCAGGCTTCCCTGTGATCAACACCCCCAGCAAAACCAAGCTGGAACCAAGCGTGTTTCACCAGGTGTTTGAGGGCAACAAAGAGCCGGCAGTGCTTAGAAATGGGGATCCACGACTCAAGGTCAACTTTGAGGAGGCAATCTTCTCCAAGTACATTGGCAATGTTAACACCCACGTGGACGAATACATGCAAGAGGCCGTGGACCATTATGCAGGGCAGCTAGCTACACTGGACATCAGCACAGAGCCCATGAAACTGGAGGATGCCGTGTATGGTACAGAGGGGCTGGAAGCACTAGACCTAACCACCAGTGCAGGCTATCCGTACGTGGCCCTAGGTATCAAGAAAAGAGACATTCTCTCTAAGAAGACCAAAGACCTTACCAAGTTGAAGGAATGCATGGACAAGTATGGCCTAAACTTACCAATGGTAACTTACGTCAAAGATGAATTAAGATCTGCCGAGAAGGTAGCCAAGGGAAAGTCCAGACTTATTGAGGCCTCCAGTCTCAATGACTCAGTAGCAATGAGGCAAACATTTGGAAACCTGTACAAAACCTTTCATCTCAATCCGGGCATTGTCACGGGCAGTGCTGTTGGGTGTGACCCAGATGTATTTTGGAGTAAGATCCCTGTCATGCTTGATGGACATCTCATAGCTTTTGACTATTCAGGTTATGACGCCAGTCTCAGCCCGGTGTGGTTTGCATGTCTGAAACTCCTCCTAGAGAAACTAGGGTATACGAATAAGGAAACAAACTACATAGATTACCTCTGCAACTCTCACCACTTATATAGGGACAAGCACTACTTTGTGAGAGGCGGTATGCCATCAGGATGTTCGGGCACTAGCATATTTAATTCCATGATTAACAACATTATAATCAGGACTCTCATGCTGAAAGTTTATAAAGGCATTGATTTGGACCAATTCAGAATGATCGCTTATGGGGATGATGTGATTGCCTCCTACCCGTGGCCCATCGATGCGTCACTGTTAGCTGAAGCAGGAAAAGATTATGGATTGATCATGACCCCAGCAGACAAAGGTGAGTGCTTTAATGAGGTAACCTGGACAAATGTGACCTTTTTGAAAAGGTACTTCAGAGCAGATGAACAGTACCCATTCCTGGTCCATCCTGTTATGCCAATGAAGGACATACATGAGTCCATTAGATGGACTAAAGACCCCAAAAACACACAGGATCACGTGCGCTCGCTGTGCCTATTGGCTTGGCACAACGGGGAGCACGAATATGAGGAGTTTATTCGCAAGATCAGAAGCGTGCCCGTCGGGCGCTGCTTGACCCTTCCTGCATTTTCGACACTGCGTAGGAAGTGGTTGGACTCCTTCTAAAATTAGAGCACAATTAGTAGATTACAATTGGCTTAACCCTACCGCATGAACCGAACTTGATAAAAGTGCGGTAGGGGTAAATTCTCCGCATTCGGTGCGG |
| >2E1_Barcode03_EV1_consensus | TTTGTGCGCCTGTTTTATACTCCCCCCCCTAAGGAAACTTTAGAAGCAAAGCAATTGTGATCAATAGTGGGTATGGCACACCAGTCATATCTTGATCAAGCACTTCTGTTCCCCCGGACTTAGTACCAATAGACTGCTCAAGCGGTTGAAGGGGAAAACGTTCGTTATCCGGCCAACTACTTCGAGAAACCTAGTAGCACCATGAAAGTTGCGGAGTGTTTCGCTCAGCACTTCCCCCGTGTAGATCAGGCTGATGAGTCACCGTATTCCCCACGGGCGACCGTGACGGTGGCTGCGTTGGCGGCCTGCCCATGGGGTAACCCATGGGACGCTCTAAAACAGACACGGTGCGAAGAGTCTATTGAGCTAGTTGGTAGTCCTCCGGCCCCTGAATGCGGCCAATCCTAACTGCGGAGCACATACTCCCAATCCAGGGAGCAGTGTGTCGTAATGGGTAACTCTGCAGCGGAACCGACTACTTTGGGTGTCCGTGTTTCCTTTTATTCTCACATTGACTGCTTATGGTGACAATTGAAAGATTGTTACCATATAGCTATTGGATTGGTCATCCGGTGAGCAATAGAGCTATTGTTTATCAATTTGTTGGATTTGTACCACTCAACTTTTCTGTTTTGAGAACACTCAACTACATCTTACTGCTAAACACATCAAAATGGGAGCACAGGTATCAACACAGAAGACCGGGGCGCACGAGACTAGCTTGAGCGCTACTGGTAACTCCATAATACACTACACGAATATTAATTATTACAAAGATGCAGCCTCTAACTCTGCCAATAGACAAGATTTCACCCAAGACCCTGGTAAGTTTACTGAACCAGTGAAAGATGTCATGATAAAAACCCTGCCAGCGCTGAATTCTCCAACGGTTGAAGAGTGCGGGTACAGTGACAGGGTCAGGTCAATCACACTTGGGAACTCCACTATTACAACTCAAGAGTGTGCCAATGTGGTGGTGGGGTACGGTGAATGGCCTGAGTATCTGAGTGATAACGAGGCAACTGCTGAGGACCAACCAACGCAGCCGGACGTGGCCACTTGCCGTTTTTACACCCTAGACTCAGTCCAATGGGAGAATGGGTCACCAGGTTGGTGGTGGAAGTTTCCCGACGCTCTAAGGGATATGGGATTATTTGGCCAAAATATGTACTACCATTACTTAGGCAGAGCCGGGTATACCATCCACGTACAATGCAATGCTTCCAAGTTTCATCAAGGCTGTATCCTGGTAGTGTGTGTCCCTGAGGCGGAGATGGGAAGTGCCCAAACCTCAGGGGTGGTCAACTACGAACACATTAGTAAGGGTGAGATCGCATCAAGGTTCACTACCACGACAACAGCAGAAGACCATGGCGTGCAGGCCGCGGTATGGAATGCTGGTATGGGCGTTGGAGTTGGGAACTTGACGATCTTCCCGCACCAATGGATCAACCTTCGCACCAACAACAGCGCCACAATTGTTATGCCATACGTAAATAGTGTACCAATGGACAATATGTATAGACATCACAACTTTACACTAATGATAATACCCTTTGTGCCTCTGGATTTCAGCGCGGGTGCATCCACATACGTGCCCATAACGGTGACAGTGGCCCCCATGTGTGCCGAGTACAATGGACTACGACTAGCTGGACACCAAGGACTACCGACCATGAACACCCCTGGCAGCAACCAATTTCTTACATCGGACGATTTCCAATCCCCGTCAGCGATGCCTCAATTTGATGTAACTCCAGAAATGCACATCCCTGGTGAGGTGCGCAACCTCATGGAAATTGCCGAAGTTGATTCTGTAATGCCAATTAACAATGATAGCGCCGCAAAAGTTTCATCCATGGAGGCTTATAGAGTCGAATTGAGCACCAACACTAATGCCGGGACTCAAGTGTTTGGCTTTCAACTGAACCCCGGAGCGGAATCAGTAATGAACCGCACATTAATGGGTGAAATCCTAAATTACTACGCACACTGGTCAGGAAGCATAAAGATAACATTCGTGTTCTGTGGTTCTGCCATGACCACTGGCAAGTTTCTGCTGTCTTACGCCCCACCAGGTGCAGGTGCGCCAAAAACTCGCAAGGATGCCATGTTAGGCACTCATGTGGTGTGGGATGTTGGGCTCCAATCCAGCTGCGTGTTATGCATCCCCTGGATTAGTCAAACCCATTACAGATTTGTGGAAAAGGATCCATACACCAATGCCGGGTTTGTGACATGTTGGTATCAGACCAGTGTAGTGTCCCCAGCGAGCAACCAGCCAAAGTGTTATATGATGTGCATGGTTTCTGCGTGTAATGACTTCTCAGTTCGCATGTTGAGAGATACCAAGTTCATTGAGCAAACATCTTTTTACCAAGGTGATGTGCAGAATGCTGTCGAAGGGGCTATGGTCAGGGTGGCAGATACAGTGCAAACTTCAGCCACAAACTCAGAGAGGGTGCCTAACTTGACAGCAGTAGAAACTGGTCACACTTCGCAGGTAGTACCTGGTGATACCATGCAGACTAGACATGTGATCAACAATCACGTGAGGTCAGAATCTACAATTGAGAACTTCCTTGCCAGATCAGCGTGTGTTTTCTTCCTAGAGTACAAGACAGGGACCAAAGAGGATTCCAATAGCTTCAACAATTGGGTGATTACAACCAGGCGAGTGGCTCAACTACGTAGAAAACTGGAAATGTTTACTTACCTACGGTTTGACATGGAAATCACCGTGGTCATTACAAGCTCGCAAGATCAGTCTACATCACAAAACCAGAATGCACCAGTGCTAACACACCAGATAATGTATGTACCACCAGGGGGACCCATACCCATAAGCGTGGATGATTACAGCTGGCAAACATCCACCAACCCCAGTATCTTTTGGACCGAAGGGAACGCTCCGGCACGCATGTCAATTCCATTTATTAGCATAGGCAATGCGTATAGTAATTTCTACGATGGGTGGTCTCACTTCTCCCAGAATGGCGTGTATGGCTTCACTACTCTGAACAACATGGGTCAATTGTTCTTCCGGCACGTAAACAAGCCCAACCCAGCCGCTATTACAAGTGTGGCGCGCATTTACTTCAAACCGAAACATGTACGCGCTTGGGTGCCTAGACCACCGCGCTTGTGTCCATACATCAATAGCACGAATGTCAACTTTGAACCCAAGCCAGTGACTGAAGTACGTACCAACATAATAACAACGGGTGCCTTTGGGCAGCAATCTGGCGCAGTGTACGTGGGCAACTACAGAGTGGTCAATAGGCACTTGGCGACTCACATTGATTGGCAAAACTGTGTGTGGGAGGACTATAACAGGGATCTACTGGTCAGCACAACTACAGCTCATGGGTGCGACACCATAGCTAGGTGCCAGTGCACGACAGGGGTGTACTTCTGCCTGAGCAGAAACAAACACTACCCAGTGTCATTTGAAGGTCCAGGATTGGTTGAGGTTCAAGAGAGTGAGTATTACCCAAGAAGGTACCAATCCCACGTGCTTCTTGCAGCCGGATTTTCTGAACCTGGAGATTGTGGTGGTATCTTGAGGTGTGAGCATGGTGTTATTGGTATAGTGACCATGGGAGGTGAAGGTGTCGTTGGTTTCGCCGATGTGCGAGACCTTCTATGGCTAGAGGATGACGCCATGGAGCAGGGAGTCAAGGACTACGTGGAACAGCTCGGCAACGCCTTTGGTTCAGGTTTCACCAATCAGATTTGTGAGCAGGTCAATCTCCTGAAAGAGTCCTTGGTAGGTCAAGACTCCATCTTGGAAAAGTCTTTAAAAGCACTAGTAAAAATCATATCAGCATTAGTGATCGTGGTAAGGAACCACGACGACTTGATCACAGTGACTGCTACACTAGCCCTCATTGGCTGCACCTCTTCACCATGGCGATGGCTCAAGCAGAAGGTATCACAATATTATGGAATACCCATGGCCGAGCGTCAGAACAATGGATGGCTCAAGAAATTCACTGAGATGACTAACGCCTGCAAAGGCATGGAGTGGATTGCCATTAAAATTCAGAAATTTATTGAATGGCTGAAAGTTAAGATTCTACCTGAAGTAAAAGAAAAACATGAATTTCTCAATAGATTAAAACAGCTGCCACTTCTTGAAAGTCAGATTGCTACCATAGAACAGAGCGCACCATCACAAGGTGACCAAGAACAGCTCTTCTCCAATGTGCAGTATTTTGCCCACTATTGCAGAAAGTACGCACCTCTGTATGCCGCCGAAGCAAAAAGAGTGTTCTCGTTGGAGAAAAAGATGAGCAACTACATACAGTTCAAGTCCAAATGCCGTATTGAGCCTGTATGTTTACTTCTCCATGGCAGCCCAGGAGCGGGGAAATCCGTGGCTACAAACCTAATTGGTAGATCCCTCGCGGAGAAACTTAACAGCTCTGTGTACTCGTTACCACCAGACCCAGATCATTTTGATGGATACAAACAACAAGCCGTAGTGATCATGGATGACCTGTGCCAGAATCCAGATGGGAAGGATGTGTCACTATTCTGTCAAATGGTATCCAGCGTGGACTTCGTACCACCCATGGCAGCTCTGGAGGAGAAAGGGATTCTTTTCACGTCCCCGTTTGTGCTAGCATCAACCAATGCGGGGTCTATCAATGCACCCACTGTGTCTGACAGCAGGGCACTTGCCAGAAGGTTCCACTTTGATATGAACATCGAGGTGATCTCCATGTATAGCCAGAATGGGAAGATTAACATGCCCATGTCTGTCAAAACATGTGATGAGGATTGCTGCCCGGTCAATTTCAAGAAATGCTGCCCGCTGGTGTGTGGTAAGGCCATTCAATTTATTGACAGAAAGACCCAAGTTAGGTATTCACTGGACATGTTGGTCACCGAGATGCTCAGGGAGTACAACCACAGACACAGCGTGGGTGCCACCCTCGAGGCTTTGTTCCAAGGGCCACCGGTCTACAGGGAGATTAAGATCAGTGTCGCTCCAGAAACACCCCCTCCACCAGCAATCGCTGACCTGCTAAAATCAGTAGACAGTGAGGCAGTAAGGGAGTACTGCAAGGAAAAAGGCTGGCTTGTGCCGGAAATTAGCTCCACCCTACAGATTGAGAAGCACGTCAGTAGAGCATTTATCTGCCTACAGGCTCTGACTACATTTGTCTCAGTAGCTGGCATAATCTACATTATCTACAAATTGTTTGCCGGTTTTCAGGGCGCGTATACGGGGATGCCAAATCAGAAACCCAAGGTGCCCACTCTGAGACAGGCTAAGGTGCAGGGCCCGGCATTCGAGTTCGCCGTGGCGATGATGAAGAGAAACGCCAGCACAGTGAAAACAGAATATGGTGAGTTCACCATGCTCGGCATCTATGACAGATGGGCAGTGTTACCACGCCACGCCAAGCCCGGACCGACCATCTTAATGAATGATCAGGAGGTCGGTGTGCTAGATGCCAAAGAATTGGTTGACAAAGATGGGACAAATCTGGAGTTGACTCTCCTAAAGCTCAATCGCAATGAGAAGTTTAGGGATATCAGAGGGTTTCTGGCAAGAGAAGAAGCTGAGGTGAATGAGGCTGTTTTGGCAATAAACACAAGCAAGTTCCCCAACATGTACATACCCGTAGGTCAAGTCACCGACTACGGTTTTCTGAACTTGGGAGGAACGCCCACAAAGAGGATGCTCATGTACAATTTCCCAACTAGAGCAGGCCAATGTGGCGGTGTCCTCATGTCAACAGGGAAGGTTCTAGGAATACATGTAGGCGGAAATGGACACCAAGGATTCTCTGCTGCCCTCCTTAGACATTACTTCAATGAGGAACAAGGTGAGATAGAATTCATTGAGAGCTCAAAGGACGCAGGCTTCCCTGTGATCAACACCCCCAGCAAAACCAAGCTGGAACCAAGCGTGTTTCACCAGGTGTTTGAGGGCAACAAAGAGCCGGCAGTGCTTAGAAATGGGGATCCACGACTCAAGGTCAACTTTGAGGAGGCAATCTTCTCCAAGTACATTGGCAATGTTAACACCCACGTGGACGAATACATGCAAGAGGCCGTGGACCATTATGCAGGGCAGCTAGCTACACTGGACATCAGCACAGAGCCCATGAAACTGGAGGATGCCGTGTATGGTACAGAGGGGCTGGAAGCACTAGACCTAACCACCAGTGCAGGCTATCCGTACGTGGCCCTAGGTATCAAGAAAAGAGACATTCTCTCTAAGAAGACCAAAGACCTTACCAAGTTGAAGGAATGCATGGACAAGTATGGCCTAAACTTACCAATGGTAACTTACGTCAAAGATGAATTAAGATCTGCCGAGAAGGTAGCCAAGGGAAAGTCCAGACTTATTGAGGCCTCCAGTCTCAATGACTCAGTAGCAATGAGGCAAACATTTGGAAACCTGTACAAAACCTTTCATCTCAATCCGGGCATTGTCACGGGCAGTGCTGTTGGGTGTGACCCAGATGTATTTTGGAGTAAGATCCCTGTCATGCTTGATGGACATCTCATAGCTTTTGACTATTCAGGTTATGACGCCAGTCTCAGCCCGGTGTGGTTTGCATGTCTGAAACTCCTCCTAGAGAAACTAGGGTATACGAATAAGGAAACAAACTACATAGATTACCTCTGCAACTCTCACCACTTATATAGGGACAAGCACTACTTTGTGAGAGGCGGTATGCCATCAGGATGTTCGGGCACTAGCATATTTAATTCCATGATTAACAACATTATAATCAGGACTCTCATGCTGAAAGTTTATAAAGGCATTGATTTGGACCAATTCAGAATGATCGCTTATGGGGATGATGTGATTGCCTCCTACCCGTGGCCCATCGATGCGTCACTGTTAGCTGAAGCAGGAAAAGATTATGGATTGATCATGACCCCAGCAGACAAAGGTGAGTGCTTTAATGAGGTAACCTGGACAAATGTGACCTTTTTGAAAAGGTACTTCAGAGCAGATGAACAGTACCCATTCCTGGTCCATCCTGTTATGCCAATGAAGGACATACATGAGTCCATTAGATGGACTAAAGACCCCAAAAACACACAGGATCACGTGCGCTCGCTGTGCCTATTGGCTTGGCACAACGGGGAGCACGAATATGAGGAGTTTATTCGCAAGATCAGAAGCGTGCCCGTCGGGCGCTGCTTGACCCTTCCTGCATTTTCGACACTGCGTAGGAAGTGGTTGGACTCCTTCTAAAATTAGAGCACAATTAGTAGATTACAATTGGCTTAACCCTACCGCATGAACCGAACTTGATAAAAGTGCGGTAGGGGTAAATTCTCCGCATTCGGTGCGG |
| >2E1V_Barcode04_EV1_consensus | TTTGTGCGCCTGTTTTATACTCCCCCCCCTAAGGAAACTTTAGAAGCAAAGCAATTGTGATCAATAGTGGGTATGGCACACCAGTCATATCTTGATCAAGCACTTCTGTTCCCCCGGACTTAGTACCAATAGACTGCTCAAGCGGTTGAAGGGGAAAACGTTCGTTATCCGGCCAACTACTTCGAGAAACCTAGTAGCACCATGAAAGTTGCGGAGTGTTTCGCTCAGCACTTCCCCCGTGTAGATCAGGCTGATGAGTCACCGTATTCCCCACGGGCGACCGTGACGGTGGCTGCGTTGGCGGCCTGCCCATGGGGTAACCCATGGGACGCTCTAAAACAGACACGGTGCGAAGAGTCTATTGAGCTAGTTGGTAGTCCTCCGGCCCCTGAATGCGGCCAATCCTAACTGCGGAGCACATACTCCCAATCCAGGGAGCAGTGTGTCGTAATGGGTAACTCTGCAGCGGAACCGACTACTTTGGGTGTCCGTGTTTCCTTTTATTCTCACATTGACTGCTTATGGTGACAATTGAAAGATTGTTACCATATAGCTATTGGATTGGTCATCCGGTGAGCAATAGAGCTATTGTTTATCAATTTGTTGGATTTGTACCACTCAACTTTTCTGTTTTGAGAACACTCAACTACATCTTACTGCTAAACACATCAAAATGGGAGCACAGGTATCAACACAGAAGACCGGGGCGCACGAGACTAGCTTGAGCGCTACTGGTAACTCCATAATACACTACACGAATATTAATTATTACAAAGATGCAGCCTCTAACTCTGCCAATAGACAAGATTTCACCCAAGACCCTGGTAAGTTTACTGAACCAGTGAAAGATGTCATGATAAAAACCCTGCCAGCGCTGAATTCTCCAACGGTTGAAGAGTGCGGGTACAGTGACAGGGTCAGGTCAATCACACTTGGGAACTCCACTATTACAACTCAAGAGTGTGCCAATGTGGTGGTGGGGTACGGTGAATGGCCTGAGTATCTGAGTGATAACGAGGCAACTGCTGAGGACCAACCAACGCAGCCGGACGTGGCCACTTGCCGTTTTTACACCCTAGACTCAGTCCAATGGGAGAATGGGTCACCAGGTTGGTGGTGGAAGTTTCCCGACGCTCTAAGGGATATGGGATTATTTGGCCAAAATATGTACTACCATTACTTAGGCAGAGCCGGGTATACCATCCACGTACAATGCAATGCTTCCAAGTTTCATCAAGGCTGTATCCTGGTAGTGTGTGTCCCTGAGGCGGAGATGGGAAGTGCCCAAACCTCAGGGGTGGTCAACTACGAACACATTAGTAAGGGTGAGATCGCATCAAGGTTCACTACCACGACAACAGCAGAAGACCATGGCGTGCAGGCCGCGGTATGGAATGCTGGTATGGGCGTTGGAGTTGGGAACTTGACGATCTTCCCGCACCAATGGATCAACCTTCGCACCAACAACAGCGCCACAATTGTTATGCCATACGTAAATAGTGTACCAATGGACAATATGTATAGACATCACAACTTTACACTAATGATAATACCCTTTGTGCCTCTGGATTTCAGCGCGGGTGCATCCACATACGTGCCCATAACGGTGACAGTGGCCCCCATGTGTGCCGAGTACAATGGACTACGACTAGCTGGACACCAAGGACTACCGACCATGAACACCCCTGGCAGCAACCAATTTCTTACATCGGACGATTTCCAATCCCCGTCAGCGATGCCTCAATTTGATGTAACTCCAGAAATGCACATCCCTGGTGAGGTGCGCAACCTCATGGAAATTGCCGAAGTTGATTCTGTAATGCCAATTAACAATGATAGCGCCGCAAAAGTTTCATCCATGGAGGCTTATAGAGTCGAATTGAGCACCAACACTAATGCCGGGACTCAAGTGTTTGGCTTTCAACTGAACCCCGGAGCGGAATCAGTAATGAACCGCACATTAATGGGTGAAATCCTAAATTACTACGCACACTGGTCAGGAAGCATAAAGATAACATTCGTGTTCTGTGGTTCTGCCATGACCACTGGCAAGTTTCTGCTGTCTTACGCCCCACCAGGTGCAGGTGCGCCAAAAACTCGCAAGGATGCCATGTTAGGCACTCATGTGGTGTGGGATGTTGGGCTCCAATCCAGCTGCGTGTTATGCATCCCCTGGATTAGTCAAACCCATTACAGATTTGTGGAAAAGGATCCATACACCAATGCCGGGTTTGTGACATGTTGGTATCAGACCAGTGTAGTGTCCCCAGCGAGCAACCAGCCAAAGTGTTATATGATGTGCATGGTTTCTGCGTGTAATGACTTCTCAGTTCGCATGTTGAGAGATACCAAGTTCATTGAGCAAACATCTTTTTACCAAGGTGATGTGCAGAATGCTGTCGAAGGGGCTATGGTCAGGGTGGCAGATACAGTGCAAACTTCAGCCACAAACTCAGAGAGGGTGCCTAACTTGACAGCAGTAGAAACTGGTCACACTTCGCAGGTAGTACCTGGTGATACCATGCAGACTAGACATGTGATCAACAATCACGTGAGGTCAGAATCTACAATTGAGAACTTCCTTGCCAGATCAGCGTGTGTTTTCTTCCTAGAGTACAAGACAGGGACCAAAGAGGATTCCAATAGCTTCAACAATTGGGTGATTACAACCAGGCGAGTGGCTCAACTACGTAGAAAACTGGAAATGTTTACTTACCTACGGTTTGACATGGAAATCACCGTGGTCATTACAAGCTCGCAAGATCAGTCTACATCACAAAACCAGAATGCACCAGTGCTAACACACCAGATAATGTATGTACCACCAGGGGGACCCATACCCATAAGCGTGGATGATTACAGCTGGCAAACATCCACCAACCCCAGTATCTTTTGGACCGAAGGGAACGCTCCGGCACGCATGTCAATTCCATTTATTAGCATAGGCAATGCGTATAGTAATTTCTACGATGGGTGGTCTCACTTCTCCCAGAATGGCGTGTATGGCTTCACTACTCTGAACAACATGGGTCAATTGTTCTTCCGGCACGTAAACAAGCCCAACCCAGCCGCTATTACAAGTGTGGCGCGCATTTACTTCAAACCGAAACATGTACGCGCTTGGGTGCCTAGACCACCGCGCTTGTGTCCATACATCAATAGCACGAATGTCAACTTTGAACCCAAGCCAGTGACTGAAGTACGTACCAACATAATAACAACGGGTGCCTTTGGGCAGCAATCTGGCGCAGTGTACGTGGGCAACTACAGAGTGGTCAATAGGCACTTGGCGACTCACATTGATTGGCAAAACTGTGTGTGGGAGGACTATAACAGGGATCTACTGGTCAGCACAACTACAGCTCATGGGTGCGACACCATAGCTAGGTGCCAGTGCACGACAGGGGTGTACTTCTGCCTGAGCAGAAACAAACACTACCCAGTGTCATTTGAAGGTCCAGGATTGGTTGAGGTTCAAGAGAGTGAGTATTACCCAAGAAGGTACCAATCCCACGTGCTTCTTGCAGCCGGATTTTCTGAACCTGGAGATTGTGGTGGTATCTTGAGGTGTGAGCATGGTGTTATTGGTATAGTGACCATGGGAGGTGAAGGTGTCGTTGGTTTCGCCGATGTGCGAGACCTTCTATGGCTAGAGGATGACGCCATGGAGCAGGGAGTCAAGGACTACGTGGAACAGCTCGGCAACGCCTTTGGTTCAGGTTTCACCAATCAGATTTGTGAGCAGGTCAATCTCCTGAAAGAGTCCTTGGTAGGTCAAGACTCCATCTTGGAAAAGTCTTTAAAAGCACTAGTAAAAATCATATCAGCATTAGTGATCGTGGTAAGGAACCACGACGACTTGATCACAGTGACTGCTACACTAGCCCTCATTGGCTGCACCTCTTCACCATGGCGATGGCTCAAGCAGAAGGTATCACAATATTATGGAATACCCATGGCCGAGCGTCAGAACAATGGATGGCTCAAGAAATTCACTGAGATGACTAACGCCTGCAAAGGCATGGAGTGGATTGCCATTAAAATTCAGAAATTTATTGAATGGCTGAAAGTTAAGATTCTACCTGAAGTAAAAGAAAAACATGAATTTCTCAATAGATTAAAACAGCTGCCACTTCTTGAAAGTCAGATTGCTACCATAGAACAGAGCGCACCATCACAAGGTGACCAAGAACAGCTCTTCTCCAATGTGCAGTATTTTGCCCACTATTGCAGAAAGTACGCACCTCTGTATGCCGCCGAAGCAAAAAGAGTGTTCTCGTTGGAGAAAAAGATGAGCAACTACATACAGTTCAAGTCCAAATGCCGTATTGAGCCTGTATGTTTACTTCTCCATGGCAGCCCAGGAGCGGGGAAATCCGTGGCTACAAACCTAATTGGTAGATCCCTCGCGGAGAAACTTAACAGCTCTGTGTACTCGTTACCACCAGACCCAGATCATTTTGATGGATACAAACAACAGGCCGTAGTGATCATGGATGACCTGTGCCAGAATCCAGATGGGAAGGATGTGTCACTATTCTGTCAAATGGTATCCAGCGTGGACTTCGTACCACCCATGGCAGCTCTGGAGGAGAAAGGGATTCTTTTCACGTCCCCGTTTGTGCTAGCATCAACCAATGCGGGGTCTATCAATGCACCCACTGTGTCTGACAGCAGGGCACTTGCCAGAAGGTTCCACTTTGATATGAACATCGAGGTGATCTCCATGTATAGCCAGAATGGGAAGATTAACATGCCCATGTCTGTCAAAACATGTGATGAGGATTGCTGCCCGGTCAATTTCAAGAAATGCTGCCCGCTGGTGTGTGGTAAGGCCATTCAATTTATTGACAGAAAGACCCAAGTTAGGTATTCACTGGACATGTTGGTCACCGAGATGCTCAGGGAGTACAACCACAGACACAGCGTGGGTGCCACCCTCGAGGCTTTGTTCCAAGGGCCACCGGTCTACAGGGAGATTAAGATCAGTGTCGCTCCAGAAACACCCCCTCCACCAGCAATCGCTGACCTGCTAAAATCAGTAGACAGTGAGGCAGTAAGGGAGTACTGCAAGGAAAAAGGCTGGCTTGTGCCGGAAATTAGCTCCACCCTACAGATTGAGAAGTACGTCAGTAGAGCATTTATCTGCCTACAGGCTCTGACTACATTTGTCTCAGTAGCTGGCATAATCTACATTATCTACAAATTGTTTGCCGGTTTTCAGGGCGCGTATACGGGGATGCCAAATCAGAAACCCAAGGTGCCCACTCTGAGACAGGCTAAGGTGCAGGGCCCGGCATTCGAGTTCGCCGTGGCGATGATGAAGAGAAACGCCAGCACAGTGAAAACAGAATATGGTGAGTTCACCATGCTCGGCATCTATGACAGATGGGCAGTGTTACCACGCCACGCCAAGCCCGGACCGACCATCTTAATGAATGATCAGGAGGTCGGTGTGCTAGATGCCAAAGAATTGGTTGACAAAGATGGGACAAATCTGGAGTTGACTCTCCTAAAGCTCAATCGCAATGAGAAGTTTAGGGATATCAGAGGGTTTCTGGCAAGAGAAGAAGCTGAGGTGAATGAGGCTGTTTTGGCAATAAACACAAGCAAGTTCCCCAACATGTACATACCCGTAGGTCAAGTCACCGACTACGGTTTTCTGAACTTGGGAGGAACGCCCACAAAGAGGATGCTCATGTACAATTTCCCAACTAGAGCAGGCCAATGTGGCGGTGTCCTCATGTCAACAGGGAAGGTTCTAGGAATACATGTAGGCGGAAATGGACACCAAGGATTCTCTGCTGCCCTCCTTAGACATTACTTCAATGAGGAACAAGGTGAGATAGAATTCATTGAGAGCTCAAAGGACGCAGGCTTCCCTGTGATCAACACCCCCAGCAAAACCAAGCTGGAACCAAGCGTGTTTCACCAGGTGTTTGAGGGCAACAAAGAGCCGGCAGTGCTTAGAAATGGGGATCCACGACTCAAGGTCAACTTTGAGGAGGCAATCTTCTCCAAGTACATTGGCAATGTTAACACCCACGTGGACGAATACATGCAAGAGGCCGTGGACCATTATGCAGGGCAGCTAGCTACACTGGACATCAGCACAGAGCCCATGAAACTGGAGGATGCCGTGTATGGTACAGAGGGGCTGGAAGCACTAGACCTAACCACCAGTGCAGGCTATCCGTACGTGGCCCTAGGTATCAAGAAAAGAGACATTCTCTCTAAGAAGACCAAAGACCTTACCAAGTTGAAGGAATGCATGGACAAGTATGGCCTAAACTTACCAATGGTAACTTACGTCAAAGATGAATTAAGATCTGCCGAGAAGGTAGCCAAGGGAAAGTCCAGACTTATTGAGGCCTCCAGTCTCAATGACTCAGTAGCAATGAGGCAAACATTTGGAAACCTGTACAAAACCTTTCATCTCAATCCGGGCATTGTCACGGGCAGTGCTGTTGGGTGTGACCCAGATGTATTTTGGAGTAAGATCCCTGTCATGCTTGATGGACATCTCATAGCTTTTGACTATTCAGGTTATGACGCCAGTCTCAGCCCGGTGTGGTTTGCATGTCTGAAACTCCTCCTAGAGAAACTAGGGTATACGAATAAGGAAACAAACTACATAGATTACCTCTGCAACTCTCACCACTTATATAGGGACAAGCACTACTTTGTGAGAGGCGGTATGCCATCAGGATGTTCGGGCACTAGCATATTTAATTCCATGATTAACAACATTATAATCAGGACTCTCATGCTGAAAGTTTATAAAGGCATTGATTTGGACCAATTCAGAATGATCGCTTATGGGGATGATGTGATTGCCTCCTACCCGTGGCCCATCGATGCGTCACTGTTAGCTGAAGCAGGAAAAGATTATGGATTGATCATGACCCCAGCAGACAAAGGTGAGTGCTTTAATGAGGTAACCTGGACAAATGTGACCTTTTTGAAAAGGTACTTCAGAGCAGATGAACAGTACCCATTCCTGGTCCATCCTGTTATGCCAATGAAGGACATACATGAGTCCATTAGATGGACTAAAGACCCCAAAAACACACAGGATCACGTGCGCTCGCTGTGCCTATTGGCTTGGCACAACGGGGAGCACGAATATGAGGAGTTTATTCGCAAGATCAGAAGCGTGCCCGTCGGGCGCTGCTTGACCCTTCCTGCATTTTCGACACTGCGTAGGAAGTGGTTGGACTCCTTCTAAAATTAGAGCATAATTAGTAGATTACAATTGGCTTAACCCTACCGCATGAACCGAACTTGATAAAAGTGCGGTAGGGGTAAATTCTCCGCATTCGGTGCGG |
| >3E1_Barcode05_EV1_consensus | TTTGTGCGCCTGTTTTATACTCCCCCCCCTAAGGAAACTTTAGAAGCAAAGCAATTGTGATCAATAGTGGGTATGGCACACCAGTCATATCTTGATCAAGCACTTCTGTTCCCCCGGACTTAGTACCAATAGACTGCTCAAGCGGTTGAAGGGGAAAACGTTCGTTATCCGGCCAACTACTTCGAGAAACCTAGTAGCACCATGAAAGTTGCGGAGTGTTTCGCTCAGCACTTCCCCCGTGTAGATCAGGCTGATGAGTCACCGTATTCCCCACGGGCGACCGTGACGGTGGCTGCGTTGGCGGCCTGCCCATGGGGTAACCCATGGGACGCTCTAAAACAGACACGGTGCGAAGAGTCTATTGAGCTAGTTGGTAGTCCTCCGGCCCCTGAATGCGGCCAATCCTAACTGCGGAGCACATACTCCCAATCCAGGGAGCAGTGTGTCGTAATGGGTAACTCTGCAGCGGAACCGACTACTTTGGGTGTCCGTGTTTCCTTTTATTCTCACATTGACTGCTTATGGTGACAATTGAAAGATTGTTACCATATAGCTATTGGATTGGTCATCCGGTGAGCAATAGAGCTATTGTTTATCAATTTGTTGGATTTGTACCACTCAACTTTTCTGTTTTGAGAACACTCAACTACATCTTACTGCTAAACACATCAAAATGGGAGCACAGGTATCAACACAGAAGACCGGGGCGCACGAGACTAGCTTGAGCGCTACTGGTAACTCCATAATACACTACACGAATATTAATTATTACAAAGATGCAGCCTCTAACTCTGCCAATAGACAAGATTTCACCCAAGACCCTGGTAAGTTTACTGAACCAGTGAAAGATGTCATGATAAAAACCCTGCCAGCGCTGAATTCTCCAACGGTTGAAGAGTGCGGGTACAGTGACAGGGTCAGGTCAATCACACTTGGGAACTCCACTATTACAACTCAAGAGTGTGCCAATGTGGTGGTGGGGTACGGTGAATGGCCTGAGTATCTGAGTGATAACGAGGCAACTGCTGAGGACCAACCAACGCAGCCGGACGTGGCCACTTGCCGTTTTTACACCCTAGACTCAGTCCAATGGGAGAATGGGTCACCAGGTTGGTGGTGGAAGTTTCCCGACGCTCTAAGGGATATGGGATTATTTGGCCAAAATATGTACTACCATTACTTAGGCAGAGCCGGGTATACCATCCACGTACAATGCAATGCTTCCAAGTTTCATCAAGGCTGTATCCTGGTAGTGTGTGTCCCTGAGGCGGAGATGGGAAGTGCCCAAACCTCAGGGGTGGTCAACTACGAACACATTAGTAAGGGTGAGATCGCATCAAGGTTCACTACCACGACAACAGCAGAAGACCATGGCGTGCAGGCCGCGGTATGGAATGCTGGTATGGGCGTTGGAGTTGGGAACTTGACGATCTTCCCGCACCAATGGATCAACCTTCGCACCAACAACAGCGCCACAATTGTTATGCCATACGTAAATAGTGTACCAATGGACAATATGTATAGACATCACAACTTTACACTAATGATAATACCCTTTGTGCCTCTGGATTTCAGCGCGGGTGCATCCACATACGTGCCCATAACGGTGACAGTGGCCCCCATGTGTGCCGAGTACAATGGACTACGACTAGCTGGACACCAAGGACTACCGACCATGAACACCCCTGGCAGCAACCAATTTCTTACATCGGACGATTTCCAATCCCCGTCAGCGATGCCTCAATTTGATGTAACTCCAGAAATGCACATCCCTGGTGAGGTGCGCAACCTCATGGAAATTGCCGAAGTTGATTCTGTAATGCCAATTAACAATGATAGCGCCGCAAAAGTTTCATCCATGGAGGCTTATAGAGTCGAATTGAGCACCAACACTAATGCCGGGACTCAAGTGTTTGGCTTTCAACTGAACCCCGGAGCGGAATCAGTAATGAACCGCACATTAATGGGTGAAATCCTAAATTACTACGCACACTGGTCAGGAAGCATAAAGATAACATTCGTGTTCTGTGGTTCTGCCATGACCACTGGCAAGTTTCTGCTGTCTTACGCCCCACCAGGTGCAGGTGCGCCAAAAACTCGCAAGGATGCCATGTTAGGCACTCATGTGGTGTGGGATGTTGGGCTCCAATCCAGCTGCGTGTTATGCATCCCCTGGATTAGTCAAACCCATTACAGATTTGTGGAAAAGGATCCATACACCAATGCCGGGTTTGTGACATGTTGGTATCAGACCAGTGTAGTGTCCCCAGCGAGCAACCAGCCAAAGTGTTATATGATGTGCATGGTTTCTGCGTGTAATGACTTCTCAGTTCGCATGTTGAGAGATACCAAGTTCATTGAGCAAACATCTTTTTACCAAGGTGATGTGCAGAATGCTGTCGAAGGGGCTATGGTCAGGGTGGCAGATACAGTGCAAACTTCAGCCACAAACTCAGAGAGGGTGCCTAACTTGACAGCAGTAGAAACTGGTCACACTTCGCAGGTAGTACCTGGTGATACCATGCAGACTAGACATGTGATCAACAATCACGTGAGGTCAGAATCTACAATTGAGAACTTCCTTGCCAGATCAGCGTGTGTTTTCTTCCTAGAGTACAAGACAGGGACCAAAGAGGATTCCAATAGCTTCAACAATTGGGTGATTACAACCAGGCGAGTGGCTCAACTACGTAGAAAACTGGAAATGTTTACTTACCTACGGTTTGACATGGAAATCACCGTGGTCATTACAAGCTCGCAAGATCAGTCTACATCACAAAACCAGAATGCACCAGTGCTAACACACCAGATAATGTATGTACCACCAGGGGGACCCATACCCATAAGCGTGGATGATTACAGCTGGCAAACATCCACCAACCCCAGTATCTTTTGGACCGAAGGGAACGCTCCGGCACGCATGTCAATTCCATTTATTAGCATAGGCAATGCGTATAGTAATTTCTACGATGGGTGGTCTCACTTCTCCCAGAATGGCGTGTATGGCTTCACTACTCTGAACAACATGGGTCAATTGTTCTTCCGGCACGTAAACAAGCCCAACCCAGCCGCTATTACAAGTGTGGCGCGCATTTACTTCAAACCGAAACATGTACGCGCTTGGGTGCCTAGACCACCGCGCTTGTGTCCATACATCAATAGCACGAATGTCAACTTTGAACCCAAGCCAGTGACTGAAGTACGTACCAACATAATAACAACGGGTGCCTTTGGGCAGCAATCTGGCGCAGTGTACGTGGGCAACTACAGAGTGGTCAATAGGCACTTGGCGACTCACATTGATTGGCAAAACTGTGTGTGGGAGGACTATAACAGGGATCTACTGGTCAGCACAACTACAGCTCATGGGTGCGACACCATAGCTAGGTGCCAGTGCACGACAGGGGTGTACTTCTGCCTGAGCAGAAACAAACACTACCCAGTGTCATTTGAAGGTCCAGGATTGGTTGAGGTTCAAGAGAGTGAGTATTACCCAAGAAGGTACCAATCCCACGTGCTTCTTGCAGCCGGATTTTCTGAACCTGGAGATTGTGGTGGTATCTTGAGGTGTGAGCATGGTGTTATTGGTATAGTGACCATGGGAGGTGAAGGTGTCGTTGGTTTCGCCGATGTGCGAGACCTTCTATGGCTAGAGGATGACGCCATGGAGCAGGGAGTCAAGGACTACGTGGAACAGCTCGGCAACGCCTTTGGTTCAGGTTTCACCAATCAGATTTGTGAGCAGGTCAATCTCCTGAAAGAGTCCTTGGTAGGTCAAGACTCCATCTTGGAAAAGTCTTTAAAAGCACTAGTAAAAATCATATCAGCATTAGTGATCGTGGTAAGGAACCACGACGACTTGATCACAGTGACTGCTACACTAGCCCTCATTGGCTGCACCTCTTCACCATGGCGATGGCTCAAGCAGAAGGTATCACAATATTATGGAATACCCATGGCCGAGCGTCAGAACAATGGATGGCTCAAGAAATTCACTGAGATGACTAACGCCTGCAAAGGCATGGAGTGGATTGCCATTAAAATTCAGAAATTTATTGAATGGCTGAAAGTTAAGATTCTACCTGAAGTAAAAGAAAAACATGAATTTCTCAATAGATTAAAACAGCTGCCACTTCTTGAAAGTCAGATTGCTACCATAGAACAGAGCGCACCATCACAAGGTGACCAAGAACAGCTCTTCTCCAATGTGCAGTATTTTGCCCACTATTGCAGAAAGTACGCACCTCTGTATGCCGCCGAAGCAAAAAGAGTGTTCTCGTTGGAGAAAAAGATGAGCAACTACATACAGTTCAAGTCCAAATGCCGTATTGAGCCTGTATGTTTACTTCTCCATGGCAGCCCAGGAGCGGGGAAATCCGTGGCTACAAACCTAATTGGTAGATCCCTCGCGGAGAAACTTAACAGCTCTGTGTACTCGTTACCACCAGACCCAGATCATTTTGATGGATACAAACAACAAGCCGTAGTGATCATGGATGACCTGTGCCAGAATCCAGATGGGAAGGATGTGTCACTATTCTGTCAAATGGTATCCAGCGTGGACTTCGTACCACCCATGGCAGCTCTGGAGGAGAAAGGGATTCTTTTCACGTCCCCGTTTGTGCTAGCATCAACCAATGCGGGGTCTATCAATGCACCCACTGTGTCTGACAGCAGGGCACTTGCCAGAAGGTTCCACTTTGATATGAACATCGAGGTGATCTCCATGTATAGCCAGAATGGGAAGATTAACATGCCCATGTCTGTCAAAACATGTGATGAGGATTGCTGCCCGGTCAATTTCAAGAAATGCTGCCCGCTGGTGTGTGGTAAGGCCATTCAATTTATTGACAGAAAGACCCAAGTTAGGTATTCACTGGACATGTTGGTCACCGAGATGCTCAGGGAGTACAACCACAGACACAGCGTGGGTGCCACCCTCGAGGCTTTGTTCCAAGGGCCACCGGTCTACAGGGAGATTAAGATCAGTGTCGCTCCAGAAACACCCCCTCCACCAGCAATCGCTGACCTGCTAAAATCAGTAGACAGTGAGGCAGTAAGGGAGTACTGCAAGGAAAAAGGCTGGCTTGTGCCGGAAATTAGCTCCACCCTACAGATTGAGAAGCACGTCAGTAGAGCATTTATCTGCCTACAGGCTCTGACTACATTTGTCTCAGTAGCTGGCATAATCTACATTATCTACAAATTGTTTGCCGGTTTTCAGGGCGCGTATACGGGGATGCCAAATCAGAAACCCAAGGTGCCCACTCTGAGACAGGCTAAGGTGCAGGGCCCGGCATTCGAGTTCGCCGTGGCGATGATGAAGAGAAACGCCAGCACAGTGAAAACAGAATATGGTGAGTTCACCATGCTCGGCATCTATGACAGATGGGCAGTGTTACCACGCCACGCCAAGCCCGGACCGACCATCTTAATGAATGATCAGGAGGTCGGTGTGCTAGATGCCAAAGAATTGGTTGACAAAGATGGGACAAATCTGGAGTTGACTCTCCTAAAGCTCAATCGCAATGAGAAGTTTAGGGATATCAGAGGGTTTCTGGCAAGAGAAGAAGCTGAGGTGAATGAGGCTGTTTTGGCAATAAACACAAGCAAGTTCCCCAACATGTACATACCCGTAGGTCAAGTCACCGACTACGGTTTTCTGAACTTGGGAGGAACGCCCACAAAGAGGATGCTCATGTACAATTTCCCAACTAGAGCAGGCCAATGTGGCGGTGTCCTCATGTCAACAGGGAAGGTTCTAGGAATACATGTAGGCGGAAATGGACACCAAGGATTCTCTGCTGCCCTCCTTAGACATTACTTCAATGAGGAACAAGGTGAGATAGAATTCATTGAGAGCTCAAAGGACGCAGGCTTCCCTGTGATCAACACCCCCAGCAAAACCAAGCTGGAACCAAGCGTGTTTCACCAGGTGTTTGAGGGCAACAAAGAGCCGGCAGTGCTTAGAAATGGGGATCCACGACTCAAGGTCAACTTTGAGGAGGCAATCTTCTCCAAGTACATTGGCAATGTTAACACCCACGTGGACGAATACATGCAAGAGGCCGTGGACCATTATGCAGGGCAGCTAGCTACACTGGACATCAGCACAGAGCCCATGAAACTGGAGGATGCCGTGTATGGTACAGAGGGGCTGGAAGCACTAGACCTAACCACCAGTGCAGGCTATCCGTACGTGGCCCTAGGTATCAAGAAAAGAGACATTCTCTCTAAGAAGACCAAAGACCTTACCAAGTTGAAGGAATGCATGGACAAGTATGGCCTAAACTTACCAATGGTAACTTACGTCAAAGATGAATTAAGATCTGCCGAGAAGGTAGCCAAGGGAAAGTCCAGACTTATTGAGGCCTCCAGTCTCAATGACTCAGTAGCAATGAGGCAAACATTTGGAAACCTGTACAAAACCTTTCATCTCAATCCGGGCATTGTCACGGGCAGTGCTGTTGGGTGTGACCCAGATGTATTTTGGAGTAAGATCCCTGTCATGCTTGATGGACATCTCATAGCTTTTGACTATTCAGGTTATGACGCCAGTCTCAGCCCGGTGTGGTTTGCATGTCTGAAACTCCTCCTAGAGAAACTAGGGTATACGAATAAGGAAACAAACTACATAGATTACCTCTGCAACTCTCACCACTTATATAGGGACAAGCACTACTTTGTGAGAGGCGGTATGCCATCAGGATGTTCGGGCACTAGCATATTTAATTCCATGATTAACAACATTATAATCAGGACTCTCATGCTGAAAGTTTATAAAGGCATTGATTTGGACCAATTCAGAATGATCGCTTATGGGGATGATGTGATTGCCTCCTACCCGTGGCCCATCGATGCGTCACTGTTAGCTGAAGCAGGAAAAGATTATGGATTGATCATGACCCCAGCAGACAAAGGTGAGTGCTTTAATGAGGTAACCTGGACAAATGTGACCTTTTTGAAAAGGTACTTCAGAGCAGATGAACAGTACCCATTCCTGGTCCATCCTGTTATGCCAATGAAGGACATACATGAGTCCATTAGATGGACTAAAGACCCCAAAAACACACAGGATCACGTGCGCTCGCTGTGCCTATTGGCTTGGCACAACGGGGAGCACGAATATGAGGAGTTTATTCGCAAGATCAGAAGCGTGCCCGTCGGGCGCTGCTTGACCCTTCCTGCATTTTCGACACTGCGTAGGAAGTGGTTGGACTCCTTCTAAAATTAGAGCACAATTAGTAGATTACAATTGGCTTAACCCTACCGCATGAACCGAACTTGATAAAAGTGCGGTAGGGGTAAATTCTCCGCATTCGGTGCGG |
| >3E1V_Barcode06_EV1_consensus | TTTGTGCGCCTGTTTTATACTCCCCCCCCTAAGGCAACTTTAGAAGCAAAGCAATTGTGATCAATAGTGGGTATGGCACACCAGTCATATCTTGATCAAGCACTTCTGTTCCCCCGGACTTAGTACCAATAGACTGCTCAAGCGGTTGAAGGGGAAAACGTTCGTTATCCGGCCAACTACTTCGAGAAACCTAGTAGCACCATGAAAGTTGCGGAGTGTTTCGCTCAGCACTTCCCCCGTGTAGATCAGGCTGATGAGTCACCGTATTCCCCACGGGCGACCGTGACGGTGGCTGCGTTGGCGGCCTGCCCATGGGGTAACCCATGGGACGCTCTAAAACAGACACGGTGCGAAGAGTCTATTGAGCTAGTTGGTAGTCCTCCGGCCCCTGAATGCGGCCAATCCTAACTGCGGAGCACATACTCCCAATCCAGGGAGCAGTGTGTCGTAATGGGTAACTCTGCAGCGGAACCGACTACTTTGGGTGTCCGTGTTTCCTTTTATTCTCACATTGACTGCTTATGGTGACAATTGAAAGATTGTTACCATATAGCTATTGGATTGGTCATCCGGTGAGCAATAGAGCTATTGTTTATCAATTTGTTGGATTTGTACCACTCAACTTTTCTGTTTTGAGAACACTCAACTACATCTTACTGCTAAACACATCAAAATGGGAGCACAGGTATCAACACAGAAGACCGGGGCGCACGAGACTAGCTTGAGCGCTACTGGTAACTCCATAATACACTACACGAATATTAATTATTACAAAGATGCAGCCTCTAACTCTGCCAATAGACAAGATTTCACCCAAGACCCTGGTAAGTTTACTGAACCAGTGAAAGATGTCATGATAAAAACCCTGCCAGCGCTGAATTCTCCAACGGTTGAAGAGTGCGGGTACAGTGACAGGGTCAGGTCAATCACACTTGGGAACTCCACTATTACAACTCAAGAGTGTGCCAATGTGGTGGTGGGGTACGGTGAATGGCCTGAGTATCTGAGTGATAACGAGGCAACTGCTGAGGACCAACCAACGCAGCCGGACGTGGCCACTTGCCGTTTTTACACCCTAGACTCAGTCCAATGGGAGAATGGGTCACCAGGTTGGTGGTGGAAGTTTCCCGACGCTCTAAGGGATATGGGATTATTTGGCCAAAATATGTACTACCATTACTTAGGCAGAGCCGGGTATACCATCCACGTACAATGCAATGCTTCCAAGTTTCATCAAGGCTGTATCCTGGTAGTGTGTGTCCCTGAGGCGGAGATGGGAAGTGCCCAAACCTCAGGGGTGGTCAACTACGAACACATTAGTAAGGGTGAGATCGCATCAAGGTTCACTACCACGACAACAGCAGAAGACCATGGCGTGCAGGCCGCGGTATGGAATGCTGGTATGGGCGTTGGAGTTGGGAACTTGACGATCTTCCCGCACCAATGGATCAACCTTCGCACCAACAACAGCGCCACAATTGTTATGCCATACGTAAATAGTGTACCAATGGACAATATGTATAGACATCACAACTTTACACTAATGATAATACCCTTTGTGCCTCTGGATTTCAGCGCGGGTGCATCCACATACGTGCCCATAACGGTGACAGTGGCCCCCATGTGTGCCGAGTACAATGGACTACGACTAGCTGGACACCAAGGACTACCGACCATGAACACCCCTGGCAGCAACCAATTTCTTACATCGGACGATTTCCAATCCCCGTCAGCGATGCCTCAATTTGATGTAACTCCAGAAATGCACATCCCTGGTGAGGTGCGCAACCTCATGGAAATTGCCGAAGTTGATTCTGTAATGCCAATTAACAATGATAGCGCCGCAAAAGTTTCATCCATGGAGGCTTATAGAGTCGAATTGAGCACCAACACTAATGCCGGGACTCAAGTGTTTGGCTTTCAACTGAACCCCGGAGCGGAATCAGTAATGAACCGCACATTAATGGGTGAAATCCTAAATTACTACGCACACTGGTCAGGAAGCATAAAGATAACATTCGTGTTCTGTGGTTCTGCCATGACCACTGGCAAGTTTCTGCTGTCTTACGCCCCACCAGGTGCAGGTGCGCCAAAAACTCGCAAGGATGCCATGTTAGGCACTCATGTGGTGTGGGATGTTGGGCTCCAATCCAGCTGCGTGTTATGCATCCCCTGGATTAGTCAAACCCATTACAGATTTGTGGAAAAGGATCCATACACCAATGCCGGGTTTGTGACATGTTGGTATCAGACCAGTGTAGTGTCCCCAGCGAGCAACCAGCCAAAGTGTTATATGATGTGCATGGTTTCTGCGTGTAATGACTTCTCAGTTCGCATGTTGAGAGATACCAAGTTCATTGAGCAAACATCTTTTTACCAAGGTGATGTGCAGAATGCTGTCGAAGGGGCTATGGTCAGGGTGGCAGATACAGTGCAAACTTCAGCCACAAACTCAGAGAGGGTGCCTAACTTGACAGCAGTAGAAACTGGTCACACTTCGCAGGTAGTACCTGGTGATACCATGCAGACTAGACATGTGATCAACAATCACGTGAGGTCAGAATCTACAATTGAGAACTTCCTTGCCAGATCAGCGTGTGTTTTCTTCCTAGAGTACAAGACAGGGACCAAAGAGGATTCCAATAGCTTCAACAATTGGGTGATTACAACCAGGCGAGTGGCTCAACTACGTAGAAAACTGGAAATGTTTACTTACCTACGGTTTGACATGGAAATCACCGTGGTCATTACAAGCTCGCAAGATCAGTCTACATCACAAAACCAGAATGCACCAGTGCTAACACACCAGATAATGTATGTACCACCAGGGGGACCCATACCCATAAGCGTGGATGATTACAGCTGGCAAACATCCACCAACCCCAGTATCTTTTGGACCGAAGGGAACGCTCCGGCACGCATGTCAATTCCATTTATTAGCATAGGCAATGCGTATAGTAATTTCTACGATGGGTGGTCTCACTTCTCCCAGAATGGCGTGTATGGCTTCACTACTCTGAACAACATGGGTCAATTGTTCTTCCGGCACGTAAACAAGCCCAACCCAGCCGCTATTACAAGTGTGGCGCGCATTTACTTCAAACCGAAACATGTACGCGCTTGGGTGCCTAGACCACCGCGCTTGTGTCCATACATCAATAGCACGAATGTCAACTTTGAACCCAAGCCAGTGACTGAAGTACGTACCAACATAATAACAACGGGTGCCTTTGGGCAGCAATCTGGCGCAGTGTACGTGGGCAACTACAGAGTGGTCAATAGGCACTTGGCGACTCACATTGATTGGCAAAACTGTGTGTGGGAGGACTATAACAGGGATCTACTGGTCAGCACAACTACAGCTCATGGGTGCGACACCATAGCTAGGTGCCAGTGCACGACAGGGGTGTACTTCTGCCTGAGCAGAAACAAACACTACCCAGTGTCATTTGAAGGTCCAGGATTGGTTGAGGTTCAAGAGAGTGAGTATTACCCAAGAAGGTACCAATCCCACGTGCTTCTTGCAGCCGGATTTTCTGAACCTGGAGATTGTGGTGGTATCTTGAGGTGTGAGCATGGTGTTATTGGTATAGTGACCATGGGAGGTGAAGGTGTCGTTGGTTTCGCCGATGTGCGAGACCTTCTATGGCTAGAGGATGACGCCATGGAGCAGGGAGTCAAGGACTACGTGGAACAGCTCGGCAACGCCTTTGGTTCAGGTTTCACCAATCAGATTTGTGAGCAGGTCAATCTCCTGAAAGAGTCCTTGGTAGGTCAAGACTCCATCTTGGAAAAGTCTTTAAAAGCACTAGTAAAAATCATATCAGCATTAGTGATCGTGGTAAGGAACCACGACGACTTGATCACAGTGACTGCTACACTAGCCCTCATTGGCTGCACCTCTTCACCATGGCGATGGCTCAAGCAGAAGGTATCACAATATTATGGAATACCCATGGCCGAGCGTCAGAACAATGGATGGCTCAAGAAATTCACTGAGATGACTAACGCCTGCAAAGGCATGGAGTGGATTGCCATTAAAATTCAGAAATTTATTGAATGGCTGAAAGTTAAGATTCTACCTGAAGTAAAAGAAAAACATGAATTTCTCAATAGATTAAAACAGCTGCCACTTCTTGAAAGTCAGATTGCTACCATAGAACAGAGCGCACCATCACAAGGTGACCAAGAACAGCTCTTCTCCAATGTGCAGTATTTTGCCCACTATTGCAGAAAGTACGCACCTCTGTATGCCGCCGAAGCAAAAAGAGTGTTCTCGTTGGAGAAAAAGATGAGCAACTACATACAGTTCAAGTCCAAATGCCGTATTGAGCCTGTATGTTTACTTCTCCATGGCAGCCCAGGAGCGGGGAAATCCGTGGCTACAAACCTAATTGGTAGATCCCTCGCGGAGAAACTTAACAGCTCTGTGTACTCGTTACCACCAGACCCAGATCATTTTGATGGATACAAACAACAAGCCGTAGTGATCATGGATGACCTGTGCCAGAATCCAGATGGGAAGGATGTGTCACTATTCTGTCAAATGGTATCCAGCGTGGACTTCGTACCACCCATGGCAGCTCTGGAGGAGAAAGGGATTCTTTTCACGTCCCCGTTTGTGCTAGCATCAACCAATGCGGGGTCTATCAATGCACCCACTGTGTCTGACAGCAGGGCACTTGCCAGAAGGTTCCACTTTGATATGAACATCGAGGTGATCTCCATGTATAGCCAGAATGGGAAGATTAACATGCCCATGTCTGTCAAAACATGTGATGAGGATTGCTGCCCGGTCAATTTCAAGAAATGCTGCCCGCTGGTGTGTGGTAAGGCCATTCAATTTATTGACAGAAAGACCCAAGTTAGGTATTCACTGGACATGTTGGTCACCGAGATGCTCAGGGAGTACAACCACAGACACAGCGTGGGTGCCACCCTCGAGGCTTTGTTCCAAGGGCCACCGGTCTACAGGGAGATTAAGATCAGTGTCGCTCCAGAAACACCCCCTCCACCAGCAATCGCTGACCTGCTAAAATCAGTAGACAGTGAGGCAGTAAGGGAGTACTGCAAGGAAAAAGGCTGGCTTGTGCCGGAAATTAGCTCCACCCTACAGATTGAGAAGTACATCAGTAGAGCATTTATCTGCCTACAGGCTCTGACTACATTTGTCTCAGTAGCTGGCATAATCTACATTATCTACAAATTGTTTGCCGGTTTTCAGGGCGCGTATACGGGGATGCCAAATCAGAAACCCAAGGTGCCCACTCTGAGACAGGCTAAGGTGCAGGGCCCGGCATTCGAGTTCGCCGTGGCGATGATGAAGAGAAACGCCAGCACAGTGAAAACAGAATATGGTGAGTTCACCATGCTCGGCATCTATGACAGATGGGCAGTGTTACCACGCCACGCCAAGCCCGGACCGACCATCTTAATGAATGATCAGGAGGTCGGTGTGCTAGATGCCAAAGAATTGGTTGACAAAGATGGGACAAATCTGGAGTTGACTCTCCTAAAGCTCAATCGCAATGAGAAGTTTAGGGATATCAGAGGGTTTCTGGCAAGAGAAGAAGCTGAGGTGAATGAGGCTGTTTTGGCAATAAACACAAGCAAGTTCCCCAACATGTACATACCCGTAGGTCAAGTCACCGACTACGGTTTTCTGAACTTGGGAGGAACGCCCACAAAGAGGATGCTCATGTACAATTTCCCAACTAGAGCAGGCCAATGTGGCGGTGTCCTCATGTCAACAGGGAAGGTTCTAGGAATACATGTAGGCGGAAATGGACACCAAGGATTCTCTGCTGCCCTCCTTAGACATTACTTCAATGAGGAACAAGGTGAGATAGAATTCATTGAGAGCTCAAAGGACGCAGGCTTCCCTGTGATCAACACCCCCAGCAAAACCAAGCTGGAACCAAGCGTGTTTCACCAGGTGTTTGAGGGCAACAAAGAGCCGGCAGTGCTTAGAAATGGGGATCCACGACTCAAGGTCAACTTTGAGGAGGCAATCTTCTCCAAGTACATTGGCAATGTTAACACCCACGTGGACGAATACATGCAAGAGGCCGTGGACCATTATGCAGGGCAGCTAGCTACACTGGACATCAGCACAGAGCCCATGAAACTGGAGGATGCCGTGTATGGTACAGAGGGGCTGGAAGCACTAGACCTAACCACCAGTGCAGGCTATCCGTACGTGGCCCTAGGTATCAAGAAAAGAGACATTCTCTCTAAGAAGACCAAAGACCTTACCAAGTTGAAGGAATGCATGGACAAGTATGGCCTAAACTTACCAATGGTAACTTACGTCAAAGATGAATTAAGATCTGCCGAGAAGGTAGCCAAGGGAAAGTCCAGACTTATTGAGGCCTCCAGTCTCAATGACTCAGTAGCAATGAGGCAAACATTTGGAAACCTGTACAAAACCTTTCATCTCAATCCGGGCATTGTCACGGGCAGTGCTGTTGGGTGTGACCCAGATGTATTTTGGAGTAAGATCCCTGTCATGCTTGATGGACATCTCATAGCTTTTGACTATTCAGGTTATGACGCCAGTCTCAGCCCGGTGTGGTTTGCATGTCTGAAACTCCTCCTAGAGAAACTAGGGTATACGAATAAGGAAACAAACTACATAGATTACCTCTGCAACTCTCACCACTTATATAGGGACAAGCACTACTTTGTGAGAGGCGGTATGCCATCAGGATGTTCGGGCACTAGCATATTTAATTCCATGATTAACAACATTATAATCAGGACTCTCATGCTGAAAGTTTATAAAGGCATTGATTTGGACCAATTCAGAATGATCGCTTATGGGGATGATGTGATTGCCTCCTACCCGTGGCCCATCGATGCGTCACTGTTAGCTGAAGCAGGAAAAGATTATGGATTGATCATGACCCCAGCAGACAAAGGTGAGTGCTTTAATGAGGTAACCTGGACAAATGTGACCTTTTTGAAAAGGTACTTCAGAGCAGATGAACAGTACCCATTCCTGGTCCATCCTGTTATGCCAATGAAGGACATACATGAGTCCATTAGATGGACTAAAGACCCCAAAAACACACAGGATCACGTGCGCTCGCTGTGCCTATTGGCTTGGCACAACGGGGAGCACGAATATGAGGAGTTTATTCGCAAGATCAGAAGCGTGCCCGTCGGGCGCTGCTTGACCCTTCCTGCATTTTCGACACTGCGTAGGAAGTGGTTGGACTCCTTCTAAAATTAGAGCACAATTAGTAGATTACAATTGGCTTAACCCTACCGCATGAACCGAACTTGATAAAAGTGCGGTAGGGGTAAATTCTCCGCATTCGGTGCGG |
| >1CVB5wt_Barcode09_consensus | AGGACGCTTCAATACAGACATGGTGCGAAGAGTCGATTGAGCTAGTTAGTAGTCCTCCGGCCCCTGAATGCGGCTGATCCCAACTGCGGAGCACATGCCCTCAATCCAGGGGGTGGTGTGTCGTAACGGGCAACTCTGCAGCGGAACCGACTACTTTGGGTGTCCGTGTTTCCTTTTATTCTTATACTGGCTGCTTATGGTGACAATTGAAAGGTTGTTGCCATATAGCTATTGGATTGGCCATCCAGTGTCTAATAGAGCTATTGTTTATCTTTTTGTTGGATTCATACCACTCAACTTAGGAGAAGTTAAAACATTACAACTCATTATACAACTGAATACAGCAAGATGGGAGCTCAGGTTTCAACGCAGAAAACTGGAGCGCACGAGACCGGGGTGAATGCTAGCGGTAATTCCATTATTCATTACACGAACATTAACTACTATAAAGATGCTGCGTCTAATTCAGCTAACCGGCAAGAATTTGCACAGGACCCAGGTAAGTTCACGGAGCCAGTAAAGGATATCATGATTAAATCAATGCCAGCCCTCAATTCGCCATCTGCCGAGGAGTGTGGGTACAGTGACAGGGTAAGATCCATCACGTTGGGAAACTCGACAATCACCACACAGGAGTGTGCTAATGTGGTGGTCGGGTATGGCACATGGCCCACGTATCTTAAAGATGAGGAAGCCACGGCAGAGGACCAACCAACTCAACCAGATGTGGCAACATGTAGGTTCTATACCTTAGAGTCAGTGATGTGGCAGCAGAGCTCACCTGGATGGTGGTGGAAATTCCCGGACGCCCTTTCGAACATGGGCTTGTTCGGGCAAAACATGCAGTACCACTATTTAGGGAGAGCTGGATACACAGTGCACGTTCAGTGCAACGCCTCCAAGTTCCACCAAGGATGCTTGTTGGTAGTGTGTGTGCCAGAGGCTGAGATGGGCTGTGCGACCATAGCCAATAAGCCAGACCAGAAGAGCCTGAGTAATGGAGAAACAGCCAACATGTTCGACTCACAAAATACGACCGGGCAAACAGCCGTGCAGGCCAATGTTATTAACGCAGGTATGGGGGTGGGTGTTGGTAACCTGACCATTTTCCCACACCAATGGATTAACCTGCGAACCAACAACAGTGCTACAATTGTTATGCCGTATATAAATAGTGTGCCAATGGATAATATGTTCAGACACAATAACTTCACCCTAATGATTATACCTTTTGCACCCTTGAGTTATAGCACGGGCGCAACCACTTACGTGCCTATCACGGTGACTGTGGCACCAATGTGCGCAGAATACAATGGTTTGCGATTGGCAGGGAAACAAGGGTTACCCACAATGCTAACACCTGGTAGCAACCAATTTTTGACCTCAGATGACTTCCAGTCGCCTTCCGCCATGCCACAGTTCGACGTGACGCCAGAAATGGCAATTCCTGGGCAGGTGAATAACTTGATGGAAATTGCAGAAGTTGATTCTGTGGTTCCAGTTAACAACACTGAGGGCAAAGTGACATCAATTGAAGCGTACCAAATACCTGTGCAATCAAACTCAACCAATGGCTCACAGGTGTTTGGGTTTCCTCTCATCCCTGGCGCCAGTAGTGTGCTGAATAGAACACTCCTGGGTGAGATCCTTAATTATTACACACATTGGTCAGGAAGCATTAAATTGACATTCATGTTTTGCGGGTCTGCGATGGCTACTGGTAAGTTTCTACTGGCTTATTCACCCCCTGGTGCTGGTGCACCAACAACCAGGAAAGAAGCAATGCTTGGAACACACGTCATCTGGGACGTTGGGTTGCAGTCCAGCTGTGTGTTATGCATCCCATGGATCAGTCAAACACACTACAGATACGTGGTGGTGGATGAGTATACCGCTGGAGGGTACATTACTTGTTGGTATCAGACAAACATCGTGGTACCTGCTGATACACAAAGTGATTGCAAAATTTTGTGTTTTGTATCAGCATGCAACGACTTTTCTGTGAGGATGTTAAAGGACACACCTTTTATAAAGCAGGACAACTTTTTCCAAGGACCACCCGGAGAAGCTGTCGAACGAGCGATTGCTCGCGTGGCTGATACCATAAGCAGTGGCCCTGTTAATTCAGAGTCAATTCCAGCACTAACAGCAGCAGAAACCGGGCACACATCCCAGGTGGTTCCGGCGGACACCATGCAAACCAGGCACGTAAAAAATTACCACTCGCGGTCTGAATCCACAGTAGAGAACTTTCTATGTAGATCCGCGTGTGTGTATTACACAACCTACAAGAACCATGGCACTGATGGAGACAACTTCGCCTACTGGGTGATCAACACAAGACAGGTTGCGCAATTGCGCCGCAAATTAGAAATGTTCACGTATGCCAGATTTGACTTAGAACTCACGTTTGTGATAACAAGCACACAAGAACAATCCACTATCAAAGGTCAAGACTCGCCTGTGTTAACTCATCAAATAATGTACGTGCCCCCAGGAGGCCCAGTGCCCACTAAGATTAACAGCTACAGCTGGCAAACATCAACCAACCCAAGTGTGTTTTGGACAGAGGGGAGTGCTCCTCCACGCATATCTATACCATTCATTAGTATTGGGAATGCTTACAGTATGTTTTATGACGGGTGGGCTAGGTTTGACAAGCAAGGGACTTATGGCATAAATACCTTGAACAACATGGGCACTTTGTACATGCGTCACGTAAACGACGGGAGTCCAGGTCCTATCGTGAGCACTGTGCGCATTTACTTTAAACCCAAACATGTGAAAACGTGGGTTCCCAGACCACCAAGGCTATGTCAGTATCAGAAGGCAGGAAACGTCAACTTTGAGCCAACAGGCGTGACAGAGTCCCGTACTGAAATCACGGCAATGCAAACCACTGGTCCATGTGGGCAACAATCTGGAGCTGTGTATGTGGGCAATTACAGGATCATAAACAGACACCTGGCAACAAGCCACGATTGGCAAAATTGCGTGTGGGAAGAATATAACAGAGATCTCCTGGTTAGCACAACCACAGCTCATGGTTGTGATATTATAGCACGGTGCCAATGTACAACTGGTGTGTACTTTTGTGCATCTAGAAATAAGCACTACCCAGTGAATTTCGAGGGTCCAGGTCTAGTGAAAGTTCAAGAGAGCGAGTACTACCCCGAGAGGTACCAGTCGCATGTACTTCTTGCAGCTGGATTCTCAGAGCCGGGTGATTGTGGAGGCATCTTGAGGTGTGAACATGGAGTTATTGGGCTCGTTACCATGGGTGGTGAGGGTGTAGTAGGCTTCGCTGACGTGCGAGATCTCTTGTGGTTGGAGGACGATGCCATGGAGCAGGGTGTTAAAGATTATGTAGAACAACTGGGAAACGCATTTGGCTCGGGTTTTACTAACCAGATCTGTGAGCAAGTCAATCTCCTCAAGGAATCCCTGATAGGGCAAGATTCTATATTAGAAAAATCCCTAAAGGCCCTGGTTAAAATCATATCAGCCCTAGTGATAGTGGTCAGGAACCACGATGACCTGATCACAGTGACTGCTACACTAGCTTTAATCGGTTGCACAACATCTCCATGGCGATGGCTCAAACAAAAAGTGAGTCAATACTACGGAATCCCCATGGCTGAAAGACAGAATAACGGTTGGTTAAAGAAATTCACCGAAATGACTAACGCTTGCAAGGGCATGGAGTGGATTGCTGTCAAAATTCAAAAGTTTATCGAGTGGCTCAAAATAAAGGTTCTGCCTGAAGTGAAGGAGAAGCATGAATTCCTCATCAGACTAAAGCAATTGCCACTCCTTGAAAGCCAAATTGCTACCATTGAGCAAAGTGCACCCTCACAAAGTGATCAAGAGCAGCTTTTTTCGAATGTCCAATACTTTGCCCATTACTGCAGGAAGTATGCACCATTGTACGCTGCCGAAGCAAAAAGAGTGTTTTCACTAGAAAAGAAAATGAGTAATTACATACAGTTCAAGTCCAAATGCCGTATTGAGCCTGTATGCTTACTCCTTCACGGTAGTCCAGGGGCTGGTAAGTCAGTTGCCACCAACTTGATAGGGAGGTCTCTTGCAGAGAAGCTCAACAGCTCTGTGTACTCTTTACCACCAGATCCAGATCATTTTGATGGGTACAAACAGCAAGCAGTAGTGATCATGGATGACCTCTGTCAAAACCCAGATGGGAAAGACGTGTCCCTGTTTTGCCAAATGGTGTCTAGTGTAGATTTTGTGCCACCAATGGCGGCGTTGGAAGAAAAAGGCATTCTCTTCACGTCCCCGTTCGTGTTAGCATCAACCAACGCGGGATCCATTAATGCACCAACAGTATCAGATAGCAGAGCACTTGCCAGAAGATTCCACTTTGACATGAATATTGAGGTGATTTCGATGTACAGCCAAAATGGCAAAATTAACATGCCCATGTCTGTTAAGACATGTGATGACGAGTGCTGCCCGATCAATTTCAAAAGGTGTTGCCCGTTAGTGTGTGGCAAGGCCATCCAGTTCATTGACAGGAGAACCCAAGTTAGATACTCACTGGATATGCTGGTTACTGAAATGTTTAGAGAGTACAATCACAGACACAGTGTGGGTACTACCCTCGAAGCTCTGTTCCAAGGACCACCAATATACAGGGAAATCAAGATCAGTGTGGCCCCGGAAACGCCCCCTCCACCAGCAATTGCTGACCTCCTAAAATCTGTAGATAGCGAGGCAGTAAGGGAGTACTGTAAGGGAAAAGGTTGGCTTATACCAGAAATCAACTCTACTTTACAAATTGAAAAACATGTCAGCAGGGCATTTATCTGCCTGCAAGCGCTGACCACATTTGTTTCAGTGGCTGGCATAATCTACATTATTTATAAGTTATTTGCCGGCTTTCAGGGTGCGTATACCGGGATGCCCAACCAGAAACCCAAGGTGCCCACCTTAAGACAGGCTAAGGTGCAAGGTCCAGCATTTGAATTTGCCGTAGCCATGATGAAGAGGAATGCCAGTACGGTTAAGACAGAATACGGTGAGTTCACAATGCTTGGCATCTATGATAGGTGGGCGGTGCTGCCGCGCCATGCCAAACCTGGGCCAACCATTTTAATGAATGAACAGGAGGTCGGTGTGCTAGATGCCAAAGAGCTGGTCGACAAAGATGGAACTAACCTGGAGCTGACGCTATTGAAACTCAACCGCAATGAAAAGTTCAGAGACATCAGAGGTTTCCTTGCAAGGGAAGAAGTTGAGGTGAACGAGGCAGTCTTGGCAATAAACACAAGCAAGTTCCCAAACATGTACATCCCTGTAGGTCAAGTCACTGACTACGGGTTTCTGAACCTAGGTGGTACCCCAACAAAGAGAATGCTCATGTATAACTTCCCTACCAGAGCAGGGCAGTGTGGCGGTGTCCTCATGTCAACAGGAAAGGTCCTTGGCATTCATGTGGGAGGAAATGGCCACCAGGGATTCTCTGCAGCTCTCCTCAAACACTATTTCAATGAAGAGCAGGGCGAAATAGAGTTTATTGAGAGTTCCAAAGATGCAGGCTTCCCTGTAATTAACACCCCTAGCAAAACTAAGCTGGAACCAAGTGTGTTTCACCAGATATTTGAGGGCAGCAAAGAACCAGCAGTTCTAAGAAACGGAGACCCGCGGCTCAAAGCCAACTTTGAAGAGGCAATCTTTTCCAAGTACATCGGAAATGTAAACACGCATGTGGACGGGTACATGATGGAAGCTGTTGATCACTACGCTGGGCAGCTGGCGACTTTGGATATCAACACAGAACCTATGAAGCTTGAGGATGCCGTCTATGGTACTGAAGGATTGGAAGCATTGGACTTGACCACTAGTGCAGGATACCCTTATGTTGCCCTGGGTATCAAGAAAAGGGACATCCTTTCCAAGAAGACCAAAGATCTTACCAAGTTGAAGGAGTGCATGGACAAGTATGGCCTGAATTTACCGATGGTGACCTATGTGAAGGATGAACTCAGGTCTGCTGAGAAGGTGGCTAAGGGAAAATCCAGGCTCATTGAGGCTTCTAGTCTCAACGACTCAGTGGCCATGAGGCAAACTTTTGGAAACCTGTACAAGACTTTCCACCTCAATCCAGGCATCGTCACCGGCAGTGCTGTCGGGTGTGATCCAGATGTATTCTGGAGCAAAATACCTGTAATGCTCGATGGACACCTTATAGCTTTTGATTATTCCGGTTACGATGCTAGTTTAAGCCCAGTTTGGTTTGCCTGTTTGAAGCTCCTCCTAGAGAAGTTAGGGTATACTCAGAAGGAAACCAACTACATAGACTACCTCTGCAACTCCCATCACCTGTACAGGGATAAGCATTATTTCGTGAGAGGTGGCATGCCATCAGGGTGTTCTGGCACTAGCATATTCAACTCTATGATTAATAACATTATTATCAGGACTCTCATGTTGAAGGTCTACAAAGGCATTGACTTAGACCAGTTCAGAATGATTGCTTACGGGGACGATGTGATAGCCTCTTACCCATGGCCCATTGATGCTTCACTGCTTGCAGAAGCAGGAAAGGACTATGGTTTGATCATGACCCCAGCAGATAAAGGAGAGTGCTTTAATGAGGTGACCTGGACTAATGTGACCTTCTTAAAAAGGTACTTTAGGGCTGATGAGCAATACCCGTTTTTGGTACACCCTGTGATGCCTATGAAGGACATACATGAATCTATTAGATGGACCAAAGATCCCAAGAACACTCAAGATCACGTGCGCTCGTTGTGCCTATTGGCGTGGCACAATGGGGAGCACGAGTATGAGGAGTTTATCCGAAAGATCAGAAGCGTGCCCGTAGGGCGCTGCTTGACCCTTCCAGCGTTTTCAACGCTGCGTAGGAAATGGTTGGACTCATTCTAAAATTAGAGCACAATTAATGAATTATGATTGGCTTGACCCTACCGCATGAACCGAACTTGACAAAAGTGCGGTAGGGGTAAATTCTCCGCATTCGGTGCGGAAAAAAAAAAAAAAAAAAAAAAAAAAAAAAAAAAAAAAAAAAAAAAAAAAAAAA |
| >1CVB5V_Barcode10_consensus | AGGACGCTTCAATACAGACATGGTGCGAAGAGTCGATTGAGCTAGTTAGTAGTCCTCCGGCCCCTGAATGCGGCTAATCCCAACTGCGGAGCACATGCCCTCAATCCAGGGGGTGGTGTGTCGTAACGGGCAACTCTGCAGCGGAACCGACTACTTTGGGTGTCCGTGTTTCCTTTTATTCTTATACTGGCTGCTTATGGTGACAATTGAAAGGTTGTTGCCATATAGCTATTGGATTGGCCATCCAGTGTCTAATAGAGCTATTGTTTATCTTTTTGTTGGATTCATACCACTCAACTTAGGAGAAGTTAAAACATTACAACTCATTATACAACTGAATACAGCAAGATGGGAGCTCAGGTTTCAACGCAGAAAACTGGAGCGCACGAGACCGGGGTGAATGCTAGCGGTAATTCCATTATTCATTACACGAACATTAACTACTATAAAGATGCTGCGTCTAATTCAGCTAACCGGCAAGAATTTGCACAGGACCCAGGTAAGTTCACGGAGCCAGTAAAGGATATCATGATTAAATCAATGCCAGCCCTCAATTCGCCATCTGCCGAGGAGTGTGGGTACAGTGACAGGGTAAGATCCATCACGTTGGGAAACTCGACAATCACCACACAGGAGTGTGCTAATGTGGTGGTCGGGTATGGCACATGGCCCACGTATCTTAAAGATGAGGAAGCCACGGCAGAGGACCAACCAACTCAACCAGATGTGGCAACATGTAGGTTCTATACCTTAGAGTCAGTGATGTGGCAGCAGAGCTCACCTGGATGGTGGTGGAAATTCCCGGACGCCCTTTCGAACATGGGCTTGTTCGGGCAAAACATGCAGTACCACTATTTAGGGAGAGCTGGATACACAGTGCACGTTCAGTGCAACGCCTCCAAGTTCCACCAAGGATGCTTGTTGGTAGTGTGTGTGCCAGAGGCTGAGATGGGCTGTGCGACCATAGCCAATAAGCCAGACCAGAAGAGCCTGAGTAATGGAGAAACAGCCAACATGTTCGACTCACAAAATACGACCGGGCAAACAGCCGTGCAGGCCAATGTTATTAACGCAGGTATGGGGGTGGGTGTTGGTAACCTGACCATTTTCCCACACCAATGGATTAACCTGCGAACCAACAACAGTGCTACAATTGTTATGCCGTATATAAATAGTGTGCCAATGGATAATATGTTCAGACACAATAACTTCACCCTAATGATTATACCTTTTGCACCCTTGAGTTATAGCACGGGCGCAACCACTTACGTGCCTATCACGGTGACTGTGGCACCAATGTGCGCAGAATACAATGGTTTGCGATTGGCAGGGAAACAAGGGTTACCCACAATGCTAACACCTGGTAGCAACCAATTTTTGACCTCAGATGACTTCCAGTCGCCTTCCGCCATGCCACAGTTCGACGTGACGCCAGAAATGGCAATTCCTGGGCAGGTGAATAACTTGATGGAAATTGCAGAAGTTGATTCTGTGGTTCCAGTTAACAACACTGAGGGCAAAGTGACATCAATTGAAGCGTACCAAATACCTGTGCAATCAAACTCAACCAATGGCTCACAGGTGTTTGGGTTTCCTCTCATCCCTGGCGCCAGTAGTGTGCTGAATAGAACACTCCTGGGTGAGATCCTTAATTATTACACACATTGGTCAGGAAGCATTAAATTGACATTCATGTTTTGCGGGTCTGCGATGGCTACTGGTAAGTTTCTACTGGCTTATTCACCCCCTGGTGCTGGTGCACCAACAACCAGGAAAGAAGCAATGCTTGGAACACACGTCATCTGGGACGTTGGGTTGCAGTCCAGCTGTGTGTTATGCATCCCATGGATCAGTCAAACACACTACAGATACGTGGTGGTGGATGAGTATACCGCTGGAGGGTACATTACTTGTTGGTATCAGACAAACATCGTGGTACCTGCTGATACACAAAGTGATTGCAAAATTTTGTGTTTTGTATCAGCATGCAACGACTTTTCTGTGAGGATGTTAAAGGACACACCTTTTATAAAGCAGGACAACTTTTTCCAAGGACCACCCGGAGAAGCTGTCGAACGAGCGATTGCTCGCGTGGCTGATACCATAAGCAGTGGCCCTGTTAATTCAGAGTCAATTCCAGCACTAACAGCAGCAGAAACCGGGCACACATCCCAGGTGGTTCCGGCGGACACCATGCAAACCAGGCACGTAAAAAATTACCACTCGCGGTCTGAATCCACAGTAGAGAACTTTCTATGTAGATCCGCGTGTGTGTATTACACAACCTACAAGAACCATGGCACTGATGGAGACAACTTCGCCTACTGGGTGATCAACACAAGACAGGTTGCGCAATTGCGCCGCAAATTAGAAATGTTCACGTATGCCAGATTTGACTTAGAACTCACGTTTGTGATAACAAGCACACAAGAACAATCCACTATCCAAGGTCAAGACTCGCCTGTGTTAACTCATCAAATAATGTACGTGCCCCCAGGAGGCCCAGTGCCCACTAAGATTAACAGCTACAGCTGGCAAACATCAACCAACCCAAGTGTGTTTTGGACAGAGGGGAGTGCTCCTCCACGCATATCTATACCATTCATTAGTATTGGGAATGCTTACAGTATGTTTTATGACGGGTGGGCTAGGTTTGACAAGCAAGGGACTTATGGCATAAATACCTTGAACAACATGGGCACTTTGTACATGCGTCACGTAAACGACGGGAGTCCAGGTCCTATCGTGAGCACTGTGCGCATTTACTTTAAACCCAAACATGTGAAAACGTGGGTTCCCAGACCACCAAGGCTATGTCAGTATCAGAAGGCAGGAAACGTCAACTTTGAGCCAACAGGCGTGACAGAGTCCCGTACTGAAATCACGGCAATGCAAACCACTGGTCCATGTGGGCAACAATCTGGAGCTGTGTATGTGGGCAATTACAGGATCATAAACAGACACCTGGCAACAAGCCACGATTGGCAAAATTGCGTGTGGGAAGAATATAACAGAGATCTCCTGGTTAGCACAACCACAGCTCATGGTTGTGATATTATAGCACGGTGCCAATGTACAACTGGTGTGTACTTTTGTGCATCTAGAAATAAGCACTACCCAGTGAATTTCGAGGGTCCAGGTCTAGTGAAAGTTCAAGAGAGCGAGTACTACCCCGAGAGGTACCAGTCGCATGTACTTCTTGCAGCTGGATTCTCAGAGCCGGGTGATTGTGGAGGCATCTTGAGGTGTGAACATGGAGTTATTGGGCTCGTTACCATGGGTGGTGAGGGTGTAGTAGGCTTCGCTGACGTGCGAGATCTCTTGTGGTTGGAGGACGATGCCATGGAGCAGGGTGTTAAAGATTATGTAGAACAACTGGGAAACGCATTTGGCTCGGGTTTTACTAACCAGATCTGTGAGCAAGTCAATCTCCTCAAGGAATCCCTGATAGGGCAAGATTCTATATTAGAAAAATCCCTAAAGGCCCTGGTTAAAATCATATCAGCCCTAGTGATAGTGGTCAGGAACCACGATGACCTGATCACAGTGACTGCTACACTAGCTTTAATCGGTTGCACAACATCTCCATGGCGATGGCTCAAACAAAAAGTGAGTCAATACTACGGAATCCCCATGGCTGAAAGACAGAATAACGGTTGGTTAAAGAAATTCACCGAAATGACTAACGCTTGCAAGGGCATGGAGTGGATTGCTGTCAAAATTCAAAAGTTTATCGAGTGGCTCAAAATAAAGGTTCTGCCTGAAGTGAAGGAGAAGCATGAATTCCTCATCAGACTAAAGCAATTGCCACTCCTTGAAAGCCAAATTGCTACCATTGAGCAAAGTGCACCCTCACAAAGTGATCAAGAGCAGCTTTTTTCGAATGTCCAATACTTTGCCCATTACTGCAGGAAGTATGCACCATTGTACGCTGCCGAAGCAAAAAGAGTGTTTTCACTAGAAAAGAAAATGAGTAATTACATACAGTTCAAGTCCAAATGCCGTATTGAGCCTGTATGCTTACTCCTTCACGGTAGTCCAGGGGCTGGTAAGTCAGTTGCCACCAACTTGATAGGGAGATCTCTTGCAGAGAAGCTCAACAGCTCTGTGTACTCTTTACCACCAGATCCAGATCATTTTGATGGGTACAAACAGCAAGCAGTAGTGATCATGGATGACCTCTGTCAAAACCCAGATGGGAAAGACGTGTCCCTGTTTTGCCAAATGGTGTCTAGTGTAGATTTTGTGCCACCAATGGCGGCGTTGGAAGAAAAAGGCATTCTCTTCACGTCCCCGTTCGTGTTAGCATCAACCAACGCGGGATCCATTAATGCACCAACAGTATCAGATAGCAGAGCACTTGCCAGAAGATTCCACTTTGACATGAATATTGAGGTGATTTCGATGTACAGCCAAAATGGCAAAATTAACATGCCCATGTCTGTTAAGACATGTGATGACGAGTGCTGCCCGATCAATTTCAAAAGGTGTTGCCCGTTAGTGTGTGGCAAGGCCATCCAGTTCATTGACAGGAGAACCCAAGTTAGATACTCACTGGATATGCTGGTTACTGAAATGTTTAGAGAGTACAATCACAGACACAGTGTGGGTACTACCCTCGAAGCTCTGTTCCAAGGACCACCAATATACAGGGAAATCAAGATCAGTGTGGCCCCGGAAACGCCCCCTCCACCAGCAATTGCTGACCTCCTAAAATCTGTAGATAGCGAGGCAGTAAGGGAGTACTGTAAGGGAAAAGGTTGGCTTATACCAGAAATCAACTCTACTTTACAAATTGAAAAACATATCAGCAGGGCATTTATCTGCCTGCAAGCGCTGACCACATTTGTTTCAGTGGCTGGCATAATCTACATTATTTATAAGTTATTTGCCGGCTTTCAGGGTGCGTATACCGGGATGCCCAACCAGAAACCCAAGGTGCCCACCTTAAGACAGGCTAAGGTGCAAGGTCCAGCATTTGAATTTGCCGTAGCCATGATGAAGAGGAATGCCAGTACGGTTAAGACAGAATACGGTGAGTTCACAATGCTTGGCATCTATGATAGGTGGGCGGTGCTGCCGCGCCATGCCAAACCTGGGCCAACCATTTTAATGAATGAACAGGAGGTCGGTGTGCTAGATGCCAAAGAGCTGGTCGACAAAGATGGAACTAACCTGGAGCTGACGCTATTGAAACTCAACCGCAATGAAAAGTTCAGAGACATCAGAGGTTTCCTTGCAAGGGAAGAAGTTGAGGTGAACGAGGCAGTCTTGGCAATAAACACAAGCAAGTTCCCAAACATGTACATCCCTGTAGGTCAAGTCACTGACTACGGGTTTCTGAACCTAGGTGGTACCCCAACAAAGAGAATGCTCATGTATAACTTCCCTACCAGAGCAGGGCAGTGTGGCGGTGTCCTCATGTCAACAGGAAAGGTCCTTGGCATTCATGTGGGAGGAAATGGCCACCAGGGATTCTCTGCAGCTCTCCTCAAACACTATTTCAATGAAGAGCAGGGCGAAATAGAGTTTATTGAGAGTTCCAAAGATGCAGGCTTCCCTGTAATTAACACCCCTAGCAAAACTAAGCTGGAACCAAGTGTGTTTCACCAGATATTTGAGGGCAGCAAAGAACCAGCAGTTCTAAGAAACGGAGACCCGCGGCTCAAAGCCAACTTTGAAGAGGCAATCTTTTCCAAGTACATCGGAAATGTAAACACGCATGTGGACGGGTACATGATGGAAGCTGTTGATCACTACGCTGGGCAGCTGGCGACTTTGGATATCAACACAGAACCTATGAAGCTTGAGGATGCCGTCTATGGTACTGAAGGATTGGAAGCATTGGACTTGACCACTAGTGCAGGATACCCTTATGTTGCCCTGGGTATCAAGAAAAGGGACATCCTTTCCAAGAAGACCAAAGATCTTACCAAGTTGAAGGAGTGCATGGACAAGTATGGCCTGAATTTACCGATGGTGACCTATGTGAAGGATGAACTCAGGTCTGCTGAGAAGGTGGCTAAGGGAAAATCCAGGCTCATTGAGGCTTCTAGTCTCAACGACTCAGTGGCCATGAGGCAAACTTTTGGAAACCTGTACAAGACTTTCCACCTCAATCCAGGCATCGTCACCGGCAGTGCTGTCGGGTGTGATCCAGATGTATTCTGGAGCAAAATACCTGTAATGCTCGATGGACACCTTATAGCTTTTGATTATTCCGGTTACGATGCTAGTTTAAGCCCAGTTTGGTTTGCCTGTTTGAAGCTCCTCCTAGAGAAGTTAGGGTATACTCAGAAGGAAACCAACTACATAGACTACCTCTGCAACTCCCATCACCTGTACAGGGATAAGCATTATTTCGTGAGAGGTGGCATGCCATCAGGGTGTTCTGGCACTAGCATATTCAACTCTATGATTAATAACATTATTATCAGGACTCTCATGTTGAAGGTCTACAAAGGCATTGACTTAGACCAGTTCAGAATGATTGCTTACGGGGACGATGTGATAGCCTCTTACCCATGGCCCATTGATGCTTCACTGCTTGCAGAAGCAGGAAAGGACTATGGTTTGATCATGACCCCAGCAGATAAAGGAGAGTGCTTTAATGAGGTGACCTGGACTAATGTGACCTTCTTAAAAAGGTACTTTAGGGCTGATGAGCAATACCCGTTTTTGGTACACCCTGTGATGCCTATGAAGGACATACATGAATCTATTAGATGGACCAAAGATCCCAAGAACACTCAAGATCACGTGCGCTCGTTGTGCCTATTGGCGTGGCACAATGGGGAGCACGAGTATGAGGAGTTTATCCGAAAGATCAGAAGCGTGCCCGTAGGGCGCTGCTTGACCCTTCCAGCGTTTTCAACGCTGCGTAGGAAATGGTTGGACTCATTCTAAAATTAGAGCACAATTAATGAATTATGATTGGCTTGACCCTACCGCATGAACCGAACTTGACAAAAGTGCGGTAGGGGTAAATTCTCCGCATTCGGTGCGGAAAAAAAAAAAAAAAAAAAAAAAAAAAAAAAAAAAAAAAAAAAAAAAAAAAAAA |

**Table S5.** Mutations found in EV1 genomes after 6 passages of the virus in RD cells in the presence or absence of vemurafenib were determined

| **Alignment**  **_NT_Pos** | **Region** | **Region**  **Codon_Pos** | **Polyprotein**  **Codon_Pos** | **Ref**  **NT** | **Alt**  **NT** | **Ref**  **AA** | **Alt**  **AA** | **Type** |
| --- | --- | --- | --- | --- | --- | --- | --- | --- |
| 76 | 5UTR |  |  | G | A |  |  | noncoding |
| 2446 | VP1 | 132 | 700 | A | C | K | Q | nonsynonymous |
| 4080 | 2C | 144 | 1244 | G | A | R | R | synonymous |
| 4807 | 3A | 58 | 1487 | G | A | V | I | nonsynonymous |

**Table S6**. Survival outcomes for monotherapy, double and triple regimens. MST - mean survival time; % Mort - percent of mortality; PI - protection index.

| Group | Survival/Total | % Mort | PI | MST | DMST |
| --- | --- | --- | --- | --- | --- |
| Placebo | 0/41 | 100.0 | 0.0 | 4.4 |  |
| Pleconaril (P) 5 mg/kg | 0/16 | 100.0 | 0.0 | 5.4 | 1.0 |
| P 10 mg/kg | 0/20 | 100.0 | 0.0 | 5.1 | 0.7 |
| P 15 mg/kg | 2/18 | 88.9 | 11.1 | ˃6.2 | 1.8 |
| P 20 mg/kg | 2/23 | 91.3 | 8.7 | >6.7 | 2.3 |
| AG-7404 (A) 1 mg/kg | 2/40 | 95.0 | 5.0 | >6.4 | 2.0 |
| A 5mg/kg | 4/18 | 77.8 | 22.2 | ˃6.3 | 1.9 |
| Mindeudesivir (M) 5mg/kg | 4/33 | 87.9 | 12.1 | >6.5 | 2.1 |
| M 10 mg/kg | 1/22 | 95.5 | 4.5 | ˃4.4 | 0.0 |
| AM 1/ 5mg/kg | 0/15 | 100.0 | 0.0 | 4.7 | 0.3 |
| PA 20/1 mg/kg | 0/14 | 100.0 | 0.0 | 6.2 | 1.8 |
| PM 20/5 mg/kg | 0/14 | 100.0 | 0.0 | 5.6 | 0.8 |
| PAM 20/1/5 mg/kg | 11/46 | 76.1 | 23.9 | ˃7.9^^^ | 3.5 |
| PAM 20/5/5 mg/kg | 0/24 | 100.0 | 0.0 | 6.5 | 2.2 |
| PMA 20/5/1 mg/kg | 2/20 | 90.0 | 10.0 | ˃5.7 | 1.3 |
| PMA 20/5/5 mg/kg | 0/24 | 100.0 | 0.0 | 5.3 | 0.9 |
| MAP 5/1/20 mg/kg | 5/26 | 80.8 | 19.2 | ˃6.9 | 2.5 |
| APM 1/20/5 mg/kg | 0/26 | 100.0 | 0.0 | 5.5 | 1.1 |
| PAM_simult. 20/1/5 mg/kg | 0/24 | 100.0 | 0.0 | 5.0 | 0.6 |

Table S7. Antienterovirals.

| **Drug** | **Virus** | **Target** | **Cell cultures** | **Organoids** | **Animal model** | **Phase I** | **Phase II** | **Drugbank ID** | **References** |
| --- | --- | --- | --- | --- | --- | --- | --- | --- | --- |
| AG-7404 | EV1 | 3C protease |  | x |  |  |  | N/A | PMID: 39843710 |
| AG-7404 | EV6 | 3C protease |  | x |  |  |  | N/A | PMID: 39843710 |
| AG-7404 | EV7 | 3C protease | x |  |  |  |  | N/A | PMID: 39843710 |
| AG-7404 | EV11 | 3C protease |  | x |  |  |  | N/A | PMID: 39843710 |
| AG-7404 | CVA-13 | 3C protease | x |  |  |  |  | N/A | PMID: 39843710 |
| AG-7404 | CVB-2 | 3C protease | x |  |  |  |  | N/A | PMID: 15917520 |
| AG-7404 | CVB-3 | 3C protease | x |  |  |  |  | N/A | PMID: 15917520 |
| AG-7404 | CVB-5 | 3C protease |  | x |  |  |  | N/A | PMID: 39843710 |
| AG-7404 | PV-1 | 3C protease | x |  |  |  |  | N/A | PMID: 23499651 |
| ALD | EVA-71 | Capsid (VP1) | x |  |  |  |  | N/A | PMID: 24509833 |
| Anisomycin | EV1 | eEF1A1 | x |  |  |  |  | DB07374 | PMID:33080984 |
| Anisomycin | CVB-3 | eEF1A1 | x |  |  |  |  | DB07374 | PMID:37156267 |
| Betulinic acid | EV6 | Uknown | x |  |  |  |  | DB12480 | PMID:12845810 |
| Compund 17 | EV1 | Capsid | x |  |  |  |  | N/A | PMID: 31185007 |
| Compund 17 | EV7 | Capsid | x |  |  |  |  | N/A | PMID: 31185007 |
| Compund 17 | CVB-1 | Capsid | x |  |  |  |  | N/A | PMID: 31185007 |
| Compund 17 | CVB-3 | Capsid | x |  |  |  |  | N/A | PMID: 31185007 |
| Compund 17 | CVB-4 | Capsid | x |  |  |  |  | N/A | PMID: 31185007 |
| Compund 17 | CVB-5 | Capsid | x |  |  |  |  | N/A | PMID: 31185007 |
| Compund 17 | CVB-6 | Capsid | x |  |  |  |  | N/A | PMID: 31185007 |
| Cucurbit[7]uril | EV11 | Human polyamines | x |  |  |  |  | N/A | PMID: 34037947 |
| Cucurbit[7]uril | EVA-71 | Human polyamines | x |  |  |  |  | N/A | PMID: 34037947 |
| Cucurbit[7]uril | CVB-3 | Human polyamines | x |  |  |  |  | N/A | PMID: 34037947 |
| Cycloheximide | CVB-1 | Host translation | x |  |  |  |  | DB20401 | <https://doi.org/10.4167/jbv.2025.55.2.187> |
| Cycloheximide | CVB-2 | Host translation | x |  |  |  |  | DB20401 | <https://doi.org/10.4167/jbv.2025.55.2.187> |
| Cycloheximide | CVB-3 | Host translation | x |  |  |  |  | DB20401 | <https://doi.org/10.4167/jbv.2025.55.2.187> |
| Cycloheximide | CVB-4 | Host translation | x |  |  |  |  | DB20401 | <https://doi.org/10.4167/jbv.2025.55.2.187> |
| Cycloheximide | CVB-5 | Host translation | x |  |  |  |  | DB20401 | https://doi.org/10.4167/jbv.2025.55.2.187 |
| Cycloheximide | CVB-6 | Host translation | x |  |  |  |  | DB20401 | https://doi.org/10.4167/jbv.2025.55.2.187 |
| Cycloheximide | EV1 | Host translation | x |  |  |  |  | DB20401 | PMID: 33080984 |
| Cycloheximide | EV6 | Host translation | x |  |  |  |  | DB20401 | PMID: 38417531 |
| CypA-11 | EVA-71 | Cyclophilin A inhibitor | x |  |  |  |  | N/A | PMID: 26564266 |
| Dalbavacin | EV1 | Unkown | x |  |  |  |  | DB06219 | PMID:29698664 |
| Emetine | EV1 | IRES-driven translation | x |  |  |  |  | DB13393 | PMID:31635418 |
| Emetine | EV6 | IRES-driven translation | x |  |  |  |  | DB13393 | PMID: 31734270 |
| Emetine | EVA-71 | IRES-driven translation | |  | x |  |  | DB13393 | PMID: 31734270 |
| Emetine | CVB-1 | IRES-driven translation | x |  |  |  |  | DB13393 | PMID: 31734270 |
| Enviroxime | EVA-71 | 3A inhibitor | x |  |  |  |  | DB19577 | PMID: 25199773 |
| Enviroxime | EV1 | 3A inhibitor | x |  |  |  |  | DB19577 | PMID: 38417531 |
| Enviroxime | EV6 | 3A inhibitor | x |  |  |  |  | DB19577 | PMID: 31705692 |
| Enviroxime | CVB-1 | 3A inhibitor | x |  |  |  |  | DB19577 | PMID: 31705692 |
| Enviroxime | CVB-2 | 3A inhibitor | x |  |  |  |  | DB19577 | PMID: 31705692 |
| Enviroxime | CVB-3 | 3A inhibitor | x |  |  |  |  | DB19577 | PMID: 31705692 |
| Enviroxime | CVB-4 | 3A inhibitor | x |  |  |  |  | DB19577 | PMID: 31705692 |
| Enviroxime | CVB-5 | 3A inhibitor | x |  |  |  |  | DB19577 | PMID: 31705692 |
| Enviroxime | CVB-6 | 3A inhibitor | x |  |  |  |  | DB19577 | PMID: 31705692 |
| Favipiravir | EVA-71 | RdRp | x |  |  |  |  | DB12466 | PMID: 27353263 |
| Favipiravir | HAV | RdRp | x |  |  |  |  | DB12466 | PMID: 35269774 |
| Favipiravir | CVB-1 | RdRp | x |  |  |  |  | DB12466 | PMID: 31705692 |
| Favipiravir | CVB-2 | RdRp | x |  |  |  |  | DB12466 | PMID: 31705692 |
| Favipiravir | CVB-3 | RdRp | x |  |  |  |  | DB12466 | PMID: 31705692 |
| Favipiravir | CVB-5 | RdRp | x |  |  |  |  | DB12466 | PMID: 31705692 |
| Favipiravir | CVB-6 | RdRp | x |  |  |  |  | DB12466 | PMID: 31705692 |
| Fluoxetine | EV1 | 2C inhibitor | x |  |  |  |  | DB00472 | PMID:23335743 |
| Fluoxetine | EV11 | 2C inhibitor | x |  |  |  |  | DB00472 | PMID:23335743 |
| Fluoxetine | CVB-1 | 2C inhibitor | x |  |  |  |  | DB00472 | PMID: 31705692 |
| Fluoxetine | CVB-2 | 2C inhibitor | x |  |  |  |  | DB00472 | PMID: 31705692 |
| Fluoxetine | CVB-3 | 2C inhibitor | x |  |  |  |  | DB00472 | PMID: 31705692 |
| Fluoxetine | CVB-4 | 2C inhibitor |  |  | x |  |  | DB00472 | PMID: 31705692 |
| Fluoxetine | CVB-5 | 2C inhibitor | x |  |  |  |  | DB00472 | PMID: 31705692 |
| Fluoxetine | CVB-6 | 2C inhibitor | x |  |  |  |  | DB00472 | PMID: 31705692 |
| FNC | EV6 | 3Dpol | x |  |  |  |  | DB16407 | PMID: 38687016 |
| FNC | EVA-71 | 3Dpol |  |  | x |  |  | DB16407 | PMID: 32075935 |
| FNC | CVB-3 | 3Dpol | x |  |  |  |  | DB16407 | PMID: 32075935 |
| Gemcitabine | EV1 | 3Dpol | x |  |  |  |  | DB00441 | PMID: 29698664 |
| Gemcitabine | EVA-71 | 3Dpol | x |  |  |  |  | DB00441 | PMID: 26526589 |
| Gemcitabine | CVB-3 | 3Dpol | x |  |  |  |  | DB00441 | PMID: 26526589 |
| GS-9620 | EVA-71 | TLR7 |  |  | x |  |  |  | PMID: 29425831 |
|  |  |  |  |  |  |  |  | DB12687 |  |
|  |  |  |  |  |  |  |  | DB12687 |  |
|  |  |  |  |  |  |  |  | DB12687 |  |
|  |  |  |  |  |  |  |  | DB12687 |  |
|  |  |  |  |  |  |  |  | DB12687 |  |
|  |  |  |  |  |  |  |  | DB12687 |  |
|  |  |  |  |  |  |  |  | DB12687 |  |
| GSK583 | CVB-5 | Human RIPK2 | x |  |  |  |  | N/A | PMID:34001511 |
| GSK717 | CVB-5 | Human NOD2 | x |  |  |  |  | N/A | PMID:34001511 |
| Guanidine HCl | EV1 | 2C inhibitor | x |  |  |  |  | DB00536 | PMID: 13990297 |
| Guanidine HCl | EVA-71 | 2C inhibitor | x |  |  |  |  | DB00536 | PMID: 22814431 |
| Guanidine HCl | CVA-13 | 2C inhibitor | x |  |  |  |  | DB00536 | PMID: 13990297 |
| Guanidine HCl | CVB-1 | 2C inhibitor | x |  |  |  |  | DB00536 | PMID: 10757231 |
| Guanidine HCl | CVB-3 | 2C inhibitor | x |  |  |  |  | DB00536 | PMID: 10757231 |
| Guanidine HCl | CVB-5 | 2C inhibitor | x |  |  |  |  | DB00536 | PMID: 13990297 |
| Homoharringtonine | EV1 | Human ribosome | x |  |  |  |  | DB04865 | PMID:31635418 |
| HS83128 | HAV | RNA synthesis |  |  | x |  |  | N/A | PMID: 40118118 |
| IMP-1088 | EV1 | NMT1/NMT2 | x |  |  |  |  | N/A | PMID: 38417531 |
| IMP-1088 | EV6 | NMT1/NMT2 | x |  |  |  |  | N/A | PMID: 38417531 |
| Ionosine | EVA-71 | RdRp | x |  |  |  |  | DB13156 | PMID: 28146624 |
| Ionosine | HAV | RdRp | x |  |  |  |  | DB13156 | PMID:1279362 |
| Itraconazole | EVA-71 | 3A inhibitor | x |  |  |  |  | DB01167 | PMID: 27353263 |
| Itraconazole | CVB-1 | 3A inhibitor | x |  |  |  |  | DB01167 | PMID: 31705692 |
| Itraconazole | CVB-2 | 3A inhibitor | x |  |  |  |  | DB01167 | PMID: 31705692 |
| Itraconazole | CVB-3 | 3A inhibitor | x |  |  |  |  | DB01167 | PMID: 31705692 |
| Itraconazole | CVB-4 | 3A inhibitor | x |  |  |  |  | DB01167 | PMID: 31705692 |
| Itraconazole | CVB-5 | 3A inhibitor | x |  |  |  |  | DB01167 | PMID: 31705692 |
| Itraconazole | CVB-6 | 3A inhibitor | x |  |  |  |  | DB01167 | PMID: 31705692 |
| JX040 | EVA-71 | 2C ATPase | x |  |  |  |  | N/A | PMID: 29346733 |
| JX040 | CVB-3 | 2C ATPase | x |  |  |  |  | N/A | PMID: 29346733 |
| Loxapine | HAV | HAV 2C Protein | x |  |  |  |  | DB00408 | PMID: 38478584 |
| L-SP40 | EVA-71 | Nucleolin receptor | |  | x |  |  | N/A | PMID: 34715144 |
| Masitinib | HAV | IRES-activity | x |  |  |  |  | DB11526 | PMID: 37298659 |
| MDL-860 | EVA-71 | PI4KB | x |  |  |  |  | N/A | PMID: 21466823 |
| MDL-860 | PV-1 | PI4KIIIß | x |  |  |  |  | N/A | PMID: 30782563 |
| MDL-860 | CVA-9 | PI4KIIIß | x |  |  |  |  | N/A | <https://doi.org/10.3389/av.2024.12361> |
| MDL-860 | CVB-1 | PI4KIIIß | x |  |  |  |  | N/A | PMID: 30782563 |
| MDL-860 | CVB-2 | PI4KIIIß | x |  |  |  |  | N/A | PMID: 14695668 |
| MDL-860 | CVB-3 | PI4KIIIß |  |  | x |  |  | N/A | PMID: 14695668 |
| MDL-860 | CVB-4 | PI4KIIIß | x |  |  |  |  | N/A | <https://doi.org/10.3389/av.2024.12361> |
| MDL-860 | CVB-5 | PI4KIIIß | x |  |  |  |  | N/A | PMID: 14695668 |
| MDL-860 | CVB-6 | PI4KIIIß | x |  |  |  |  | N/A | PMID: 14695668 |
| Mindeudesivir | EV1 | RdRp |  | x |  |  |  | N/A | PMID: 39843710 |
| Mindeudesivir | EV6 | RdRp |  | x |  |  |  | N/A | PMID: 39843710 |
| Mindeudesivir | EV7 | RdRp |  |  |  |  |  | N/A | PMID: 39843710 |
| Mindeudesivir | EV11 | RdRp |  | x |  |  |  | N/A | PMID: 39843710 |
| Mindeudesivir | CVA-13 | RdRp | x |  |  |  |  | N/A | PMID: 39843710 |
| Mindeudesivir | CVB-5 | RdRp |  | x |  |  |  | N/A | PMID: 39843710 |
| Mindeudesivir | CVB-6 | RdRp | x |  |  |  |  | N/A | PMID: 39843710 |
| Navitoclax | EV6 | Human BCL2 | x |  |  |  |  | DB12340 | PMID: 23887633 |
| NITD008 | EVA-71 | 3Dpol |  |  | x |  |  | N/A | PMID: 25100827 |
| NK-1.9k | EVA-71 | 3C protease | x |  |  |  |  | N/A | PMID: 28063993 |
| Obatoclax | EV1 | Human MCL1 | x |  |  |  |  | DB12191 | PMID: 31635418 |
| OSW-1 | EVA-71 | OSBP | x |  |  |  |  | N/A | PMID: 25752737 |
| OSW-1 | CVA9 | OSBP | x |  |  |  |  | N/A | PMID: 30576108 |
| OSW-1 | CVB-3 | OSBP | x |  |  |  |  | N/A | PMID: 25752737 |
| Oxoglaucine | PV-1 | PI4KIIIß | x |  |  |  |  | N/A | PMID: 10757231 |
| Oxoglaucine | CVA-9 | PI4KIIIß | x |  |  |  |  | N/A | PMID: 10757231 |
| Oxoglaucine | CVB-1 | PI4KIIIß | x |  |  |  |  | N/A | PMID: 34978843 |
| Oxoglaucine | CVB-3 | PI4KIIIß | x |  |  |  |  | N/A | PMID: 34978843 |
| Oxoglaucine | CVB-4 | PI4KIIIß | x |  |  |  |  | N/A | PMID: 10757231 |
| Pirodavir | EV11 | Capsid (VP1) | x |  |  |  |  | DB08012 | PMID: 1317142 |
| Pirodavir | EVA-71 | Capsid (VP1) | x |  |  |  |  | DB08012 | PMID: 25199773 |
| Pirodavir | CVB-2 | Capsid (VP1) | x |  |  |  |  | DB08012 | PMID: 1317142 |
| Pirodavir | CVB-3 | Capsid (VP1) | x |  |  |  |  | DB08012 | PMID: 1317142 |
| Pirodavir | CVB-4 | Capsid (VP1) | x |  |  |  |  | DB08012 | PMID: 1317142 |
| Pirodavir | CVB-5 | Capsid (VP1) | x |  |  |  |  | DB08012 | PMID: 1317142 |
| Pirodavir | CVB-6 | Capsid (VP1) | x |  |  |  |  | DB08012 | PMID: 1317142 |
| Pleconaril | EV1 | Capsid (VP1) |  | x |  |  |  | DB05105 | PMID: 38417531 |
| Pleconaril | EV6 | Capsid (VP1) |  | x |  |  |  | DB05105 | PMID: 10471549 |
| Pleconaril | EV7 | Capsid (VP1) | x |  |  |  |  | DB05105 | PMID: 10471549 |
| Pleconaril | EV11 | Capsid (VP1) |  | x |  |  | x | DB05105 | PMID: 39843710 |
| Pleconaril | EVA-71 | Capsid (VP1) |  | x |  |  |  | DB05105 | PMID: 38417531 |
| Pleconaril | CVA-9 | Capsid (VP1) |  |  | x |  |  | DB05105 | PMID: 10471549 |
| Pleconaril | CVA-13 | Capsid (VP1) | x |  |  |  |  | DB05105 | PMID: 39843710 |
| Pleconaril | CVB-1 | Capsid (VP1) | x |  |  |  | x | DB05105 | PMID: 31705692 |
| Pleconaril | CVB-2 | Capsid (VP1) | x |  |  |  |  | DB05105 | PMID: 31705692 |
| Pleconaril | CVB-4 | Capsid (VP1) | x |  |  |  |  | DB05105 | PMID: 31705692 |
| Pleconaril | CVB-5 | Capsid (VP1) |  | x |  |  |  | DB05105 | PMID: 39843710 |
| Pleconaril | CVB-6 | Capsid (VP1) | x |  |  |  |  | DB05105 | PMID: 39843710 |
| Pleconaril | HAV | Capsid (VP1) | x |  |  |  |  | DB05105 | PMID: 23528258 |
| Pocapavir | PV-1 | Capsid (VP1) | x |  |  |  |  | DB17736 | <https://doi.org/10.3389/av.2024.12361> |
| Pocapavir | EV6 | Capsid (VP1) | x |  |  |  |  | DB17736 | PMID: 9174174 |
| Pocapavir | EV7 | Capsid (VP1) | x |  |  |  |  | DB17736 | PMID: 9174174 |
| Pocapavir | EV11 | Capsid (VP1) | x |  |  |  |  | DB17736 | PMID: 9174174 |
| Pocapavir | CVA-9 | Capsid (VP1) | x |  |  |  |  | DB17736 | <https://doi.org/10.3389/av.2024.12361> |
| Pocapavir | CVB-2 | Capsid (VP1) | x |  |  |  |  | DB17736 | PMID: 9174174 |
| Pocapavir | CVB-3 | Capsid (VP1) | x |  |  |  |  | DB17736 | PMID: 9174174 |
| Pocapavir | CVB-4 | Capsid (VP1) | x |  |  |  |  | DB17736 | <https://doi.org/10.3389/av.2024.12361> |
| Remdesivir | EV1 | RdRp | x |  |  |  |  | DB14761 | PMID: 38417531 |
| Remdesivir | EV6 | RdRp | x |  |  |  |  | DB14761 | PMID: 38417531 |
| Remdesivir | EV11 | RdRp | x |  |  |  |  | DB14761 | PMID: 38417531 |
| Remdesivir | EVA-71 | RdRp |  | x |  |  |  | DB14761 | PMID: 32595613 |
| Remdesivir | CVB-3 | RdRp | x |  |  |  |  | DB14761 | PMID: 32595613 |
| Remdesivir | CVB-5 | RdRp | x |  |  |  |  | DB14761 | PMID: 38417531 |
| Ribavirin | EVA-71 | RNA synthesis |  |  | x |  |  | DB00811 | PMID: 18279075 |
| Ribavirin | CVB-1 | RNA synthesis | x |  |  |  |  | DB00811 | PMID: 31705692 |
| Ribavirin | CVB-2 | RNA synthesis | x |  |  |  |  | DB00811 | PMID: 31705692 |
| Ribavirin | CVB-3 | RNA synthesis | x |  |  |  |  | DB00811 | PMID: 31705692 |
| Ribavirin | CVB-4 | RNA synthesis | x |  |  |  |  | DB00811 | PMID: 31705692 |
| Ribavirin | CVB-5 | RNA synthesis | x |  |  |  |  | DB00811 | PMID: 31705692 |
| Ribavirin | CVB-6 | RNA synthesis | x |  |  |  |  | DB00811 | PMID: 31705692 |
| Ribavirin | HAV | RNA synthesis | x |  |  |  |  | DB00811 | PMID: 35269774 |
| Rupintrivir | PV-1 | 3C protease | x |  |  |  |  | DB05102 | PMID: 21466823 |
| Rupintrivir | EV1 | 3C protease | x |  |  |  |  | DB05102 | PMID: 38417531 |
| Rupintrivir | EV6 | 3C protease | x |  |  |  |  | DB05102 | PMID: 38417531 |
| Rupintrivir | EV11 | 3C protease | x |  |  |  |  | DB05102 | PMID: 38417531 |
| Rupintrivir | EVA-71 | 3C protease | x |  |  |  |  | DB05102 | PMID: 25199773 |
| Rupintrivir | CVB-5 | 3C protease | x |  |  |  |  | DB05102 | PMID: 38417531 |
| RYL-634 | EVA-71 | DHODH | x |  |  |  |  | N/A | PMID: 30938999 |
| Sofosbuvir | EVA-71 | RdRp |  |  | x |  |  | DB08934 | PMID: 32424333 |
| Sofosbuvir | HAV | RdRp | x |  |  |  |  | DB08934 | PMID: 29653132 |
| Suramin | EVA-71 | Capsid |  |  | x |  |  | DB04786 | PMID: 26038755 |
| Suramin | CVA-9 | Capsid | x |  |  |  |  | DB04786 | PMID: 28218309 |
| T-00127-HEV1 | EVA-71 | PI4KIIIß | x |  |  |  |  | N/A | PMID: 21177810 |
| T-00127-HEV1 | CVB-3 | PI4KIIIß | x |  |  |  |  | N/A | PMID: 21177810 |
| Vapendavir | EV1 | Capsid (VP1) | x |  |  |  |  | DB05181 | PMID: 38417531 |
| Vapendavir | EV6 | Capsid (VP1) | x |  |  |  |  | DB05181 | [PMID: 38417531](https://pubmed.ncbi.nlm.nih.gov/39843710/) |
| Vapendavir | EV11 | Capsid (VP1) | x |  |  |  |  | DB05181 | [PMID: 39843710](https://pubmed.ncbi.nlm.nih.gov/39843710/) |
| Vapendavir | EVA-71 | Capsid (VP1) | x |  |  |  |  | DB05181 | PMID: 25199773 |
| Vapendavir | CVB-5 | Capsid (VP1) | x |  |  |  |  | DB05181 | [PMID: 39843710](https://pubmed.ncbi.nlm.nih.gov/39843710/) |
| Vemurafenib | EV1 | Unknown | x |  |  |  |  | DB08881 | PMID: 33080984 |
| Vemurafenib | EV6 | Unknown | x |  |  |  |  | DB08881 | PMID: 38417531 |
| Vemurafenib | EVA-71 | Unknown | x |  |  |  |  | DB08881 | PMID: 36145288 |
| Vemurafenib | CVA-9 | Unknown | x |  |  |  |  | DB08881 | PMID: 37436162 |
| Vemurafenib | CVB-1 | Unknown | x |  |  |  |  | DB08881 | PMID: 37436162 |
| Vemurafenib | CVB-2 | Unknown | x |  |  |  |  | DB08881 | PMID: 37436162 |
| Vemurafenib | CVB-3 | Unknown | x |  |  |  |  | DB08881 | PMID: 37436162 |
| Vemurafenib | CVB-4 | Unknown |  |  | x |  |  | DB08881 | PMID: 37436162 |
| Vemurafenib | CVB-5 | Unknown | x |  |  |  |  | DB08881 | PMID: 37436162 |
| Vemurafenib | CVB-6 | Unknown | x |  |  |  |  | DB08881 | PMID: 37436162 |
| Z10325150 | HAV | 3C protease | x |  |  |  |  | N/A | PMID: 35682728 |

Table S8. Combinations of antienterovierals.

| **Virus** | **Drug 1** | **Drug 2** | **Clinical phase** | **Line/Animal Model** | **Effect (A/S)** | **Reference** |
| --- | --- | --- | --- | --- | --- | --- |
| EV1 | Anisomycin | IMP-1088 | Preclinical | A549 | S | PMID: 38417531 |
| EV1 | Anisomycin | Rupintrivir | Preclinical | A549 | A | PMID: 36146673 |
| EV1 | Anisomycin | Vemurafenib | Preclinical | A549 | S | PMID: 33080984 |
| EV1 | Anisomycin | Vapendavir | Preclinical | A549 | A | PMID: 38417531 |
| EV1 | Anisomycin | Remdesivir | Preclinical | A549 | A | PMID: 38417531 |
| EV1 | Anisomycin | Emetine | Preclinical | A549 | A | PMID: 38417531 |
| EV1 | Anisomycin | Pleconaril | Preclinical | A549 | S | PMID: 38417531 |
| EV1 | Anisomycin | Enviroxime | Preclinical | A549 | S | PMID: 38417531 |
| EV1 | Anisomycin | Cycloheximide | Preclinical | A549 | A | PMID: 38417531 |
| EV1 | Cycloheximide | IMP-1088 | Preclinical | A549 | A | PMID: 38417531 |
| EV1 | Cycloheximide | Rupintrivir | Preclinical | A549 | S | PMID: 36146673 |
| EV1 | Cycloheximide | Vemurafenib | Preclinical | A549 | S | PMID: 33080984 |
| EV1 | Cycloheximide | Pleconaril | Preclinical | A549 | S | PMID: 38417531 |
| EV1 | Cycloheximide | Vapendavir | Preclinical | A549 | S | PMID: 38417531 |
| EV1 | Cycloheximide | Remdesivir | Preclinical | A549 | S | PMID: 38417531 |
| EV1 | Cycloheximide | Enviroxime | Preclinical | A549 | S | PMID: 38417531 |
| EV1 | Emetine | IMP-1088 | Preclinical | A549 | A | PMID: 38417531 |
| EV1 | Emetine | Pleconaril | Preclinical | A549 | S | PMID: 38417531 |
| EV1 | Emetine | Rupintrivir | Preclinical | A549 | A | PMID: 36146673 |
| EV1 | Emetine | Vemurafenib | Preclinical | A549 | S | PMID: 33080984 |
| EV1 | Emetine | Enviroxime | Preclinical | A549 | S | PMID: 38417531 |
| EV1 | Enviroxime | IMP-1088 | Preclinical | A549 | A | PMID: 38417531 |
| EV1 | Enviroxime | Pleconaril | Preclinical | A549 | S | PMID: 38417531 |
| PV | Enviroxime | Guanidine | Preclinical | FL cells | S | PMID: 10757231 |
| PV | Enviroxime | Ribavirin | Preclinical | Unknown | A | PMID: 21466823 |
| EV1 | Enviroxime | Rupintrivir | Preclinical | A549 | S | PMID: 38417531 |
| EV-A71 | Enviroxime | Rupintrivir | Preclinical | Unknown | A | PMID:21466823 |
| HRV-B14 | Enviroxime | Rupintrivir | Preclinical | Unknown | A | PMID:21466823 |
| PV | Enviroxime | Rupintrivir | Preclinical | Unknown | A | PMID:21466823 |
| EV-A71 | Enviroxime | Vapendavir | Preclinical | Unknown | A | PMID: 21466823 |
| HRV-B14 | Enviroxime | Vapendavir | Preclinical | Unknown | A | PMID: 21466823 |
| PV | Enviroxime | Vapendavir | Preclinical | Unknown | A | PMID: 21466823 |
| EV-A71 | Favipiravir | Suramin | Preclinical | RD;Vero cells | S | PMID: 27353263 |
| CVB-1 | MDL-860 | Oxoglaucine | Preclinical | HEp-2 cells | A | PMID: 34978843 |
| CVB-3 | MDL-860 | Oxoglaucine | Preclinical | HEp-2 cells | S | PMID: 34978843 |
| CVB-1 | MDL-860 | Guanidine | Preclinical | HEp-2 cells | S | PMID: 34978843 |
| CVB-3 | MDL-860 | Guanidine | Preclinical | HEp-2 cells | S | PMID: 34978843 |
| EV-A71 | NK-1.9k | NITD008 | Preclinical | RD cells | S | PMID: 28063993 |
| EV-A71 | NK-1.9k | ALD | Preclinical | RD cells | S | PMID: 28063993 |
| EV1 | Pleconaril | IMP-1088 | Preclinical | A549 | S | PMID: 38417531 |
| CVB-1 | Pleconaril | Guanidine | Preclinical | HEp-2 cells | S | PMID: 34978843 |
| CBV-3 (W) | Pleconaril | Guanidine | Preclinical | HEp-2 cells | S | PMID: 34978843 |
| CVB-1 | Pleconaril | MDL-860 | Preclinical | HEp-2 cells | A | PMID: 34978843 |
| CVB-3 (W) | Pleconaril | MDL-860 | Preclinical | HEp-2 cells | S | PMID: 34978843 |
| CVB-1 | Pleconaril | Oxoglaucine | Preclinical | HEp-2 cells | A | PMID: 34978843 |
| CVB-3 (W) | Pleconaril | Oxoglaucine | Preclinical | HEp-2 cells | A | PMID: 34978843 |
| EV1 | Pleconaril | Rupintrivir | Preclinical | A549 | S | PMID: 36146673 |
| EV1 | Pleconaril | Vemurafenib | Preclinical | A549 | S | PMID: 36146673 |
| PV-1 | Pocapavir | AG-7404 | Preclinical | HeLa cells | S | PMID: 23499651 |
| PV-1 | Pocapavir | Vapendavir | Preclinical | HeLa cells | A | PMID: 23499651 |
| PV-1 | Pocapavir | Guanidine | Preclinical | HEp-2 cells | A | doi: 10.3389/av.2024.12361 |
| CVA-9 | Pocapavir | Guanidine | Preclinical | HEp-2 cells | A | doi: 10.3389/av.2024.12361 |
| CVB-4 | Pocapavir | Guanidine | Preclinical | HEp-2 cells | S | doi: 10.3389/av.2024.12361 |
| PV-1 | Pocapavir | MDL-860 | Preclinical | HEp-2 cells | S | doi: 10.3389/av.2024.12361 |
| CVA-9 | Pocapavir | MDL-860 | Preclinical | HEp-2 cells | A | doi: 10.3389/av.2024.12361 |
| CVB-4 | Pocapavir | MDL-860 | Preclinical | HEp-2 cells | S | doi: 10.3389/av.2024.12361 |
| PV-1 | Pocapavir | Oxoglaucine | Preclinical | HEp-2 cells | S | doi: 10.3389/av.2024.12361 |
| CV-A9 | Pocapavir | Oxoglaucine | Preclinical | HEp-2 cells | S | doi: 10.3389/av.2024.12361 |
| CVB-4 | Pocapavir | Oxoglaucine | Preclinical | HEp-2 cells | S | doi: 10.3389/av.2024.12361 |
| CVA-9 | Pocapavir | Pleconaril | Preclinical | HEp-2 cells | A | doi: 10.3389/av.2024.12361 |
| CVB-4 | Pocapavir | Pleconaril | Preclinical | HEp-2 cells | S | doi: 10.3389/av.2024.12361 |
| EV1 | Remdesivir | IMP-1088 | Preclinical | A549 | A | PMID: 38417531 |
| EV1 | Remdesivir | Rupintrivir | Preclinical | A549 | A | PMID: 36146673 |
| EV1 | Remdesivir | Vemurafenib | Preclinical | A549 | S | PMID: 38417531 |
| EV1 | Remdesivir | Vapendavir | Preclinical | A549 | S | PMID: 38417531 |
| EV-A71 | Ribavirin | Gemcitabine | Preclinical | Vero cells | S | PMID: 26526589 |
| CVB-3 | Ribavirin | Gemcitabine | Preclinical | Vero cells | S | PMID: 26526589 |
| EV1 | Rupintrivir | IMP-1088 | Preclinical | A549 | S | PMID: 38417531 |
| EV-A71 | Rupintrivir | Favipiravir | Preclinical | RD;Vero cells | S | PMID: 27353263 |
| EV-A71 | Rupintrivir | Itraconazole | Preclinical | RD;Vero cells | S | PMID: 27353263 |
| PV | Rupintrivir | Ribavirin | Preclinical | Unknown | A | PMID: 21466823 |
| EV-A71 | Rupintrivir | Suramin | Preclinical | RD;Vero cells | A | PMID: 27353263 |
| EV-A71 | Rupintrivir | NITD008 | Preclinical | RD cells | S | PMID: 25446894 |
| EV-A71 | Rupintrivir | Vapendavir | Preclinical | Unknown | A | PMID:21466823 |
| HRV-B14 | Rupintrivir | Vapendavir | Preclinical | Unknown | A | PMID:21466823 |
| PV | Rupintrivir | Vapendavir | Preclinical | Unknown | A | PMID:21466823 |
| EV1 | Rupintrivir | Vapendavir | Preclinical | A549 | A | PMID: 38417531 |
| EV1 | Rupintrivir | Vemurafenib | Preclinical | A549 | S | PMID: 36146673 |
| EV1 | Vapendavir | IMP-1088 | Preclinical | A549 | A | PMID: 38417531 |
| PV | Vapendavir | AG-7404 | Preclinical | HeLa cells | S | PMID: 23499651 |
| PV | Vapendavir | Ribavirin | Preclinical | Unknown | A | PMID: 21466823 |
| EV1 | Vapendavir | Vemurafenib | Preclinical | A549 | S | PMID: 38417531 |
| EV1 | Vemurafenib | Gemcitabine | Preclinical | A549 | S | PMID: 33080984 |
| EV1 | Vemurafenib | Homoharringtonine | Preclinical | A549 | S | PMID: 33080984 |
| EV1 | Vemurafenib | Obatoclax | Preclinical | A549 | S | PMID: 33080984 |


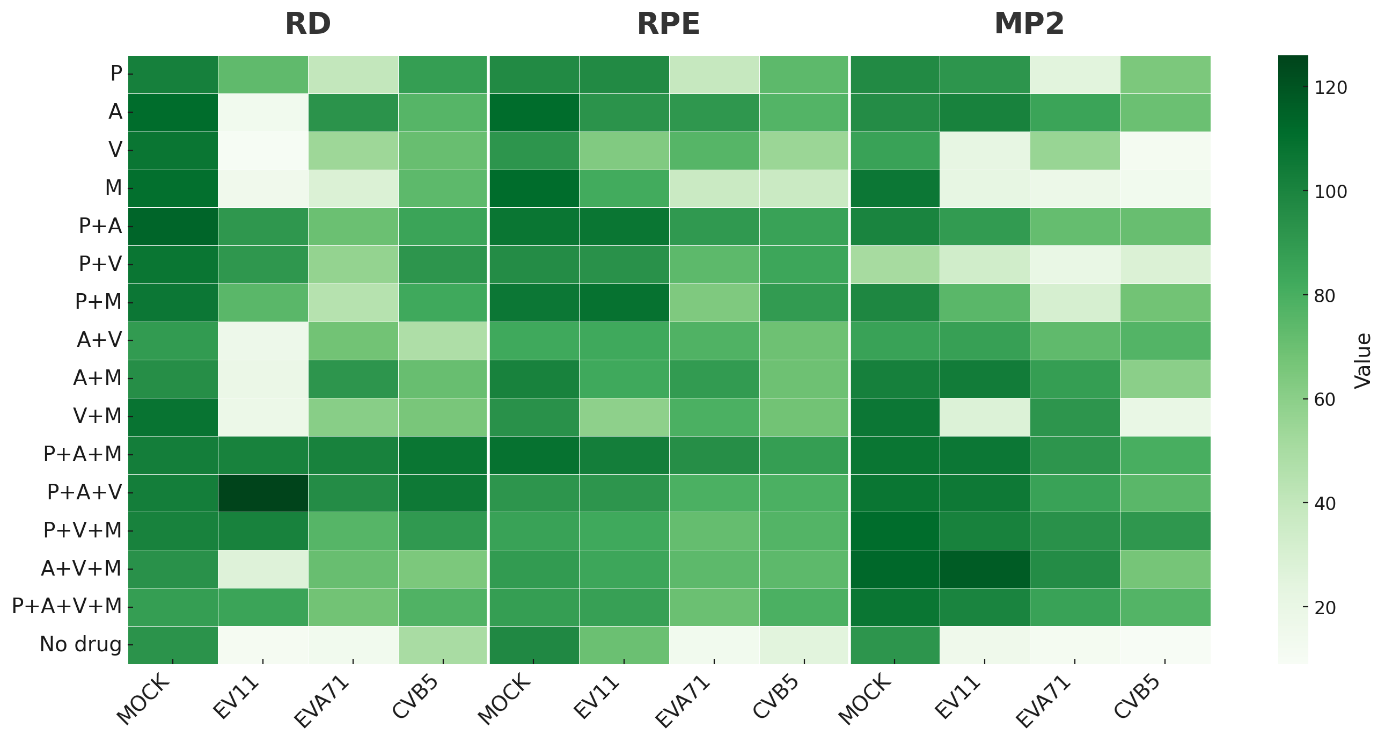


**Fig. S1**. Anti-enteroviral effect of pleconaril (P), AG7404 (A), vemurafenib (V), mindeudesivir (M), and their combinations in RD, RPE and MP2 cell cultures. Cells were treated with 0.2 μM P, 1 μM A, 5 μM V or their combinations, and infected with enteroviruses (100 TCID_50_). After 72 h, cell viability was determined using a CTG assay. Mean, n=3.

**
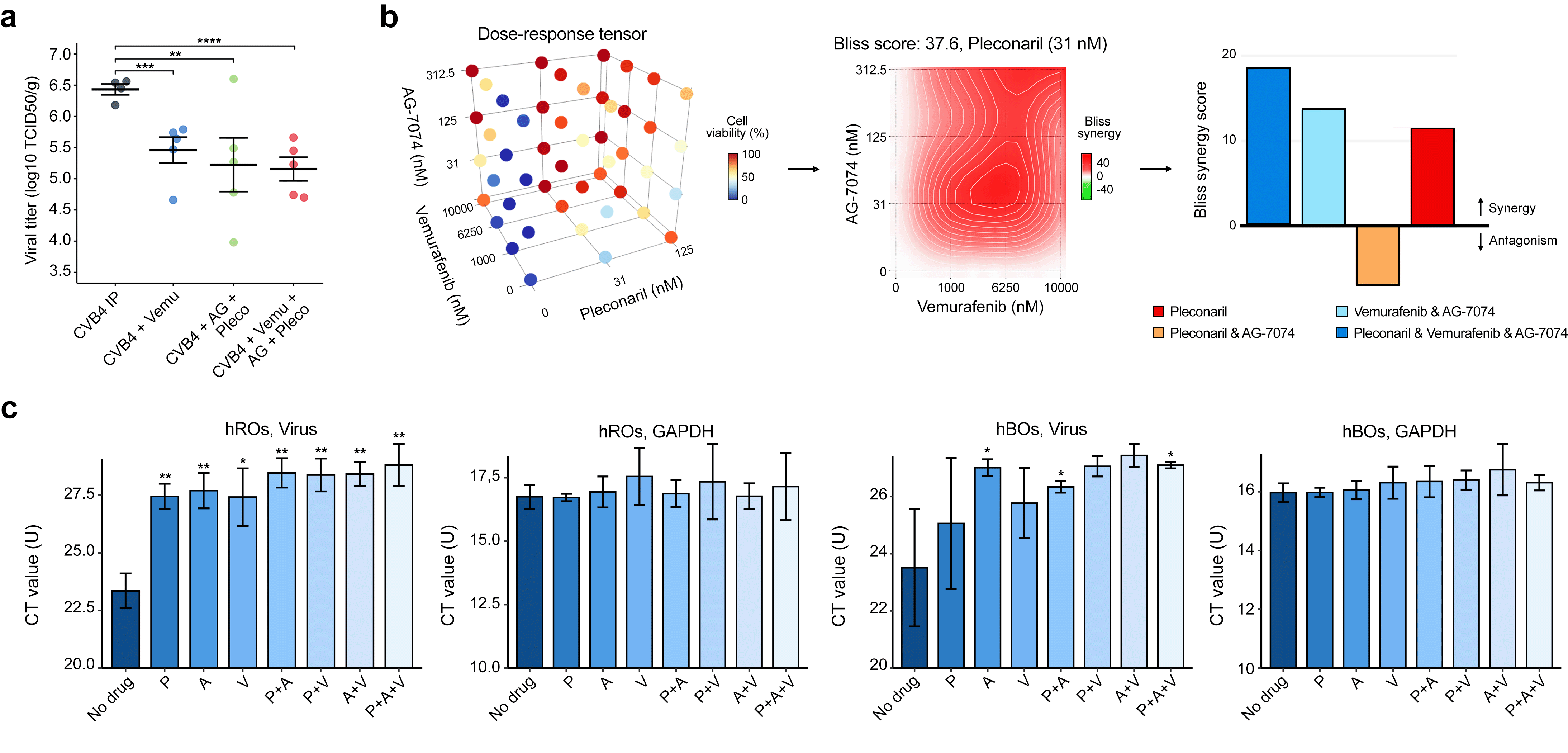
**

**Fig. S2**. Retinal (hROs) and brain (hBOs) organoids were treated with 0.2 μM P, 1 μM A, 5 μM V or their combinations, and infected with EV11 (100 TCID50). After 72 h, culture supernatants were collected, and RNA was extracted. Viral and host RNA was analyzed by RT-qPCR. CT values are shown (mean ± SD; n = 3). *p<0.1, ** p<0.05, ***p<0.01, ****p<0.001.
